# “Naked Nickel”‐Catalyzed Heteroaryl–Heteroaryl Suzuki–Miyaura Coupling

**DOI:** 10.1002/anie.202424051

**Published:** 2025-03-12

**Authors:** Rakan Saeb, Byeongdo Roh, Josep Cornella

**Affiliations:** ^1^ Max‐Planck‐Institut für Kohlenforschung Kaiser‐Wilhelm‐Platz 1 45470 Mülheim an der Ruhr Germany

**Keywords:** Boron, Cross‐coupling, Heterocycles, Nickel, Synthetic methods

## Abstract

In this article, we report that the air‐stable “naked nickel”, [Ni(^4‐CF3^stb)_3_], is a competent catalyst in the catalytic Suzuki–Miyaura cross‐coupling reaction (SMC) between heteroaryl bromides and heteroaromatic boron‐based nucleophiles. The catalytic system is characterized by its ability to avoid decomposition or deactivation in the presence of multiple Lewis basic sites. The protocol permits the formation of C‒C bonds between two heteroaryl moieties in the absence of complex exogenous ligands, thus minimizing screening procedures and simplifying reaction setups. This method accommodates combinations of distinct 6‐membered heteroaryl bromides and 5‐ and 6‐membered heterocyclic *B*‐based nucleophiles.

## Graphical abstract

We report that an air‐stable “naked nickel”, Ni(^4‐CF3^stb)_3_, can efficiently catalyze heteroaryl–heteroaryl Suzuki–Miyaura cross‐coupling (SMC) reactions in the absence of exogenous ligands. This protocol permits the construction of >15 different heterobiaryls containing multiple Lewis basic heteroatoms from a broad set of 6‐membered heteroaryl bromides with 5‐ and 6‐membered heterocyclic *B*‐nucleophiles.

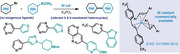

The catalytic construction of C─C bonds *via* the coupling of (hetero)aryl (pseudo)halides with *B*‐based nucleophiles (Suzuki–Miyaura cross‐coupling, SMC coupling) has had a fundamental impact on our society. In fact, the SMC coupling represents the most frequently used catalytic transformation in drug discovery campaigns.^[^ ^1^, ^2^ ^]^ A recent survey revealed that 82% of new FDA‐approved drugs contain at least one *N*‐heterocyclic unit, with pyridine being the most recurrent heterocycle.^[^ ^3^ ^]^ It is, therefore, no surprise that the construction of C(sp^2^)─C(sp^2^) bonds between two heterocyclic motifs *via* a SMC is of significant interest. However, this transformation is not trivial as the presence of Lewis basic sites in the substrates and products can poison the catalyst through coordination.^[^ ^4^, ^5^, ^6^ ^]^ Traditionally, this catalytic transformation has relied on the use of Pd catalysts equipped with bulky and crafted ligands.^[^ ^7^, ^8^, ^9^, ^10^, ^11^, ^12^, ^13^, ^14^ ^]^ With the goal of transitioning to more sustainable processes, strategies based on Ni have become of high interest as replacement for Pd.^[^ ^15^, ^16^, ^17^ ^]^ In 2012 and 2013, the Hartwig and Garg groups reported the first Ni‐catalyzed SMC couplings that would permit the formation of heterobiaryls (Figure 1a). In both, the catalytic reaction relied on the application of preligated Ni(phosphine) complexes.^[^ ^18^, ^19^ ^]^ These seminal works set the stage for a myriad of subsequent reports on mechanistic studies, the development of even more fine‐tuned ligands and specialized catalytic systems that advanced the area of Ni‐catalyzed SMC (Figure 1a).^[^ ^4^, ^5^, ^6^, ^20^, ^21^, ^22^, ^23^, ^24^, ^25^, ^26^, ^27^, ^28^, ^29^, ^30^, ^31^, ^32^, ^33^, ^34^, ^35^, ^36^ ^]^ Despite the advances, the use of expensive and multistep *P*‐, *N*‐ and NHC‐based ligands is still required for a successful coupling. As a result, practitioners either require a detailed knowledge of reactivity profiles to circumvent challenges associated with different ligand classes or rely on extensive ligand screening assays (HTE).^[^ ^6^, ^29^, ^30^, ^32^, ^37^, ^38^, ^39^, ^40^ ^]^ Hence, a simplified catalytic system where no exogenous ligands are required would increase simplicity in reaction setups and therefore, would be highly coveted. Even though examples of SMC in the absence of exogenous ligands have been reported,^[^ ^41^, ^42^, ^43^, ^44^ ^]^ their scope is limited when attempting heterobiaryl synthesis. So far, only the cross‐coupling of 2‐bromothiophene with 3‐thienylboronic acid and 4‐pyridinylboronic acid have been described.

**Figure 1:**
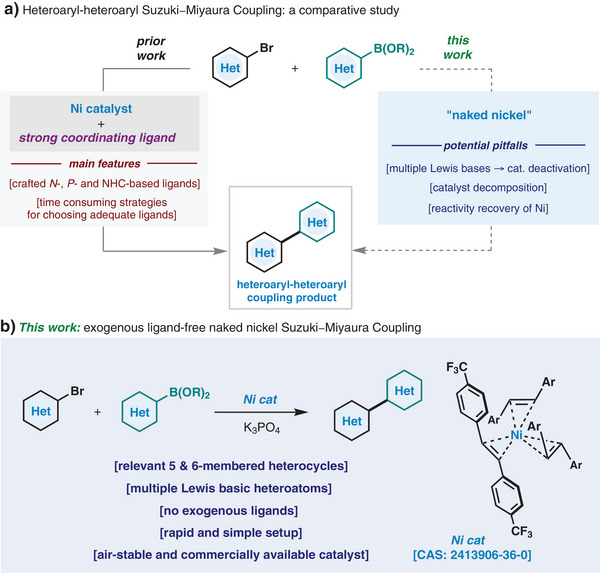
a) Traditional approaches for Ni‐catalyzed Suzuki‒Miyaura cross‐coupling (SMC) between two heteroaryl units. b) This work: a simple “naked‐nickel”‐catalyzed approach for heteroaryl–heteroaryl SMC coupling.

With the aim of providing simple and more accessible methods, our group has recently reported a Buchwald–Hartwig amination reaction, where an air‐stable “naked nickel” catalyst could catalyze the formation of C─N bonds between heteroaryl bromides and various *N*‐based nucleophiles in the absence of ancillary ligands.^[^ ^45^ ^]^ We speculated that such an approach could be extended to Suzuki–Miyaura cross‐coupling reactions, thus enabling facile construction of heterobiaryl scaffolds.^[^ ^46^, ^47^, ^48^, ^49^, ^50^, ^51^, ^52^, ^53^ ^]^

Herein, we report that the commercially available Ni(^4‐CF3^stb)_3_ (CAS: 2413906‐36‐0), a robust and air‐stable Ni(0)‐complex developed by our group,^[^ ^54^ ^]^ can catalyze the SMC coupling of a broad range of heteroaryl bromides with heteroaromatic boron‐based nucleophiles without the necessity of applying ancillary ligands (Figure 1b).

Our initial attempts involved the coupling of 3‐bromopyridine (**1**) with 3‐thienylboronic acid (**2**) in the presence of 10 mol% of Ni(^4‐CF3^stb)_3_. After an extensive optimization of the reaction parameters, we identified K_3_PO_4_ as the suitable base in DMA (0.5 M) at 60 °C for 16 h, resulting in 86% isolated yield of the coupling product (**3**) (Table 1, Entry 1). By switching to Ni(^4‐tBu^stb)_3_, a slight decrease in yield was observed (Entry 2). As shown in Entries 3 and 4, other *B*‐based nucleophiles such as pinacol esters (Bpin) or more robust 1,1,2,2‐tetraethylene glycol esters (BEpin)^[^ ^55^ ^]^ were amenable to coupling, providing 86% and 44% of **3**, respectively. However, the parent potassium trifluoroborate (BF_3_K) was unsuccessful under the optimized conditions (Entry 5). Reduction of the catalyst loading to 5 mol% had a detrimental effect in the outcome of the reaction; yet, compound **3** could still be obtained in a satisfactory 59% yield (Entry 6). Replacing Ni(^4‐CF3^stb)_3_ by NiCl_2_⋅6H_2_O afforded 18% of **3,** whereas the addition of 30 mol% of ^4‐CF3^stb to the Ni(II) salt did not improve the reactivity (Entries 7 and 8). This reactivity was also observed previously in the context of a “naked nickel”‐catalyzed Buchwald–Hartwig coupling.^[^ ^45^ ^]^ Wilke's “naked nickel” Ni(COD)_2_^[^ ^56^ ^]^ afforded the desired product in good yield when handling the catalyst under a strictly inert atmosphere (Entry 9). In contrast, the utilization of an alternative air‐stable Ni(0)‐source, such as Ni(COD)(DQ),^[^ ^57^ ^]^ provided lower yields (Entry 10). Finally, a control experiment confirmed the requirement of Ni in this SMC coupling (Table 1, Entry 11).

**Table 1:** Optimization of the “naked nickel”‐catalyzed coupling of 3‐bromopyridine with 3‐thienylboronic acid.

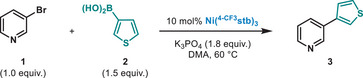		
Entry	Deviations from above	Yield in % of 3^a)^
1	None	93 (86)^b)^
2	Ni(^4‐tBu^stb)_3_	83
3	With 3‐thienyl‐Bpin	86
4	With 3‐thienyl‐BEpin	44
5	With 3‐thienyl‐BF_3_K	n.d.
6	5 mol% Ni(^4‐CF3^stb)_3_	59
7	NiCl_2_⋅6H_2_O	18
8	NiCl_2_⋅6H_2_O, 30 mol% ^4‐CF3^stb	18
9	Ni(COD)_2_	73
10	Ni(COD)(DQ)	26
11	No [Ni]	n.d.

^a)^ ^1^H NMR yield as determined by using 1,3,5‐trimethoxybenzene as internal standard.

^b)^ Isolated yield (0.3 mmol scale). ^4‐tBu^stb, (*E*)‐1,2‐bis(4‐(*tert*‐butyl)phenyl)ethene; ^4‐CF3^stb, (*E*)‐1,2‐bis(4‐(trifluoromethyl)phenyl)ethene; COD, 1,5‐cyclooctadiene; DQ, duroquinone; n.d., not detected.

With the optimized conditions in hand, we explored the generality of the protocol regarding heteroaryl bromides using **2** as model nucleophile (Figure 2). Besides **1**, a pyridine derivative bearing a methyl ester underwent smooth coupling (**4**), and additional ester functionalities in **14** and **30** also could be accommodated. The protocol also permits the coupling of a 4‐bromopyridine (**5**) in moderate yield. In contrast to a previous report,^[^ ^44^ ^]^ the coupling of 2‐bromopyridines was feasible under our optimized protocol. The coupling of a 2‐bromopyridine derivative with a 5‐CF_3_ group proceeded smoothly giving **6** in good yield, whereas 6‐CN substituted 2‐bromopyridine resulted in product formation in moderate yield (**7**). 6‐Membered heterocycles containing two Lewis basic heteroatoms were also amenable with our protocol, permitting the coupling of pyrimidines and pyrazines (**8** and **9**) in high yield. The coupling of 4‐bromoisoquinoline with 3‐thienylboronic acid delivered the desired product in excellent yield (**10**). Its structural isomer, 4‐bromoquinoline, also underwent smooth coupling (**11**). Fused heterocycles such as 3‐bromo‐1,5‐naphthyridine also afforded the desired C‒C product in excellent yield (**12**). Furthermore, we could demonstrate the coupling of a 4‐chloroquinazoline derivative (**13**) in moderate yield, although slightly elevated temperatures were required. Finally, a pyridine connected to a protected amino acid (Boc‐Tyr‐OMe) underwent successful coupling (**14**) despite the presence of multiple Lewis basic heteroatoms, esters, or a carbamate. At present, the coupling of 5‐membered heteroaryl bromides falls beyond the scope of the current study (see Supporting Information). Next, we investigated the scope of boron‐based nucleophiles that could be used in our protocol (Figure 2). Besides 3‐thienylboronic acid (**3**), 3‐furanylboronic acid was found to be an excellent coupling partner (**15**). Additionally, bicyclic heterocycles such as 3‐benzothiophene (**16**) and 2‐benzofuran (**17**) could be satisfactorily coupled with **1** in high yields. Furthermore, *tert*‐butyl 4‐(4‐(4,4,5,5‐tetramethyl‐1,3,2‐dioxaborolan‐2‐yl)‐1*H*‐pyrazol‐1‐yl)piperidine‐1‐carboxylate could be efficiently coupled with 3‐bromopyridine, delivering **18** in good yield. The coupling of **1** with unprotected 5‐indolylboronic acid also afforded the desired heterobiaryl **19** in good yield. An indazole bearing an *o*‐Me group in respect to the Bpin moiety underwent coupling in moderate yield (**20**). Next, we turned our attention to the scope of B‐nucleophiles derived from 6‐membered heterocycles, a coupling that has proven difficult in previous studies.^[^ ^4^, ^18^, ^36^ ^]^ 3‐Quinolineboronic acid could be coupled in good yield (**21**). More interestingly, the coupling of pyridine‐based boronic acids turned out to be feasible with our protocol. 6‐Methoxy‐3‐pyridinylboronic acid also underwent efficient coupling to the desired product (**22**). When increasing the steric pressure, as in the case of 2‐methoxy‐3‐pyridinylboronic acid, the coupling remained possible, albeit in slightly reduced yields (**23**). Decreasing the electron density on the boronic acid also resulted in coupling, as demonstrated by **24**. Furthermore, a 3‐pyridinylboronic ester bearing an unprotected 6‐piperidinol substituent was amenable for cross‐coupling with our protocol, resulting in the formation of **25** in moderate yield. Finally, a *N*‐methylated pyrrolo[2,3*b*]pyridine could also be applied in a SMC coupling giving the desired product in moderate yield (**26**). Overall, we demonstrated the coupling of 18 types of distinct heterocycles, with a “naked nickel” catalyst, thus overcoming common challenges such as the coupling of pyridinylboronic acids^[^ ^4^, ^18^, ^36^ ^]^ or catalyst poisoning.^[^ ^4^, ^5^, ^6^ ^]^

**Figure 2:**
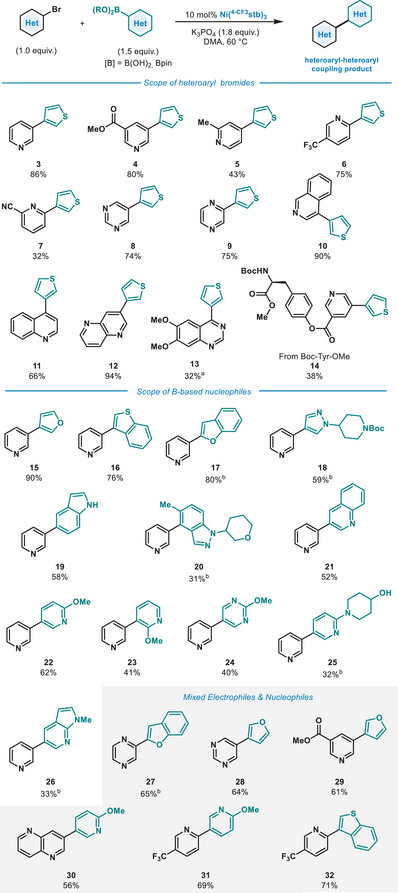
Scope of the Suzuki–Miyaura cross‐coupling protocol. Reaction conditions: heteroaryl bromide (1.0 equiv.), heteroaryl boronic acid (1.5 equiv.), K_3_PO_4_ (1.8 equiv.), Ni(^4‐CF3^stb)_3_ (10 mol%), DMA (0.5 M), 60 °C, 16 h, 0.3 mmol scale. Yields represent isolated yields. ^a)^From heteroaryl chloride, 100 °C. ^b)^From heteroaryl boronic acid pinacol ester.

During the exploration of the scope in cross‐couplings, it is common practice to select one coupling partner and vary the nature of the other and *vice* *versa*. However, to provide a more representative picture of the feasibility of the protocol, we performed cross experiments, by mixing various *B*‐based nucleophiles and bromides (Figure 2, grey background). To this end, the coupling between 2‐bromopyrazine and benzofuran bearing a 2‐Bpin unit was attempted, resulting in efficient cross‐coupling (**27**). Additionally, 3‐furanylboronic acid could be smoothly coupled with 5‐bromopyrimidine as well as methyl 5‐bromonicotinate (**28** and **29**) in good yield. Furthermore, both 3‐bromo‐1,5‐naphthyridine and 2‐bromo‐5‐(trifluoromethyl)pyridine were demonstrated to be good coupling partners for 6‐methoxy‐3‐pyrdinylboronic acid (**30** and **31**). Finally, 2‐bromo‐5‐(trifluoro‐methyl)pyridine underwent efficient coupling with benzo[*b*]thien‐2‐ylboronic acid, resulting in the formation of the desired product (**32**) in high yield. We believe these last experiments truly highlight the potential of the methodology.

Considering the impact and usage of SMC in the many different fields of research, a simple reaction set‐up was deemed to be essential. The reaction set‐up does not require the use of dry K_3_PO_4_ nor is affected by the presence of 2.0 equiv. of H_2_O in the reaction mixture (Figure 3a, Path a). Moreover, when performing the coupling between **1** and **2** under air with DMA—stored on the benchtop without degassing nor predrying—the yield plummeted to 25% (Figure 3a, Path b). Nonetheless, a simple Ar flushing of the reaction vessel was sufficient to fully restore the catalytic activity, giving rise to **3** in 89% yield (Figure 3a, Path b). Finally, to highlight the translational potential of the method, 4‐bromoisoquinoline was reacted on a 5.0 mmol scale (1.0 g scale) with 3‐thienylboronic acid. We demonstrate that for this particular case, a reduced catalyst loading of 1 mol% Ni permitted the synthesis of **10** in high yield, thus representing a >16‐fold increase in scale (Figure 3b). Although successful in this particular case, the reduction of catalyst loading is not general (see Supporting Information).

**Figure 3:**
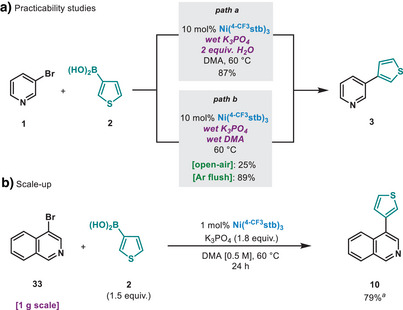
a) Practicability and zero‐precaution experiments. b) Scale‐up experiment. Yields determined by ^1^H NMR, using 1,3,5‐trimethoxybenzene as internal standard. ^a)^Isolated yield.

In conclusion, we have developed a protocol for a “naked nickel”‐catalyzed heteroaryl–heteroaryl Suzuki–Miyaura cross‐coupling reaction under mild conditions. The protocol is characterized by a simple reaction setup and the application of an air‐stable Ni(0)‐catalyst in the absence of exogenous ligands. It enables the coupling of heteroaryl bromides with heteroaromatic *B*‐based nucleophiles, resulting in the formation of >15 classes of heterobiaryls bearing multiple Lewis basic heteroatoms. To the best of our knowledge, this protocol represents the first example of a general SMC coupling in the absence of donating ligands that gives streamlined access to a broad scope of heterobiaryls. Although these results do not aim at replacing current strategies for SMC couplings, they serve as a blueprint of reactivity on the potential of Ni–olefin complexes for the preparation of heterobiaryls.

## Conflict of Interests

The authors declare no conflict of interest.

## Supporting information



Supporting Information

Supporting Information

## Data Availability

The data that support the findings of this study are available in the Supporting Information of this article.
