# Supplementary material for: “Naked Nickel”‐Catalyzed Heteroaryl–Heteroaryl Suzuki–Miyaura Coupling
Source: Angew Chem Int Ed Engl. 2025 Mar 12;64(22):e202424051. doi: 10.1002/anie.202424051 (PMC12105700; doi:10.1002/anie.202424051)

Supporting Information

"Naked Nickel"-Catalyzed Heteroaryl-Heteroaryl Suzuki-Miyaura Cross-Coupling Reactions

Rakan Saeb, Byeongdo Roh and Josep Cornella*

Max-Planck-Institut für Kohlenforschung, Department of Organometallic Chemistry, Kaiser-Wilhelm-Platz 1, 45470, Mülheim an der Ruhr, North Rhine-Westphalia, Germany

Table of Contents

General Considerations S1

Ligand and Catalyst Preparation S2

Preparation of Starting Materials S4

General Procedure (GP) for the Heteroaryl-Heteroaryl Suzuki-Miyaura Cross-Coupling S6

Reaction Optimization........ S7

3-(Thiophen-3-yl)pyridine (**3**):........ S8

Methyl 5-(thiophen-3-yl)nicotinate (**4**): S8

2-Methyl-4-(thiophen-3-yl)pyridine (**5**):........ S9

2-(Thiophen-3-yl)-5-(trifluoromethyl)pyridine (**6**): S9

6-(Thiophen-3-yl)picolinonitrile (**7**):........ S10

5-(Thiophen-3-yl)pyrimidine (**8**):........ S10

2-(Thiophen-3-yl)pyrazine (**9**): S11

4-(Thiophen-3-yl)isoquinoline (**10**): S11

4-(Thiophen-3-yl)quinoline (**11**): S12

3-(Thiophen-3-yl)-1,5-naphthyridine (**12**): S12

6,7-Dimethoxy-4-(thiophen-3-yl)quinazoline (**13**): S13

(*S*)-4-(2-((*tert*-Butoxycarbonyl)amino)-3-methoxy-3-oxopropyl)phenyl 5-(thiophen-3-yl)nicotinate (**14**): S14

3-(Furan-3-yl)pyridine (**15**):........ S14

3-(Benzo[*b*]thiophen-3-yl)pyridine (**16**): S15

3-(Benzofuran-2-yl)pyridine (**17**): S15

tert-Butyl 4-(4-(pyridin-3-yl)-1*H*-pyrazol-1-yl)piperidine-1-carboxylate (**18**):........ S16

5-(Pyridin-3-yl)-1*H*-indole (**19**): S17

5-Methyl-4-(pyridin-3-yl)-1-(tetrahydro-2*H*-pyran-3-yl)-1*H*-indazole (**20**):........ S17

3-(Pyridin-3-yl)quinoline (**21**):........ S18

6-Methoxy-3,3'-bipyridine (**22**):........ S19

2-Methoxy-3,3'-bipyridine (**23**):........ S19

2-Methoxy-5-(pyridin-3-yl)pyrimidine (**24**): S20

1-([3,3'-Bipyridin]-6-yl)piperidin-4-ol (**25**): S20

1-Methyl-5-(pyridin-3-yl)-1*H*-pyrrolo[2,3-*b*]pyridine (**26**): S21

2-(Benzofuran-2-yl)pyrazine (**27**):........ S21

5-(Furan-3-yl)pyrimidine (**28**):........ S22

Methyl 5-(furan-3-yl)nicotinate (**29**):........ S22

3-(6-Methoxypyridin-3-yl)-1,5-naphthyridine (**30**): S23

6'-Methoxy-5-(trifluoromethyl)-2,3'-bipyridine (**31**): S24

2-(Benzo[*b*]thiophen-3-yl)-5-(trifluoromethyl)pyridine (**32**): S24

Limitations of the Scope S26

Addition of Water S27

Zero-Precaution Experiment: Reaction under Air S27

Zero-Precaution Experiment: Argon-Flush S28

Scale-Up of 4-(thiophen-3-yl)isoquinoline (**10**) S29

Scale-Up of 2-Methoxy-5-(pyridin-3-yl)pyrimidine (**24**): S29

Ligand-Exchange Study in Ni($^{4\text{-}CF_3}$stb)$_3$ with 3-bromopyridine: S30

References S34

NMR Spectra S35

General Considerations

Unless otherwise stated, all manipulations were performed using simple Schlenk techniques (weigh in solids, one cycle of vacuum/argon and then add liquids/solvent) under dry argon in heat gun-dried glassware. Unless otherwise stated all reactions were carried out using anhydrous solvents. Ni($^{4\text{-}CF3}$stb)$_3$ (tris(*trans*-1,2-bis(4-(trifluoromethyl)phenyl)ethene)nickel(0)), CAS: 2413906-36-0) was prepared according to a literature procedure, [1] and was stored in a screw cap vial under air in a freezer (−18 °C) and was handled open to air under ambient conditions. Unless otherwise noted, all other reagents were obtained from commercial suppliers and used without further purification. Anhydrous DMA was purchased from Sigma-Aldrich (250 mL, 99.8%, SureSeal) or Thermo Fisher (99.5%, AcroSeal). Column chromatography: Merck silica gel 60 (40–63 µm). Preparative thin layer chromatography (prepTLC): Merck PLC Silica gel 60 F_{254}, 1mm (20 × 20 cm). MS (EI): Finnigan MAT 8200 (70 eV), ESI-MS: ESQ 3000 (Bruker). Accurate mass determinations: Bruker APEX III FT-MS (7 T magnet), QExactiveGC (Thermo Fischer), or MAT 95 (Finnigan). Mass analyzer type: Quadrupol-Orbitrap. NMR spectra for characterization were recorded on a Bruker AVIII HD 300, or AVIII HD 400 (at 298–300 K, unless otherwise stated). IR spectra were recorded on an Alpha Platinum ATR instrument (Bruker); wavenumbers ($\tilde{\nu}$) in cm^{-1}. Melting points were recorded on a Büchi melting point apparatus, Model B-540 (Büchi, Switzerland) and are uncorrected. ^{1}H NMR spectra were referenced to the residual protons of the deuterated solvent (δ ($CDCl_3$) = 7.26 ppm), δ (Toluene-d_8) = 2.09 ppm) (degassed *via* 4 freeze-pump-thaw cycles, dried over 3Å MS, and stored in a Schlenk tube under argon). ^{13}C NMR spectra were referenced internally to the D-coupled ^{13}C resonances of the NMR solvent. Chemical shifts (δ) are given in ppm, relative to TMS (tetramethylsilane), and coupling constants (*J*) are provided in Hz. ^{19}F NMR spectra were referenced externally to the ^{19}F resonances of $CFCl_3$. Multiplicities are reported as (s = singlet, d = doublet, t = triplet, q = quartet, p = pentet, m = multiplet or unresolved, brs = broad signal).

Ligand and Catalyst Preparation

(*E*)-1,2-bis(4-(trifluoromethyl)phenyl)ethene ($^{4\text{-CF3}}$stb):

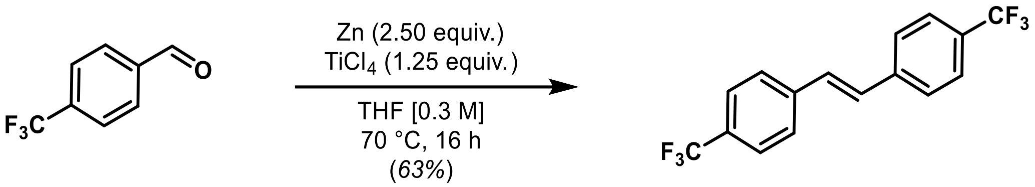

(*E*)-1,2-bis(4-(trifluoromethyl)phenyl)ethene was prepared according to a procedure reported in the literature.[1a]

A 1 L three-necked round-bottomed flask, equipped with a reflux condenser and stirring bar was dried under vacuum using a heat gun set to 550 °C, followed by three cycles of vacuum/argon. The flask was charged with 4-(trifluoromethyl)benzaldehyde (10.0 mL, 75.2 mmol, 1.00 equiv.) and THF (245 mL). The solution was cooled to −78 °C and $TiCl_4$ (10.0 mL, 91.5 mmol, 1.25 equiv.) was added dropwise. The reaction was allowed to warm to 25 °C and was stirred for 10 min. Zn dust (12.0 g, 183 mmol, 2.50 equiv.) was added portion-wise over 2 min. The reaction was refluxed at 70 °C for 16 h and then allowed to cool to 25 °C. Water (320 mL) was added, followed by an aqueous solution of HCl (73 mL, 1.0 M). The reaction was stirred for 5 min followed by separation of the layers. The aqueous layer was extracted with MTBE (2 × 500 mL). The combined organic layers were washed with brine and dried over $MgSO_4$. The solution was partially concentrated (approx. 200 mL), loaded onto a column and the material was purified *via* column chromatography over silica gel (hexanes). The fractions containing the product were concentrated to dryness and the solid material was dissolved in DMF (400 mL). The resulting solution was complemented with water (300 mL), where upon an off-white solid precipitated. The solid was filtered with a D3 fritted filter and washed with water (300 mL). The remainings were dissolved in CH_2Cl_2 (400 mL) and washed with water (2 × 200 mL). The organic layer was dried over $MgSO_4$, and concentrated under reduced pressure to afford $^{4\text{-CF3}}$stb as a white solid (7.28 g, 63%).

Spectroscopic data match those reported in the literature.[1a]

***R*$_f$:** 0.55 (hexanes)

^{1}H NMR (400 MHz, $CDCl_3$) δ 7.63 (s, 8H), 7.20 (s, 2H).

^{13}C NMR (101 MHz, $CDCl_3$) δ 140.2, 130.1 (q, J = 32.6 Hz), 129.7, 127.0, 125.9 (q, J = 3.8 Hz), 128.4 – 120.1 (m).

^{19}F NMR (282 MHz, $CDCl_3$) δ −62.57.

Ni($^{4\text{-}CF3}$stb)$_3$:

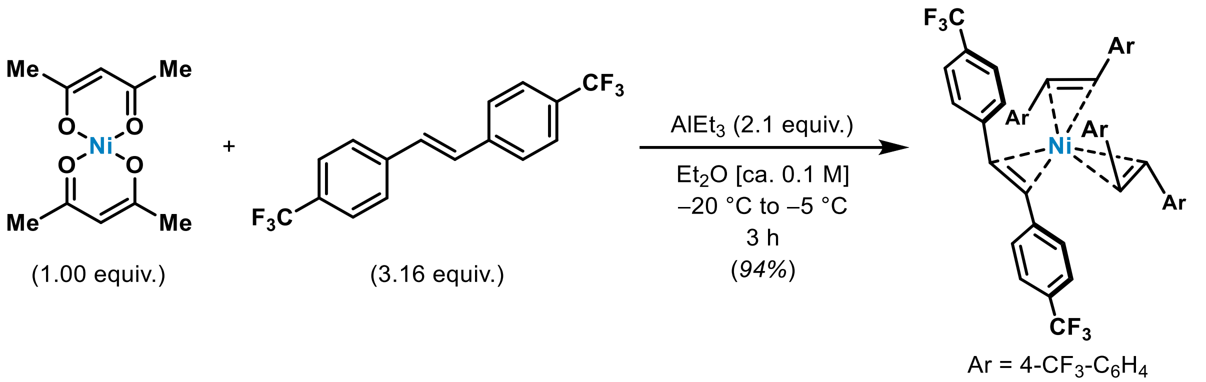

Ni($^{4\text{-}CF3}$stb)$_3$ was prepared using a slightly modified procedure, from the one reported.[1]

A 50 mL Schlenk flask equipped with a stirring bar, was dried under vacuum using a heat gun set to 550 °C, followed by three cycles of vacuum/argon. The flask was charged with anhydrous Ni(acac)$_2$ (0.50 g, 1.9 mmol, 1.0 equiv.) *via* argon trousers, followed by addition of $^{4\text{-}CF3}$stb (1.93 g, 6.12 mmol, 3.16 equiv.). One cycle of vacuum/argon was performed. Anhydrous Et$_2$O (20 mL) was added and the solution was cooled to −20 °C using a cryostat. A solution of AlEt$_3$ (1.0 M in hexanes, 4.1 mL, 2.1 equiv.) was added dropwise over 2 minutes to the reaction vessel. The reaction was allowed to warm up to −5 °C and stirred at that temperature for 3 hours. The suspension was cooled to −35 °C, stirred for 5 min at that temperature and then transferred (by using PE tubing) to a jacketed argon frit cooled to −35 °C. The suspension was filtered, leaving the product on the frit. The solid was washed with Et$_2$O (5 × 2 mL). The argon frit was transferred onto another Schlenk tube held under argon and dried under high vacuum, to afford the desired product as a red solid (1.83 g, 94%).

Preparation of Starting Materials

4,4,5,5-Tetraethyl-2-(thiophen-3-yl)-1,3,2-dioxaborolane (3-thienyl-BEpin, SM1):

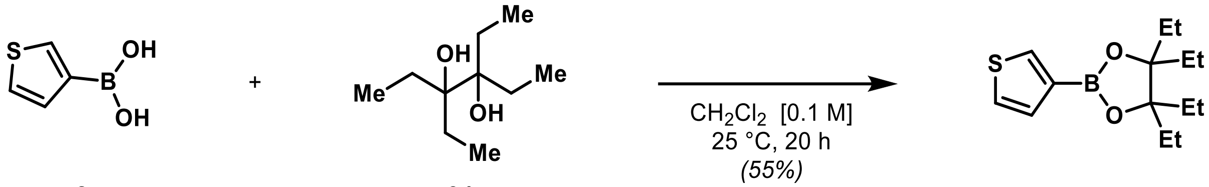

4,4,5,5-Tetraethyl-2-(thiophen-3-yl)-1,3,2-dioxaborolane was prepared based on a procedure reported in the literature.[2]

A 50 mL round-bottomed flask equipped with a stirring bar was dried under vacuum using a heat gun set to 550 °C, followed by three cycles of vacuum/argon. The flask was charged with 3-thienylboronic acid (256 mg, 2.00 mmol, 1.00 equiv.) and 3,4-diethylhexane-3,4-diol (348 mg, 2.00 mmol, 1.00 equiv.). One cycle of vacuum/argon was performed, and dry CH_2Cl_2 (20 mL) was added. The reaction was vigorously stirred at 25 °C. After 20 h the reaction mixture was concentrated under reduced pressure and filtered over a plug of silica gel using 50:50 pentane:MTBE as eluent to afford the title compound as a colorless oil (293 mg, 55%).

R

R_f: 0.99 (50:50 pentane:MTBE)

^{1}H NMR (400 MHz, $CDCl_3$) δ 7.92 (dd, J = 2.7, 1.0 Hz, 1H), 7.42 (dd, J = 4.8, 1.0 Hz, 1H), 7.33 (dd, J = 4.8, 2.7 Hz, 1H), 1.75 (qq, J = 14.5, 7.0 Hz, 8H), 0.96 (t, J = 7.5 Hz, 12H).

^{13}C NMR (101 MHz, $CDCl_3$) δ 136.4, 132.3, 125.3, 88.8, 26.5, 9.0.

^{11}B NMR (128 MHz, $CDCl_3$) δ 28.81.

HRMS (EI) m/z: [M] calc’d for $C_{14}H_{23}O_2SB$: 266.1506, found: 266.1507

FTIR (ATR): $\tilde{\nu}$ [cm^{-1}] = 2973, 2943, 2883, 1520, 1415, 1383, 1370, 1310, 1289, 1114, 1088, 926, 899, 855, 793, 679.

Note: In the ^{13}C NMR analysis, one signal could not be observed. However, this is in agreement with observations made for analogous compounds.[2]

(*S*)-4-(2-((*Tert*-butoxycarbonyl)amino)-3-methoxy-3-oxopropyl)phenyl 5-bromonicotinate (SM2):

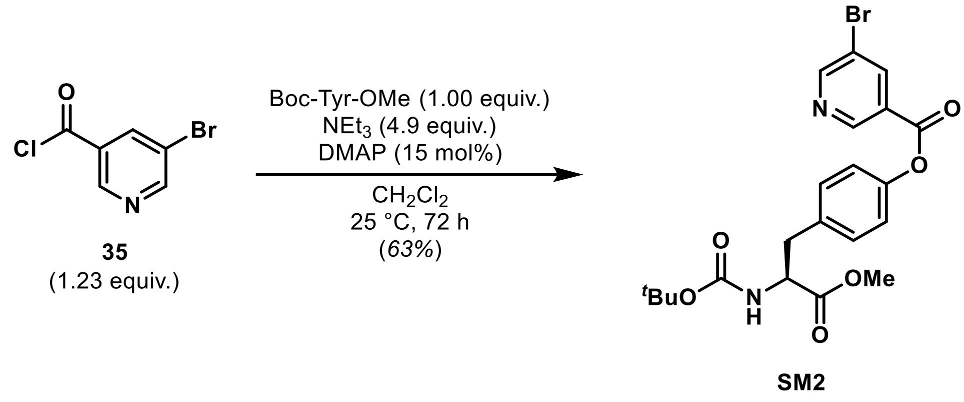

A 12-mL screwcap vial equipped with a stirring bar was charged with 5-bromonicotinoyl chloride (180 mg, 0.817 mmol, 1.23 equiv.), Boc-Tyr-OMe (196 mg, 0.662 mmol, 1.00 equiv.), catalytic amounts of DMAP (12.1 mg, 15 mol%), and CH_2Cl_2 (2.0 mL). NEt_3 (0.45 mL, 3.2 mmol, 4.9 equiv.) was added dropwise and the reaction mixture was stirred for 72 h at 25 °C. The reaction was quenched by the addition of water (3.0 mL). The phases were separated and the aqueous layer was extracted with CH_2Cl_2 (3 × 10 mL). The combined organic layers were washed with brine (10 mL), dried over $MgSO_4$ and concentrated under reduced pressure. Purification *via* column chromatography (silica gel, 50:50 pentane:MTBE) afforded the title compound as a beige solid (200 mg, 63%).

m.p.: 84.6–88.2 °C

***R_f*:** 0.52 (50:50 pentane:MTBE)

^{1}H NMR (400 MHz, $CDCl_3$) δ 9.28 (d, *J* = 1.8 Hz, 1H), 8.91 (d, *J* = 2.3 Hz, 1H), 8.57 (p, *J* = 2.3 Hz, 1H), 7.21 (d, *J* = 8.6 Hz, 2H), 7.18 – 7.12 (m, 2H), 5.03 (d, *J* = 8.3 Hz, 1H), 4.61 (q, *J* = 6.6 Hz, 1H), 3.73 (s, 3H), 3.21 – 3.03 (m, 2H), 1.43 (s, 9H).

^{13}C NMR (101 MHz, $CDCl_3$) δ 172.3, 162.7, 155.3, 155.2, 149.5, 149.4, 140.1, 134.6, 130.7, 127.1, 121.6, 120.9, 80.2, 54.5, 52.5, 38.0, 28.4.

HRMS (ESI) m/z: $[M+Na]^+$ calc'd for $C_{21}H_{23}N_2O_6BrNa$: 501.0632, found: 501.0631

FTIR (ATR): $\tilde{\nu}$ [cm^{-1}] = 3351, 3072, 2960, 2923, 2853, 1732, 1685, 1517, 1436, 1263, 1190, 1161, 1090, 1019, 754.

General Procedure (GP) for the Heteroaryl-Heteroaryl Suzuki-Miyaura Cross-Coupling

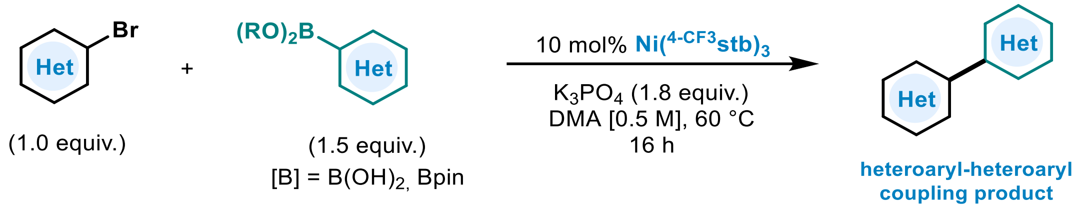

A 12-mL screwcap vial equipped with a stirring bar was dried under vacuum using a heat gun set to 550 °C, followed by three cycles of vacuum/argon. The vial was charged with Ni($^{4\text{-}CF_3}$stb)$_3$ (10 mol%), K_3PO_4 (1.8 equiv.) and with solid heteroaryl bromide (1.0 equiv.) and heteroaryl-B$(OH)_2$/heteroaryl-Bpin (1.5 equiv.), weighed under air. One cycle of vacuum/argon was performed. The reaction vessel was charged with liquid coupling partners using a Hamilton syringe through the rubber septum, while purging the liquid material with argon. DMA (0.5 M, corresponding to the heteroaryl bromide) was added using a syringe through the rubber septum, the cap of the vial was switched for a new one, and the reaction tube was placed into a pre-heated heating block held at 60 °C. The reaction was stirred for 16 h at that temperature and was then allowed to cool to 25 °C. The reaction was diluted with MTBE (1.5 mL), and quenched by the addition of a saturated aqueous solution of LiCl (10 mL). The phases were separated and the aqueous layer was extracted with MTBE (3–4 × 10 mL). The combined organic layers were dried over $MgSO_4$ and concentrated under reduced pressure. Purification was performed *via* column chromatography over silica gel or *via* preparative TLC (pTLC).

In some cases, residual amounts of the boron nucleophiles co-eluted with the products, therefore, additional acid/base or basic work-up was performed as follows and will be indicated when applied:

Acid/base work-up: The residual organic material was dissolved in MTBE (20 mL), and an aqueous solution of HCl (1 M) was added to set the pH to 1. The phases were separated and the aqueous layer was washed with MTBE (4 × 10 mL). An aqueous solution of NaOH (1 M) was added to set the pH to 14. MTBE was added and the phases were separated. The aqueous phase was then extracted with MTBE (3 × 10 mL). The combined organic layers were dried over $MgSO_4$ and dried under reduced pressure to give the pure desired product.

Basic work-up: The residual organic material was dissolved in MTBE (20 mL), an aqueous solution of NaOH (1 M) was added to set the pH to 14 and the phases were separated. The aqueous phase was then extracted with MTBE (3 × 10 mL). The combined organic layers were dried over $MgSO_4$ and dried under reduced pressure to give the pure desired product.

Note: Some of the reaction products exert certain solubility in water; hence, it is advised to carefully check the aqueous phase for residual organic material *via* TLC in every extraction procedure.

Reaction Optimization

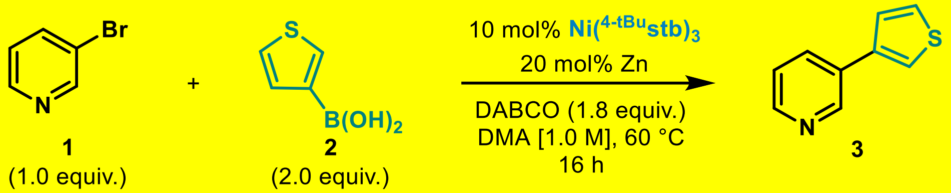

entry	deviations from above	yield[a] in % of **3**
1	none	8
2	Ni($^{4\text{-}CF3}$stb)$_3$	17
3	Ni($^{4\text{-}CF3}$stb)$_3$, no Zn	14
4	Ni($^{4\text{-}CF3}$stb)$_3$, no Zn, K_3PO_4, DMA [0.5 M]	88
5	Ni($^{4\text{-}CF3}$stb)$_3$, no Zn, K_3PO_4, DMA [0.5 M], 110 °C	90
6	Ni($^{4\text{-}CF3}$stb)$_3$, no Zn, K_3PO_4, DMA [0.5 M], 1.1 equiv. NaI	88
7	Ni($^{4\text{-}CF3}$stb)$_3$, no Zn, K_3PO_4, DMA [0.5 M], 1.5 equiv. **2**	93 (86)[b]
8	Ni($^{4\text{-}CF3}$stb)$_3$, no Zn, K_3PO_4, DMA [0.5 M], 1.5 equiv. 3-thienyl-Bpin	86
9	Ni($^{4\text{-}CF3}$stb)$_3$, no Zn, K_3PO_4, DMA [0.5 M], 1.5 equiv. 3-thienyl-BEpin	44
10	Ni($^{4\text{-}CF3}$stb)$_3$, no Zn, K_3PO_4, DMA [0.5 M], 1.5 equiv. 3-thienyl-BF_3K	n.d.
11	5 mol% Ni($^{4\text{-}CF3}$stb)$_3$, no Zn, K_3PO_4, DMA [0.5 M], 1.5 equiv. **2**	59
12	Ni($^{4\text{-}tBu}$stb)$_3$, no Zn, K_3PO_4, DMA [0.5 M], 1.5 equiv. **2**	83
13	$NiCl_2$•6H_2O, no Zn, K_3PO_4, DMA [0.5 M], 1.5 equiv. **2**	18
14	$NiCl_2$•6H_2O, 30 mol% $^{4\text{-}CF3}$stb, no Zn, K_3PO_4, DMA [0.5 M], 1.5 equiv. **2**	18
15	Ni(COD)$_2$, no Zn, K_3PO_4, DMA [0.5 M], 1.5 equiv. **2**	73
16	Ni(COD)(DQ), no Zn, K_3PO_4, DMA [0.5 M], 1.5 equiv. **2**	26
17	no [Ni], no Zn, K_3PO_4, DMA [0.5 M], 1.5 equiv. **2**	n.d.

[a] ^{1}H NMR yield as determined by using 1,3,5-trimethoxybenzene as internal standard.
[b] Isolated yield (0.3 mmol scale), $^{4\text{-}tBu}$stb = (*E*)-1,2-bis(4-(*tert*-butyl)phenyl)ethene, $^{4\text{-}CF3}$stb = (*E*)-1,2-bis(4-(trifluoromethyl)phenyl)ethene, COD = 1,5-cyclooctadiene, DQ = duroquinone

Note: Reactions were analyzed after aqueous work-up according to the **GP**. 1,3,5-Trimethoxybenzene was added to the crude material as IS and $CDCl_3$ was added. An aliquot was transferred into an NMR tube and the yields were determined based on the integration of the internal standard (at 6.05 ppm). It was found that integration of signals in the area of 7.52 – 7.25 ppm is less accurate, as integration usually appears too high due to signal overlap. Signals at 8.85 ppm, 8.50 ppm and 7.86 ppm were determined to be the most reliable and were integrated (as long as no overlap was detected). The yield presented was averaged based on these integrations.

^{1}H NMR (400 MHz, $CDCl_3$) δ 8.85 (s, 1H), 8.50 (s, 1H), 7.86 (d, *J* = 7.9 Hz, 1H), 7.50 (dd, *J* = 2.9, 1.4 Hz, 1H), 7.41 (dd, *J* = 5.0, 2.9 Hz, 1H), 7.36 (dd, *J* = 5.0, 1.4 Hz, 1H).

3-(Thiophen-3-yl)pyridine (3):

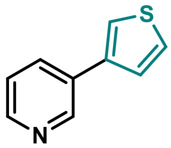

3-(Thiophen-3-yl)pyridine was prepared according to the **GP**, using Ni($^{4\text{-}CF_3}$stb)$_3$ (30.3 mg, 10 mol%), K_3PO_4 (115 mg, 0.543 mmol, 1.80 equiv.), 3-bromopyridine (29 µL, 0.30 mmol, 1.0 equiv.), and 3-thienylboronic acid (57.8 mg, 0.452 mmol, 1.50 equiv.) in DMA (0.6 mL) at 60 °C. Purification *via* pTLC (silica gel, 80:20 pentane:MTBE) afforded the title compound as an off-white solid (41.5 mg, 86%). Spectroscopic data matched those reported in the literature.[3]

m.p.: 76.0 – 77.0 °C

***R_f*:** 0.16 (80:20 pentane:MTBE)

^{1}H NMR (400 MHz, $CDCl_3$) δ 8.88 (dd, *J* = 2.4, 0.9 Hz, 1H), 8.53 (dd, *J* = 4.8, 1.7 Hz, 1H), 7.87 (ddd, *J* = 7.9, 2.3, 1.6 Hz, 1H), 7.53 (dd, *J* = 2.9, 1.4 Hz, 1H), 7.45 (dd, *J* = 5.0, 2.9 Hz, 1H), 7.40 (dd, *J* = 5.0, 1.4 Hz, 1H), 7.33 (ddd, *J* = 8.0, 4.8, 0.9 Hz, 1H).

^{13}C NMR (101 MHz, $CDCl_3$) δ 148.3, 147.8, 138.9, 133.6, 131.6, 127.1, 126.0, 123.7, 121.5.

Methyl 5-(thiophen-3-yl)nicotinate (4):

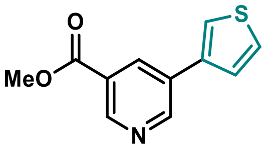

Methyl 5-(thiophen-3-yl)nicotinate was prepared according to the **GP**, using Ni($^{4\text{-}CF_3}$stb)$_3$ (30.3 mg, 10 mol%), K_3PO_4 (115 mg, 0.542 mmol, 1.81 equiv.), methyl 5-bromonicotinate (64.9 mg, 0.300 mmol, 1.00 equiv.), and 3-thienylboronic acid (57.7 mg, 0.451 mmol, 1.50 equiv.) in DMA (0.6 mL) at 60 °C. Purification *via* pTLC (silica gel, 70:30 pentane:MTBE) afforded the title compound as an off-white solid (52.8 mg, 80%).

m.p.: 100.3 – 103.3 °C

***R_f*:** 0.31 (70:30 pentane:MTBE)

^{1}H NMR (400 MHz, $CDCl_3$) δ 9.13 (d, *J* = 2.0 Hz, 1H), 9.03 (d, *J* = 2.3 Hz, 1H), 8.47 (t, *J* = 2.2 Hz, 1H), 7.62 (dd, *J* = 2.9, 1.4 Hz, 1H), 7.48 (dd, *J* = 5.0, 2.9 Hz, 1H), 7.45 (dd, *J* = 5.1, 1.4 Hz, 1H), 3.99 (s, 3H).

^{13}C NMR (101 MHz, $CDCl_3$) δ 166.0, 151.3, 149.2, 137.8, 134.5, 131.5, 127.5, 126.2, 126.0, 122.4, 52.7.

HRMS (EI) m/z: [M] calc'd for $C_{11}H_9NO_2S$: 219.0349, found: 219.0351

FTIR (ATR): $\tilde{\nu}$ [cm^{-1}] = 3059, 2954, 2922, 2853, 1717, 1426, 1295, 1247, 1216, 1120, 842, 822, 761, 724, 694, 637.

2-Methyl-4-(thiophen-3-yl)pyridine (5):

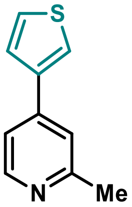

2-Methyl-4-(thiophen-3-yl)pyridine was prepared according to the **GP**, using Ni($^{4\text{-}CF3}$stb)$_3$ (30.2 mg, 10 mol%), K_3PO_4 (115 mg, 0.540 mmol, 1.80 equiv.), 4-bromo-2-methylpyridine (51.7 mg, 0.300 mmol, 1.00 equiv.), and 3-thienylboronic acid (57.8 mg, 0.452 mmol, 1.50 equiv.) in DMA (0.6 mL) at 60 °C. Purification *via* pTLC (silica gel, 99:1 CH_2Cl_2:MeOH), followed by a basic work-up afforded the title compound as a yellow oil (22.8 mg, 43%).

***Rf*:** 0.18 (99:1 CH_2Cl_2:MeOH)

^{1}H NMR (400 MHz, $CDCl_3$) δ 8.50 (dd, *J* = 5.3, 0.8 Hz, 1H), 7.63 (dd, *J* = 2.4, 1.9 Hz, 1H), 7.45 – 7.40 (m, 2H), 7.35 (dd, *J* = 1.8, 0.8 Hz, 1H), 7.32 – 7.24 (m, 1H), 2.60 (s, 3H).

^{13}C NMR (101 MHz, $CDCl_3$) δ 159.1, 149.8, 143.1, 139.9, 127.1, 125.9, 123.0, 120.5, 118.2, 24.7.

HRMS (EI) m/z: [M] calc'd for $C_{10}H_9NS$: 175.0450, found: 175.0448

FTIR (ATR): $\tilde{\nu}$ [cm^{-1}] = 3369, 3105, 2923, 2853, 1065, 1558, 1524, 1481, 1354, 1424, 844, 783, 682, 648, 614, 557, 450.

2-(Thiophen-3-yl)-5-(trifluoromethyl)pyridine (6):

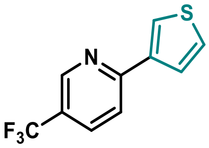

2-(Thiophen-3-yl)-5-(trifluoromethyl)pyridine was prepared according to the **GP**, using Ni($^{4\text{-}CF3}$stb)$_3$ (30.3 mg, 10 mol%), K_3PO_4 (115 mg, 0.539 mmol, 1.80 equiv.), 2-bromo-5-(trifluoromethyl)pyridine (67.8 mg, 0.300 mmol, 1.00 equiv.), and 3-thienylboronic acid (57.7 mg, 0.451 mmol, 1.50 equiv.) in DMA (0.6 mL) at 60 °C. Purification *via* pTLC (silica gel, 95:5 pentane:MTBE) afforded the title compound as a colorless oil (51.4 mg, 75%).

Spectroscopic data matched those reported in the literature.[4]

R_f: 0.50 (95:5 pentane:MTBE)

^{1}H NMR (400 MHz, $CDCl_3$) δ 8.87 (dd, *J* = 1.5, 0.9 Hz, 1H), 8.02 (dd, *J* = 3.0, 1.3 Hz, 1H), 7.96 – 7.90 (m, 1H), 7.74 – 7.66 (m, 2H), 7.43 (dd, *J* = 5.1, 3.0 Hz, 1H).

^{13}C NMR (101 MHz, $CDCl_3$) δ 156.7, 146.8 (q, *J* = 4.2 Hz), 141.0, 134.0 (q, *J* = 3.6 Hz), 127.0, 130.9 – 122.4 (m), 126.3, 125.7, 125.1 – 124.0 (m), 119.7.

^{19}F NMR (282 MHz, $CDCl_3$) δ −62.26.

6-(Thiophen-3-yl)picolinonitrile (7):

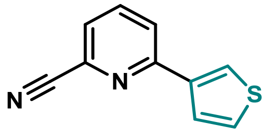

6-(Thiophen-3-yl)picolinonitrile was prepared according to the **GP**, using Ni($^{4\text{-}CF3}$stb)$_3$ (30.3 mg, 10 mol%), K_3PO_4 (115 mg, 0.542 mmol, 1.80 equiv.), 6-bromopicolinonitrile (36.9 mg, 0.300 mmol, 1.00 equiv.), and 3-thienylboronic acid (57.7 mg, 0.451 mmol, 1.50 equiv.) in DMA (0.6 mL) at 60 °C. Purification *via* pTLC (silica gel, 80:20:4 pentane:CH_2Cl_2:EtOAc) afforded the title compound as an off-white solid (18.1 mg, 32%).

m.p.: 76.0 – 86.3 °C

***R$_f$*:** 0.38 (80:20:4 pentane:CH_2Cl_2:EtOAc)

^{1}H NMR (400 MHz, $CDCl_3$) 8.00 (dd, *J* = 3.0, 1.3 Hz, 1H), 7.86 – 7.79 (m, 2H), 7.68 (dd, *J* = 5.1, 1.3 Hz, 1H), 7.55 (dd, *J* = 6.6, 2.0 Hz, 1H), 7.43 (dd, *J* = 5.1, 3.0 Hz, 1H).

^{13}C NMR (101 MHz, $CDCl_3$) δ 155.1, 140.3, 137.8, 133.9, 127.1, 126.4, 126.2, 125.6, 123.4, 117.5.

HRMS (EI) m/z: [M] calc'd for $C_{10}H_6N_2S$: 186.0246, found: 186.0247

FTIR (ATR): $\tilde{\nu}$ [cm^{-1}] = 3106, 2925, 2851, 2237, 1586, 1559, 1526, 1454, 1428, 1200, 988, 874, 839, 820, 784, 673.

5-(Thiophen-3-yl)pyrimidine (8):

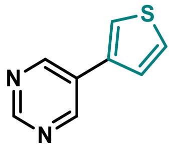

5-(Thiophen-3-yl)pyrimidine was prepared according to the **GP**, using Ni($^{4\text{-}CF3}$stb)$_3$ (30.3 mg, 10 mol%), K_3PO_4 (115 mg, 0.543 mmol, 1.80 equiv.), 5-bromopyrimidine (47.9 mg, 0.301 mmol, 1.00 equiv.), and 3-thienylboronic acid (57.8 mg, 0.452 mmol, 1.50 equiv.) in DMA (0.6 mL) at 60 °C. Purification *via* pTLC (silica gel, 70:30 pentane:MTBE) afforded the title compound as a white solid (36.1 mg, 74%).
Spectroscopic data matched those reported in the literature.[3]

m.p.: 102.3 – 103.3 °C

***R$_f$*:** 0.22 (70:30 pentane:MTBE)

^{1}H NMR (400 MHz, $CDCl_3$) δ 9.14 (s, 1H), 8.96 (s, 2H), 7.61 (dd, *J* = 2.9, 1.4 Hz, 1H), 7.51 (dd, *J* = 5.1, 2.9 Hz, 1H), 7.41 (dd, *J* = 5.0, 1.4 Hz, 1H).

^{13}C NMR (101 MHz, $CDCl_3$) δ 157.3, 154.3, 135.3, 129.7, 128.0, 125.6, 122.7.

2-(Thiophen-3-yl)pyrazine (9):

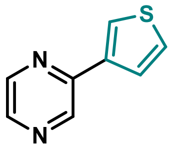

2-(Thiophen-3-yl)pyrazine was prepared according to the **GP**, using Ni($^{4\text{-CF3}}$stb)$_3$ (30.3 mg, 10 mol%), K_3PO_4 (115 mg, 0.544 mmol, 1.82 equiv.), 2-bromopyrazine (27 µL, 0.30 mmol, 1.0 equiv.), and 3-thienylboronic acid (57.8 mg, 0.452 mmol, 1.51 equiv.) in DMA (0.6 mL) at 60 °C. Purification *via* pTLC (silica gel, 70:30 pentane:MTBE) afforded the title compound as a beige solid (36.3 mg, 75%).

Spectroscopic data matched those reported in the literature.[5]

m.p.: 81.0 – 83.5 °C

***R_f*:** 0.30 (70:30 pentane:MTBE)

^{1}H NMR (400 MHz, CDCl$_3$) δ 8.93 (d, *J* = 1.5 Hz, 1H), 8.57 (dd, *J* = 2.5, 1.5 Hz, 1H), 8.44 (d, *J* = 2.5 Hz, 1H), 8.00 (dd, *J* = 3.0, 1.3 Hz, 1H), 7.70 (dd, *J* = 5.1, 1.3 Hz, 1H), 7.46 (dd, *J* = 5.1, 3.0 Hz, 1H).

^{13}C NMR (101 MHz, CDCl$_3$) δ 149.3, 144.4, 142.7, 142.1, 139.1, 127.2, 125.9, 125.0.

4-(Thiophen-3-yl)isoquinoline (10):

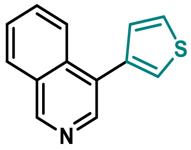

4-(Thiophen-3-yl)isoquinoline was prepared according to the **GP**, using Ni($^{4\text{-CF3}}$stb)$_3$ (30.3 mg, 10 mol%), K_3PO_4 (116 mg, 0.545 mmol, 1.81 equiv.), 4-bromoisoquinoline (62.5 mg, 0.300 mmol, 1.00 equiv.), and 3-thienylboronic acid (57.6 mg, 0.450 mmol, 1.50 equiv.) in DMA (0.6 mL) at 60 °C. Purification *via* pTLC (silica gel, 70:30 pentane:MTBE) afforded the title compound as a colorless oil (57.2 mg, 90%).

Spectroscopic data matched those reported in the literature.[6]

***R_f*:** 0.23 (70:30 pentane:MTBE)

^{1}H NMR (400 MHz, CDCl$_3$) δ 9.26 – 9.21 (m, 1H), 8.55 (s, 1H), 8.08 – 8.00 (m, 2H), 7.70 (ddd, *J* = 8.4, 6.8, 1.4 Hz, 1H), 7.64 (ddd, *J* = 8.1, 6.8, 1.2 Hz, 1H), 7.52 (dd, *J* = 4.9, 3.0 Hz, 1H), 7.47 (dd, *J* = 3.0, 1.3 Hz, 1H), 7.34 (dd, *J* = 4.9, 1.3 Hz, 1H).

^{13}C NMR (101 MHz, CDCl$_3$) δ 152.1, 142.9, 137.5, 134.5, 130.8, 129.4, 128.6, 128.4, 128.1, 127.4, 126.2, 124.8, 124.5.

4-(Thiophen-3-yl)quinoline (11):

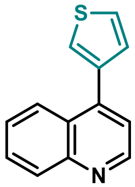

4-(Thiophen-3-yl)quinoline was prepared according to the **GP**, using Ni($^{4\text{-}CF_3}$stb)$_3$ (30.3 mg, 10 mol%), K_3PO_4 (115 mg, 0.542 mmol, 1.81 equiv.), 4-bromoquinoline (62.4 mg, 0.300 mmol, 1.00 equiv.), and 3-thienylboronic acid (57.7 mg, 0.451 mmol, 1.50 equiv.) in DMA (0.6 mL) at 60 °C. Purification *via* pTLC (silica gel, 80:20 pentane:EtOAc) afforded the title compound as a yellowish liquid (42.0 mg, 66%). Spectroscopic data matched those reported in the literature.[7]

R R_f**:** 0.25 (80:20 pentane:EtOAc)

^{1}H NMR (400 MHz, $CDCl_3$) δ 8.92 (d, *J* = 4.5 Hz, 1H), 8.17 (ddd, *J* = 8.5, 1.4, 0.6 Hz, 1H), 8.08 (ddd, *J* = 8.5, 1.5, 0.6 Hz, 1H), 7.73 (ddd, *J* = 8.4, 6.8, 1.4 Hz, 1H), 7.57 – 7.46 (m, 3H), 7.38 (d, *J* = 4.4 Hz, 1H), 7.34 (dd, *J* = 4.5, 1.8 Hz, 1H).

^{13}C NMR (75 MHz, $CDCl_3$) δ 150.1, 148.8, 143.3, 138.6, 130.0, 129.5, 129.0, 126.89, 126.84, 126.4, 125.8, 125.1, 121.2.

3-(Thiophen-3-yl)-1,5-naphthyridine (12):

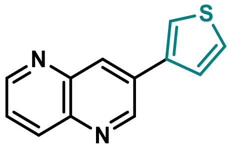

3-(Thiophen-3-yl)-1,5-naphthyridine was prepared according to the **GP**, using Ni($^{4\text{-}CF_3}$stb)$_3$ (30.2 mg, 10 mol%), K_3PO_4 (115 mg, 0.542 mmol, 1.81 equiv.), 3-bromo-1,5-naphthyridine (62.7 mg, 0.300 mmol, 1.00 equiv.), and 3-thienylboronic acid (57.7 mg, 0.451 mmol, 1.50 equiv.) in DMA (0.6 mL) at 60 °C. Purification *via* pTLC (silica gel, 20:80 pentane:MTBE) afforded the title compound as a white solid (59.9 mg, 94%).

m.p.: 129.0 – 133.4 °C

R_f**:** 0.20 (20:80 pentane:MTBE)

^{1}H NMR (400 MHz, $CDCl_3$) δ 9.27 (d, *J* = 2.2 Hz, 1H), 8.98 (dd, *J* = 4.2, 1.7 Hz, 1H), 8.52 (dd, *J* = 2.2, 0.9 Hz, 1H), 8.39 (ddd, *J* = 8.5, 1.7, 0.9 Hz, 1H), 7.74 (dd, *J* = 2.9, 1.4 Hz, 1H), 7.61 (dd, *J* = 8.5, 4.2 Hz, 1H), 7.56 (dd, *J* = 5.0, 1.4 Hz, 1H), 7.51 (dd, *J* = 5.0, 2.9 Hz, 1H).

^{13}C NMR (101 MHz, $CDCl_3$) δ 151.8, 150.3, 144.1, 142.8, 138.2, 137.2, 133.0, 132.2, 127.6, 126.2, 124.1, 122.8.

HRMS (EI) m/z: [M] calc'd for $C_{12}H_8N_2S$: 212.0403, found: 212.0404

FTIR (ATR): $\tilde{\nu}$ [cm^{-1}] = 3070, 1605, 1536, 1492, 1462, 1454, 1311, 1193, 1116, 909, 850, 819, 796, 766, 626, 612.

6,7-Dimethoxy-4-(thiophen-3-yl)quinazoline (13):

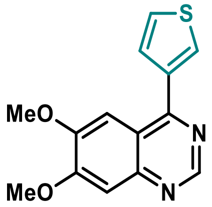

6,7-Dimethoxy-4-(thiophen-3-yl)quinazoline was prepared according to the **GP**, using Ni($^{4\text{-}CF3}$stb)$_3$ (30.3 mg, 10 mol%), K_3PO_4 (114 mg, 0.537 mmol, 1.79 equiv.), 4-chloro-6,7-dimethoxyquinazoline (67.4 mg, 0.300 mmol, 1.00 equiv.), and 3-thienylboronic acid (57.7 mg, 0.451 mmol, 1.50 equiv.) in DMA (0.6 mL) at 100 °C. Purification *via* pTLC (silica gel, MTBE) afforded the title compound as a beige solid (25.9 mg, 32%).

m.p.: 159.5 – 161.6 °C

***R$_f$*:** 0.34 (MTBE)

^{1}H NMR (400 MHz, CDCl$_3$) δ 9.14 (s, 1H), 7.90 (dd, *J* = 2.9, 1.3 Hz, 1H), 7.62 (dd, *J* = 5.0, 1.3 Hz, 1H), 7.53 (dd, *J* = 5.0, 3.0 Hz, 1H), 7.50 (s, 1H), 7.36 (s, 1H), 4.07 (s, 3H), 3.97 (s, 3H).

^{13}C NMR (101 MHz, CDCl$_3$) δ 160.0, 155.9, 153.8, 150.7, 149.4, 139.4, 128.7, 127.7, 126.7, 118.8, 107.2, 103.8, 56.6, 56.3.

HRMS (ESI) m/z: $[M+H]^+$ calc'd for $C_{14}H_{13}N_2O_2S$: 273.0692, found: 273.0693

FTIR (ATR): $\tilde{\nu}$ [cm^{-1}] = 3093, 2919, 2829, 1500, 1428, 1355, 1231, 1214, 1179, 1136, 1012, 851, 773, 762, 673.

(*S*)-4-(2-((*tert*-Butoxycarbonyl)amino)-3-methoxy-3-oxopropyl)phenyl 5-(thiophen-3-yl)nicotinate (14):

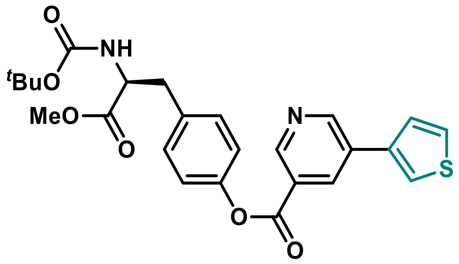

(*S*)-4-(2-((*tert*-Butoxycarbonyl)amino)-3-methoxy-3-oxopropyl)phenyl 5-(thiophen-3-yl)nicotinate was prepared according to the **GP**, using Ni($^{4\text{-}CF3}$stb)$_3$ (30.3 mg, 10 mol%), K_3PO_4 (115mg, 0.539 mmol, 1.80 equiv.), **SM2** (144 mg, 0.300 mmol, 1.00 equiv.), and 3-thienylboronic acid (57.8 mg, 0.452 mmol, 1.51 equiv.) in DMA (0.6 mL) at 60 °C. Purification *via* pTLC (silica gel, 97:3 CH_2Cl_2:MeOH) afforded the title compound as a colorless oil (55.2 mg, 38%).

***R$_f$*:** 0.38 (97:3 CH_2Cl_2:MeOH)

^{1}H NMR (400 MHz, CDCl$_3$) δ 9.28 (t, *J* = 1.9 Hz, 1H), 9.09 (t, *J* = 2.1 Hz, 1H), 8.59 (dt, *J* = 4.3, 2.0 Hz, 1H), 7.65 (dt, *J* = 2.8, 1.4 Hz, 1H), 7.53 – 7.43 (m, 2H), 7.24 – 7.16 (m, 4H), 5.07 (d, *J* = 8.4 Hz, 1H), 4.61 (q, *J* = 6.7 Hz, 1H), 3.73 (s, 3H), 3.12 (qd, *J* = 13.9, 6.0 Hz, 2H), 1.43 (s, 9H).

^{13}C NMR (101 MHz, CDCl$_3$) δ 172.3, 163.9, 155.2, 151.7, 149.6, 149.5, 137.6, 134.9, 134.4, 131.7, 130.6, 127.6, 125.9, 125.7, 122.6, 121.7, 80.2, 54.5, 52.4, 37.9, 28.4.

HRMS (ESI) m/z: $[M+Na]^+$ calc'd for $C_{25}H_{26}O_6N_2SNa$: 505.1404, found: 505.1403

FTIR (ATR): $\tilde{\nu}$ [cm^{-1}] = 3378, 3105, 2976, 2928, 2855, 1740, 1711, 1507, 1365, 1292, 1232, 1196, 1166, 1052, 1020, 756.

3-(Furan-3-yl)pyridine (15):

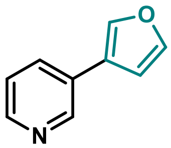

3-(Furan-3-yl)pyridine was prepared according to the **GP**, using Ni($^{4\text{-}CF3}$stb)$_3$ (30.2 mg, 10 mol%), K_3PO_4 (116 mg, 0.544 mmol, 1.81 equiv.), 3-bromopyridine (29 µL, 0.30 mmol, 1.0 equiv.), and 3-furanylboronic acid (50.5 mg, 0.451 mmol, 1.50 equiv.) in DMA (0.6 mL) at 60 °C. Purification *via* pTLC (silica gel, 70:30 pentane:MTBE) afforded the title compound as a colorless oil (39.5 mg, 90%).

Spectroscopic data matched those reported in the literature.[3]

***R$_f$*:** 0.26 (70:30 pentane:MTBE)

^{1}H NMR (400 MHz, CDCl$_3$) δ 8.77 (dt, J = 2.2, 1.1 Hz, 1H), 8.50 (dt, J = 4.9, 1.6 Hz, 1H), 7.82 – 7.71 (m, 2H), 7.52 (q, J = 1.7 Hz, 1H), 7.30 (dddd, J = 7.7, 4.8, 2.0, 0.9 Hz, 1H), 6.71 (td, J = 1.8, 0.9 Hz, 1H).
^{13}C NMR (101 MHz, CDCl$_3$) δ 148.3, 147.4, 144.3, 139.1, 133.2, 128.5, 123.8, 123.4, 108.6.

3-(Benzo[*b*]thiophen-3-yl)pyridine (16):

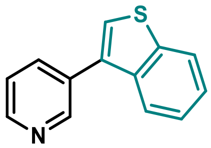

3-(Benzo[*b*]thiophen-3-yl)pyridine was prepared according to the **GP**, using Ni($^{4\text{-}CF3}$stb)$_3$ (30.2 mg, 10 mol%), K_3PO_4 (115 mg, 0.539 mmol, 1.79 equiv.), 3-bromopyridine (29 µL, 0.30 mmol, 1.0 equiv.), and benzo[*b*]thien-2-ylboronic acid (80.4 mg, 0.452 mmol, 1.50 equiv.) in DMA (0.6 mL) at 60 °C. Purification *via* pTLC (silica gel, 70:30 pentane:MTBE) afforded the title compound as a colorless oil (48.1 mg, 76%). Spectroscopic data matched those reported in the literature.[8]
***R$_f$*:** 0.28 (70:30 pentane:MTBE)
^{1}H NMR (400 MHz, CDCl$_3$) δ 8.86 (dd, J = 2.3, 0.9 Hz, 1H), 8.66 (dd, J = 4.8, 1.7 Hz, 1H), 7.97 – 7.93 (m, 1H), 7.90 (ddd, J = 7.8, 2.3, 1.7 Hz, 1H), 7.87 – 7.83 (m, 1H), 7.48 (s, 1H), 7.46 – 7.40 (m, 3H).
^{13}C NMR (101 MHz, CDCl$_3$) δ 149.7, 148.9, 140.8, 137.7, 136.0, 134.5, 132.0, 124.9, 124.83, 124.82, 123.7, 123.2, 122.5.

3-(Benzofuran-2-yl)pyridine (17):

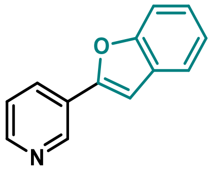

3-(Benzofuran-2-yl)pyridine was prepared according to the **GP**, using Ni($^{4\text{-}CF3}$stb)$_3$ (30.2 mg, 10 mol%), K_3PO_4 (114 mg, 0.539 mmol, 1.80 equiv.), 3-bromopyridine (29 µL, 0.30 mmol, 1.0 equiv.), and benzofuran-2-boronic acid pinacol ester (110 mg, 0.452 mmol, 1.50 equiv.) in DMA (0.6 mL) at 60 °C. Purification *via* pTLC (silica gel, 99:1 CH_2Cl_2:MeOH), followed by an acid/base work-up afforded the title compound as a white solid (47.0 mg, 80%).
Spectroscopic data matched those reported in the literature.[3]
m.p.: 77.4 – 79.8 °C
***R$_f$*:** 0.40 (99:1 CH_2Cl_2:MeOH)

^{1}H NMR (400 MHz, $CDCl_3$) δ 9.12 (dd, J = 2.3, 0.9 Hz, 1H), 8.58 (dd, J = 4.8, 1.6 Hz, 1H), 8.13 (ddd, J = 8.0, 2.3, 1.7 Hz, 1H), 7.62 (ddd, J = 7.7, 1.4, 0.7 Hz, 1H), 7.55 (dq, J = 8.2, 0.9 Hz, 1H), 7.38 (ddd, J = 8.0, 4.9, 0.9 Hz, 1H), 7.36 – 7.30 (m, 1H), 7.29 – 7.24 (m, 1H), 7.13 (d, J = 1.0 Hz, 1H).
^{13}C NMR (101 MHz, $CDCl_3$) δ 155.2, 153.1, 149.5, 146.6, 132.0, 128.9, 126.8, 125.1, 123.8, 123.4, 121.4, 111.5, 102.9.

tert-Butyl 4-(4-(pyridin-3-yl)-1*H*-pyrazol-1-yl)piperidine-1-carboxylate (18):

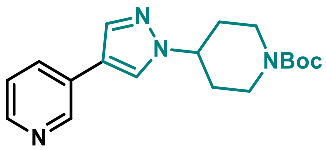

tert-Butyl 4-(4-(pyridin-3-yl)-1*H*-pyrazol-1-yl)piperidine-1-carboxylate was prepared according to the **GP**, using Ni($^{4\text{-}CF3}$stb)$_3$ (30.3 mg, 10 mol%), K_3PO_4 (115 mg, 0.542 mmol, 1.80 equiv.), 3-bromopyridine (29 µL, 0.30 mmol, 1.0 equiv.), and *tert*-butyl 4-(4-(4,4,5,5-tetramethyl-1,3,2-dioxaborolan-2-yl)-1*H*-pyrazol-1-yl)piperidine-1-carboxylate (170 mg, 0.452 mmol, 1.50 equiv.) in DMA (0.6 mL) at 60 °C. Purification *via* pTLC (silica gel, 95:5 CH_2Cl_2:MeOH) followed by a basic work-up afforded the title compound as a white solid (58.1 mg, 59%).
m.p.: 140.8 – 141.8 °C
***R_f*:** 0.29 (95:5 CH_2Cl_2:MeOH)
^{1}H NMR (400 MHz, $CDCl_3$) δ 8.76 – 8.70 (m, 1H), 8.44 (dd, J = 4.8, 1.6 Hz, 1H), 7.79 (d, J = 0.8 Hz, 1H), 7.74 – 7.69 (m, 2H), 7.30 – 7.22 (m, 1H), 4.35 – 4.23 (m, 3H), 2.91 (d, J = 12.9 Hz, 2H), 2.18 – 2.11 (m, 2H), 1.94 (qd, J = 12.2, 4.5 Hz, 2H), 1.46 (s, 9H).
^{13}C NMR (101 MHz, $CDCl_3$) δ 154.6, 147.7, 147.0, 136.6, 132.7, 128.7, 123.9, 123.8, 119.4, 80.1, 59.7, 42.9, 32.5, 28.5.
HRMS (ESI) m/z: $[M+H]^+$ calc'd for $C_{18}H_{25}N_4O_2$: 329.1972, found: 329.1970
FTIR (ATR): $\tilde{\nu}$ [cm^{-1}] = 3474, 3093, 2974, 2933, 2862, 1687, 1421, 1366, 1277, 1236, 1167, 1127, 981, 954, 710.

5-(Pyridin-3-yl)-1*H*-indole (19):

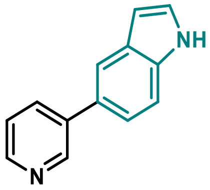

5-(Pyridin-3-yl)-1*H*-indole was prepared according to the **GP**, using Ni($^{4\text{-CF3}}$stb)$_3$ (30.3 mg, 10 mol%), K_3PO_4 (116 mg, 0.546 mmol, 1.81 equiv.), 3-bromopyridine (29 µL, 0.30 mmol, 1.0 equiv.), and 5-indolylboronic acid (72.7 mg, 0.452 mmol, 1.50 equiv.) in DMA (0.6 mL) at 60 °C. Purification *via* pTLC (silica gel, 50:50 pentane:MTBE) followed by a basic work-up afforded the title compound as a beige solid (34.0 mg, 58%).

Spectroscopic data matched those reported in the literature.[9]

m.p.: 160.6 – 161.9 °C

R**$_f$:** 0.20 (50:50 pentane:MTBE)

^{1}H NMR (400 MHz, CDCl$_3$) δ 8.92 (dd, *J* = 2.4, 0.9 Hz, 1H), 8.56 (dd, *J* = 4.8, 1.6 Hz, 1H), 8.41 (brs, 1H), 7.93 (ddd, *J* = 7.9, 2.4, 1.7 Hz, 1H), 7.86 (dt, *J* = 1.7, 0.8 Hz, 1H), 7.50 (dt, *J* = 8.5, 0.9 Hz, 1H), 7.43 (dd, *J* = 8.5, 1.7 Hz, 1H), 7.36 (ddd, *J* = 7.9, 4.8, 0.9 Hz, 1H), 7.28 (dd, *J* = 3.2, 2.4 Hz, 1H), 6.64 (ddd, *J* = 3.1, 2.0, 0.9 Hz, 1H).

^{13}C NMR (101 MHz, CDCl$_3$) δ 148.7, 147.6, 138.1, 135.8, 134.7, 130.0, 128.7, 125.4, 123.6, 121.7, 119.6, 111.8, 103.2.

5-Methyl-4-(pyridin-3-yl)-1-(tetrahydro-2*H*-pyran-3-yl)-1*H*-indazole (20):

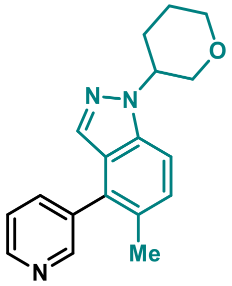

5-Methyl-4-(pyridin-3-yl)-1-(tetrahydro-2*H*-pyran-3-yl)-1*H*-indazole was prepared according to the **GP**, using Ni($^{4\text{-CF3}}$stb)$_3$ (30.3 mg, 10 mol%), K_3PO_4 (114 mg, 0.536 mmol, 1.78 equiv.), 3-bromopyridine (29 µL, 0.30 mmol, 1.0 equiv.), and 5-methyl-1-(oxan-2-yl)-4-(4,4,5,5-tetramethyl-1,3,2-dioxaborolan-2-yl)-1*H*-indazole (155 mg, 0.452 mmol, 1.50 equiv.) in DMA (0.6 mL) at 60 °C. Purification *via* pTLC (silica gel, 50:50 pentane:MTBE) afforded the title compound as a yellowish oil (27.5 mg, 31%).

R**$_f$:** 0.19 (50:50 pentane:MTBE)

^{1}H NMR (400 MHz, CDCl$_3$) δ 8.69 – 8.65 (m, 2H), 7.76 – 7.71 (m, 1H), 7.66 (d, *J* = 0.9 Hz, 1H), 7.54 (dd, *J* = 8.5, 0.9 Hz, 1H), 7.44 (ddd, *J* = 7.8, 4.9, 0.9 Hz, 1H), 7.34 (d, *J* = 8.6 Hz, 1H), 5.73 (dd, *J* = 9.5, 2.7 Hz, 1H), 4.04 (ddq, *J* = 11.5, 3.4, 1.6 Hz, 1H), 3.81 – 3.70 (m, 1H), 2.57 (dddd, *J* = 13.4, 11.3, 9.4, 4.0 Hz, 1H), 2.32 (s, 3H), 2.20 – 2.12 (m, 1H), 2.12 – 2.05 (m, 1H), 1.82 – 1.62 (m, 3H).

^{13}C NMR (101 MHz, CDCl$_3$) δ 150.5, 148.7, 138.5, 137.2, 134.7, 133.0, 130.0, 129.7, 128.3, 125.6, 123.5, 109.9, 85.6, 67.7, 29.6, 25.3, 22.8, 19.3.

HRMS (EI) m/z: [M] calc'd for $C_{18}H_{19}N_3O$: 293.1523, found: 293.1525

FTIR (ATR): $\tilde{\nu}$ [cm^{-1}] = 3029, 2942, 2861, 1474, 1453, 1425, 1408, 1206, 1164, 1081, 1042, 1019, 997, 913, 808, 718.

3-(Pyridin-3-yl)quinoline (21):

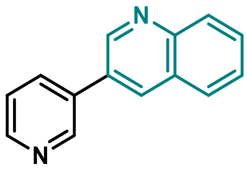

3-(Pyridin-3-yl)quinoline was prepared according to the **GP**, using Ni($^{4\text{-}CF3}$stb)$_3$ (30.3 mg, 10 mol%), K_3PO_4 (115 mg, 0.542 mmol, 1.80 equiv.), 3-bromopyridine (29 µL, 0.30 mmol, 1.0 equiv.), and quinolin-3-ylboronic acid (77.8 mg, 0.450 mmol, 1.49 equiv.) in DMA (0.6 mL) at 60 °C. Purification *via* pTLC (silica gel, 50:50 pentane:EtOAc) afforded the title compound as a yellowish solid (32.5 mg, 52%).

Spectroscopic data matched those reported in the literature.[10]

m.p.: 111.1 – 112.3 °C

***R$_f$*:** 0.22 (50:50 pentane:EtOAc)

^{1}H NMR (300 MHz, CDCl$_3$) δ 9.15 (d, *J* = 2.3 Hz, 1H), 8.97 (d, *J* = 2.3 Hz, 1H), 8.68 (dd, *J* = 4.8, 1.6 Hz, 1H), 8.31 (d, *J* = 2.3 Hz, 1H), 8.15 (d, *J* = 8.5 Hz, 1H), 8.00 (dt, *J* = 8.0, 1.9 Hz, 1H), 7.89 (dd, *J* = 8.2, 1.5 Hz, 1H), 7.75 (ddd, *J* = 8.5, 6.9, 1.5 Hz, 1H), 7.60 (ddd, *J* = 8.1, 6.9, 1.2 Hz, 1H), 7.45 (dd, *J* = 7.9, 4.9 Hz, 1H).

^{13}C NMR (75 MHz, CDCl$_3$) δ 149.24, 149.22, 148.4, 147.6, 134.6, 133.62, 133.57, 130.6, 129.9, 129.3, 128.0, 127.8, 127.3, 123.8.

6-Methoxy-3,3'-bipyridine (22):

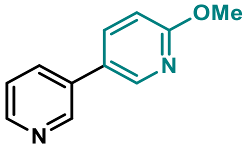

6-Methoxy-3,3'-bipyridine was prepared according to the **GP**, using Ni($^{4\text{-}CF_3}$stb)$_3$ (30.2 mg, 10 mol%), K_3PO_4 (115 mg, 0.544 mmol, 1.81 equiv.), 3-bromopyridine (29 µL, 0.30 mmol, 1.0 equiv.), and 6-methoxy-3-pyridinylboronic acid (69.2 mg, 0.453 mmol, 1.50 equiv.) in DMA (0.6 mL) at 60 °C. Purification *via* pTLC (silica gel, 70:30 pentane:MTBE) afforded the title compound as a white solid (35.0 mg, 62%).

Spectroscopic data matched those reported in the literature.[11]

m.p.: 60.3 – 61.8 °C

***R_f*:** 0.16 (70:30 pentane:MTBE)

^{1}H NMR (400 MHz, $CDCl_3$) δ 8.79 (dd, *J* = 2.4, 0.9 Hz, 1H), 8.60 (dd, *J* = 4.8, 1.6 Hz, 1H), 8.38 (dd, *J* = 2.6, 0.8 Hz, 1H), 7.85 – 7.75 (m, 2H), 7.37 (ddd, *J* = 7.9, 4.8, 0.9 Hz, 1H), 6.86 (dd, *J* = 8.6, 0.8 Hz, 1H), 3.99 (s, 3H).

^{13}C NMR (101 MHz, $CDCl_3$) δ 164.3, 148.7, 148.0, 145.3, 137.5, 134.0, 133.7, 126.9, 123.8, 111.4, 53.8.

2-Methoxy-3,3'-bipyridine (23):

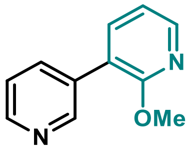

2-Methoxy-3,3'-bipyridine was prepared according to the **GP**, using Ni($^{4\text{-}CF_3}$stb)$_3$ (30.3 mg, 10 mol%), K_3PO_4 (114mg, 0.536 mmol, 1.78 equiv.), 3-bromopyridine (29 µL, 0.30 mmol, 1.0 equiv.), and 2-methoxy-3-pyridinylboronic acid (69.1 mg, 0.452 mmol, 1.50 equiv.) in DMA (0.6 mL) at 60 °C. Purification *via* pTLC (silica gel, 70:30 pentane:MTBE) followed by a basic work-up afforded the title compound as a colorless oil (22.8 mg, 41%).

Spectroscopic data matched those reported in the literature.[12]

***R_f*:** 0.35 (80:20 pentane:MTBE)

^{1}H NMR (400 MHz, $CDCl_3$) δ 8.78 (dd, *J* = 2.3, 0.9 Hz, 1H), 8.58 (dd, *J* = 4.8, 1.6 Hz, 1H), 8.21 (dd, *J* = 5.0, 1.9 Hz, 1H), 7.90 (ddd, *J* = 7.9, 2.3, 1.7 Hz, 1H), 7.63 (dd, *J* = 7.3, 1.9 Hz, 1H), 7.35 (ddd, *J* = 8.0, 4.8, 0.9 Hz, 1H), 7.01 (dd, *J* = 7.3, 5.0 Hz, 1H), 3.98 (s, 3H).

^{13}C NMR (101 MHz, $CDCl_3$) δ 161.1, 150.0, 148.7, 146.8, 138.6, 136.6, 132.7, 123.2, 121.3, 117.4, 53.7.

2-Methoxy-5-(pyridin-3-yl)pyrimidine (24):

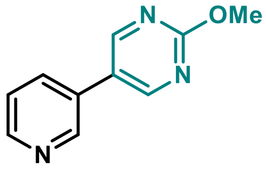

2-Methoxy-5-(pyridin-3-yl)pyrimidine was prepared according to the **GP**, using Ni($^{4\text{-}CF3}$stb)$_3$ (30.3 mg, 10 mol%), K_3PO_4 (115 mg, 0.543 mmol, 1.80 equiv.), 3-bromopyridine (29 µL, 0.3 mmol, 1.0 equiv.), and (2-methoxypyrimidin-5-yl)boronic acid (69.2 mg, 0.450 mmol, 1.49 equiv.) in DMA (0.6 mL) at 60 °C. Purification *via* pTLC (silica gel, EtOAc) afforded the title compound as an off-white solid (22.5 mg, 40%). Spectroscopic data matched those reported in the literature.[13]

m.p.: 100.8 – 112.2 °C

R**$_f$:** 0.40 (EtOAc)

^{1}H NMR (300 MHz, CDCl$_3$) δ 8.80 (d, *J* = 2.4 Hz, 1H), 8.73 (s, 2H), 8.66 (d, *J* = 4.8 Hz, 1H), 7.83 (d, *J* = 7.9 Hz, 1H), 7.42 (dd, *J* = 7.9, 4.8 Hz, 1H), 4.08 (s, 3H).

^{13}C NMR (75 MHz, CDCl$_3$) δ 165.7, 157.6, 149.6, 147.8, 134.0, 130.5, 125.4, 124.1, 55.4.

1-([3,3'-Bipyridin]-6-yl)piperidin-4-ol (25):

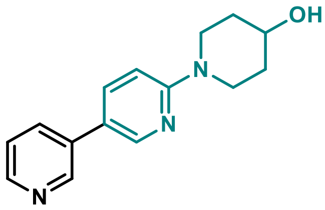

1-([3,3'-Bipyridin]-6-yl)piperidin-4-ol was prepared according to the **GP**, using Ni($^{4\text{-}CF3}$stb)$_3$ (30.3 mg, 10 mol%), K_3PO_4 (116 mg, 0.546 mmol, 1.81 equiv.), 3-bromopyridine (29 µL, 0.30 mmol, 1.0 equiv.), and 1-(5-(4,4,5,5-tetramethyl-1,3,2-dioxaborolan-2-yl)pyridin-2-yl)piperidin-4-ol (137 mg, 0.452 mmol, 1.50 equiv.) in DMA (0.6 mL) at 60 °C. Purification *via* pTLC (silica gel, 95:5 CH_2Cl_2:MeOH) followed by a basic work-up afforded the title compound as a colorless oil (24.5 mg, 32%).

R**$_f$:** 0.26 (95:5 CH_2Cl_2:MeOH)

^{1}H NMR (400 MHz, CDCl$_3$) δ 8.77 (dd, *J* = 2.4, 0.9 Hz, 1H), 8.53 (dd, *J* = 4.8, 1.6 Hz, 1H), 8.41 (dd, *J* = 2.6, 0.8 Hz, 1H), 7.80 (ddd, *J* = 7.9, 2.4, 1.6 Hz, 1H), 7.69 (dd, *J* = 8.9, 2.6 Hz, 1H), 7.33 (ddd, *J* = 7.9, 4.8, 0.9 Hz, 1H), 6.77 (dd, *J* = 8.9, 0.8 Hz, 1H), 4.17 – 4.07 (m, 2H), 3.96 (tt, *J* = 8.6, 4.0 Hz, 1H), 3.24 (ddd, *J* = 13.3, 9.8, 3.3 Hz, 2H), 2.00 (dqd, *J* = 10.5, 3.8, 1.9 Hz, 2H), 1.87 (brs, 1H), 1.68 – 1.54 (m, 2H).

^{13}C NMR (101 MHz, CDCl$_3$) δ 158.8, 147.9, 147.5, 146.4, 136.1, 134.2, 133.4, 123.8, 122.3, 107.1, 68.1, 43.0, 34.0.

HRMS (ESI) m/z: $[M+H]^+$ calc'd for $C_{15}H_{18}N_3O$: 256.1444, found: 256.1444

FTIR (ATR): $\tilde{\nu}$ [cm^{-1}] = 3286, 2933, 2855, 1605, 1548, 1506, 1476, 1427, 1365, 1321, 1300, 1229, 1077, 1025, 802, 709.

1-Methyl-5-(pyridin-3-yl)-1*H*-pyrrolo[2,3-*b*]pyridine (26):

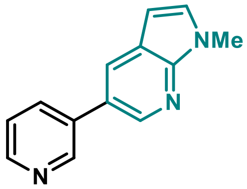

1-Methyl-5-(pyridin-3-yl)-1*H*-pyrrolo[2,3-*b*]pyridine was prepared according to the **GP**, using Ni($^{4\text{-}CF3}$stb)$_3$ (30.3 mg, 10 mol%), K_3PO_4 (113 mg, 0.534 mmol, 1.77 equiv.), 3-bromopyridine (29 µL, 0.30 mmol, 1.0 equiv.), and 1-methyl-5-(4,4,5,5-tetramethyl-1,3,2-dioxaborolan-2-yl)-1*H*-pyrrolo[2,3-*b*]pyridine (117 mg, 0.452 mmol, 1.50 equiv.) in DMA (0.6 mL) at 60 °C. Purification *via* pTLC (silica gel, 70:30 pentane:MTBE to MTBE) afforded the title compound as a yellowish oil (20.9 mg, 33%).

Spectroscopic data matched those reported in the literature.[14]

***R$_f$*:** 0.18 (MTBE)

^{1}H NMR (400 MHz, CDCl$_3$) δ 8.89 (d, *J* = 2.4 Hz, 1H), 8.61 (dd, *J* = 4.9, 1.6 Hz, 1H), 8.55 (d, *J* = 2.1 Hz, 1H), 8.09 (d, *J* = 2.1 Hz, 1H), 7.91 (dt, *J* = 7.9, 2.0 Hz, 1H), 7.39 (dd, *J* = 7.9, 4.8 Hz, 1H), 7.25 (d, *J* = 3.5 Hz, 1H), 6.53 (d, *J* = 3.5 Hz, 1H), 3.94 (s, 3H).

^{13}C NMR (101 MHz, CDCl$_3$) δ 148.6, 148.3, 147.9, 142.1, 135.5, 134.7, 130.4, 127.4, 126.0, 123.8, 120.7, 99.9, 31.6.

2-(Benzofuran-2-yl)pyrazine (27):

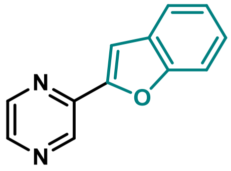

2-(Benzofuran-2-yl)pyrazine was prepared according to the **GP**, using Ni($^{4\text{-}CF3}$stb)$_3$ (30.3 mg, 10 mol%), K_3PO_4 (115 mg, 0.542 mmol, 1.81 equiv.), 2-bromopyrazine (27 µL, 0.30 mmol, 1.0 equiv.), and 2-(benzofuran-2-yl)-4,4,5,5-tetramethyl-1,3,2-dioxaborolane (110 mg, 0.451 mmol, 1.51 equiv.) in DMA (0.6 mL) at 60 °C. Purification *via* pTLC (silica gel, 50:50 pentane:EtOAc) afforded the title compound as an off-white solid (38.2 mg, 65%).

Spectroscopic data matched those reported in the literature.[3]

m.p.: 147.5 – 149.3 °C

***R_f*:** 0.6 (50:50 pentane:EtOAc)

^{1}H NMR (400 MHz, $CDCl_3$) δ 9.16 (d, *J* = 1.5 Hz, 1H), 8.61 (dd, *J* = 2.5, 1.5 Hz, 1H), 8.51 (d, *J* = 2.5 Hz, 1H), 7.67 (ddd, *J* = 7.7, 1.4, 0.8 Hz, 1H), 7.60 (dd, *J* = 8.3, 0.9 Hz, 1H), 7.51 (d, *J* = 1.0 Hz, 1H), 7.38 (ddd, *J* = 8.3, 7.2, 1.4 Hz, 1H), 7.32 – 7.25 (m, 1H).

^{13}C NMR (101 MHz, $CDCl_3$) δ 155.7, 152.6, 145.2, 144.5, 143.6, 141.5, 128.4, 126.1, 123.6, 122.0, 111.8, 107.0.

5-(Furan-3-yl)pyrimidine (28):

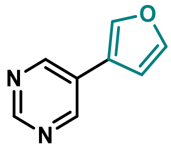

5-(Furan-3-yl)pyrimidine was prepared according to the **GP**, using Ni($^{4\text{-}CF_3}$stb)$_3$ (30.3 mg, 10 mol%), K_3PO_4 (115 mg, 0.542 mmol, 1.80 equiv.), 5-bromopyrimidine (47.7 mg, 0.300 mmol, 1.00 equiv.), and 3-furanylboronic acid (50.4 mg, 0.450 mmol, 1.50 equiv.) in DMA (0.6 mL) at 60 °C. Purification *via* pTLC (silica gel, 50:50 pentane:EtOAc) afforded the title compound as an off-white solid (28.2 mg, 64%). Spectroscopic data matched those reported in the literature.[3]

m.p.: 71.5 – 72.8 °C

***R_f*:** 0.35 (50:50 pentane:EtOAc)

^{1}H NMR (300 MHz, $CDCl_3$) δ 9.12 (s, 1H), 8.85 (s, 2H), 7.83 (dd, *J* = 1.5, 0.9 Hz, 1H), 7.61 – 7.52 (m, 1H), 6.72 (dd, *J* = 1.9, 0.9 Hz, 1H).

^{13}C NMR (75 MHz, $CDCl_3$) δ 157.2, 153.9, 144.9, 139.6, 126.9, 120.0, 108.2.

Methyl 5-(furan-3-yl)nicotinate (29):

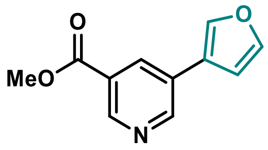

Methyl 5-(furan-3-yl)nicotinate was prepared according to the **GP**, using Ni($^{4\text{-}CF_3}$stb)$_3$ (30.3 mg, 10 mol%), K_3PO_4 (115 mg, 0.542 mmol, 1.81 equiv.), methyl 5-bromonicotinate (64.9 mg, 0.300 mmol, 1.00 equiv.), and 3-furanylboronic acid (50.4 mg, 0.450 mmol, 1.50 equiv.) in DMA (0.6 mL) at 60 °C. Purification *via* pTLC (silica gel, 50:50 pentane:EtOAc) afforded the title compound as an off-white solid (37.2 mg, 61%).

m.p.: 70.4 – 72.1 °C

***R_f*:** 0.55 (50:50 pentane:EtOAc)

^{1}H NMR (300 MHz, CDCl$_3$) δ 9.07 (d, J = 2.0 Hz, 1H), 8.89 (d, J = 2.2 Hz, 1H), 8.33 (t, J = 2.1 Hz, 1H), 7.83 (dd, J = 1.6, 0.9 Hz, 1H), 7.52 (t, J = 1.7 Hz, 1H), 6.74 (dd, J = 1.9, 0.9 Hz, 1H), 3.96 (s, 3H).
^{13}C NMR (101 MHz, CDCl$_3$) δ 165.8, 150.6, 149.0, 144.5, 139.6, 133.9, 128.5, 126.2, 122.5, 108.5, 52.6.
HRMS (EI) m/z: [M] calc'd for $C_{11}H_9NO_3$: 203.0577, found: 203.0578
FTIR (ATR): $\tilde{\nu}$ [cm^{-1}] = 3099, 2952, 2837, 1721, 1589, 1510, 1426, 1316, 1262, 1156, 1120, 1023, 993, 875, 834, 764, 696, 597, 425.

3-(6-Methoxypyridin-3-yl)-1,5-naphthyridine (30):

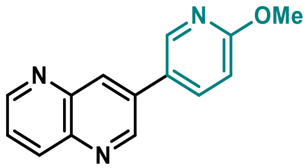

3-(6-Methoxypyridin-3-yl)-1,5-naphthyridine was prepared according to the **GP**, using Ni($^{4\text{-}CF3}$stb)$_3$ (30.3 mg, 10 mol%), K_3PO_4 (115 mg, 0.542 mmol, 1.81 equiv.), 3-bromo-1,5-naphthyridine (62.7 mg, 0.300 mmol, 1.00 equiv.), and 6-methoxy-3-pyridinylboronic acid (68.8 mg, 0.450 mmol, 1.50 equiv.) in DMA (0.6 mL) at 60 °C. Purification *via* pTLC (silica gel, 70:30 pentane:EtOAc) afforded the title compound as a white solid (40.1 mg, 56%).
m.p.: 158.4 – 160.5 °C
***R$_f$*:** 0.35 (70:30 pentane:EtOAc)
^{1}H NMR (300 MHz, CDCl$_3$) δ 9.18 (d, J = 2.3 Hz, 1H), 8.99 (dd, J = 4.2, 1.7 Hz, 1H), 8.55 (dd, J = 2.6, 0.8 Hz, 1H), 8.49 (dd, J = 2.3, 0.9 Hz, 1H), 8.41 (ddd, J = 8.5, 1.7, 0.9 Hz, 1H), 7.93 (dd, J = 8.6, 2.6 Hz, 1H), 7.63 (dd, J = 8.5, 4.2 Hz, 1H), 6.91 (dd, J = 8.6, 0.7 Hz, 1H), 4.00 (s, 3H).
^{13}C NMR (101 MHz, CDCl$_3$) δ 164.6, 151.9, 150.1, 145.8, 143.8, 142.9, 137.6, 137.3, 134.3, 133.7, 126.1, 124.3, 111.7, 53.9.
HRMS (EI) m/z: [M] calc'd for $C_{14}H_{11}N_3O$: 237.0897, found: 237.0899
FTIR (ATR): $\tilde{\nu}$ [cm^{-1}] = 3051. 2975, 2941, 2828, 1609, 1470, 1387, 1365, 1265, 1233, 1201, 1080, 1021, 850, 735, 704, 510, 461, 416.

6'-Methoxy-5-(trifluoromethyl)-2,3'-bipyridine (31):

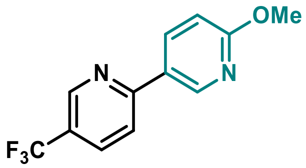

6'-Methoxy-5-(trifluoromethyl)-2,3'-bipyridine was prepared according to the **GP**, using Ni($^{4\text{-}CF3}$stb)$_3$ (30.3 mg, 10 mol%), K_3PO_4 (114 mg, 0.540 mmol, 1.80 equiv.), 2-bromo-5-(trifluoromethyl)pyridine (67.8 mg, 0.300 mmol, 1.00 equiv.), and 6-methoxy-3-pyridinylboronic acid (68.8 mg, 0.450 mmol, 1.50 equiv.) in DMA (0.6 mL) at 60 °C. Purification *via* pTLC (silica gel, 98:2 CH_2Cl_2:MTBE) afforded the title compound as a white solid (53.0 mg, 69%).

Spectroscopic data matched those reported in the literature.[13]

m.p.: 80.0 – 83.5 °C

***R_f*:** 0.41 (98:2 CH_2Cl_2:MTBE)

^{1}H NMR (400 MHz, $CDCl_3$) δ 8.92 (dt, *J* = 2.5, 1.0 Hz, 1H), 8.81 (dd, *J* = 2.5, 0.8 Hz, 1H), 8.29 (dd, *J* = 8.7, 2.5 Hz, 1H), 7.97 (ddd, *J* = 8.4, 2.0, 0.8 Hz, 1H), 7.78 (dt, *J* = 8.5, 0.8 Hz, 1H), 6.87 (dd, *J* = 8.7, 0.7 Hz, 1H), 4.01 (s, 3H).

^{13}C NMR (101 MHz, $CDCl_3$) δ 165.4, 158.4, 146.9 (q, *J* = 4.1 Hz), 146.3, 137.6, 134.1 (q, *J* = 3.5 Hz), 127.3, 124.8 (q, *J* = 33.2 Hz), 123.8 (q, *J* = 272.0 Hz), 119.1, 111.3, 53.9.

^{19}F NMR (282 MHz, $CDCl_3$) δ −62.31.

2-(Benzo[*b*]thiophen-3-yl)-5-(trifluoromethyl)pyridine (32):

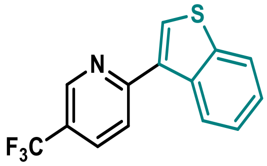

2-(Benzo[*b*]thiophen-3-yl)-5-(trifluoromethyl)pyridine was prepared according to the **GP**, using Ni($^{4\text{-}CF3}$stb)$_3$ (30.3 mg, 10 mol%), K_3PO_4 (115 mg, 0.541 mmol, 1.80 equiv.), 2-bromo-5-(trifluoromethyl)pyridine (67.8 mg, 0.300 mmol, 1.00 equiv.), and benzo[*b*]thien-2-ylboronic acid (80.2 mg, 0.451 mmol, 1.50 equiv.) in DMA (0.6 mL) at 60 °C. Purification *via* pTLC (silica gel, 98:2 Pent:MTBE) afforded the title compound as a white solid (59.7 mg, 71%).

m.p.: 84.5 – 85.5 °C

***R_f*:** 0.28 (98:2 Pent:MTBE)

^{1}H NMR (400 MHz, $CDCl_3$) δ 9.03 – 9.01 (m, 1H), 8.54 (ddd, *J* = 8.1, 1.5, 0.7 Hz, 1H), 8.01 (dd, *J* = 8.4, 2.4 Hz, 1H), 7.96 – 7.91 (m, 2H), 7.81 (d, *J* = 8.3 Hz, 1H), 7.53 – 7.40 (m, 2H).

^{13}C NMR (101 MHz, $CDCl_3$) δ 157.9, 146.7 (q, J = 4.1 Hz), 141.0, 136.9, 135.4, 133.9 (q, J = 3.4 Hz), 128.5, 125.2, 125.1, 124.7 (q, J = 33.1 Hz) 124.3, 123.9 (q, J = 272.1 Hz), 123.0, 122.0.

^{19}F NMR (282 MHz, $CDCl_3$) δ −62.23.

HRMS (EI) m/z: [M] calc'd for $C_{14}H_8NSF_3$: 279.0324, found: 279.0326

FTIR (ATR): $\tilde{\nu}$ [cm^{-1}] = 3096, 3065, 2924, 1603, 1323, 1226, 1163, 1121, 1078, 1012, 859, 840, 762, 745, 732, 421, 407.

Limitations of the Scope

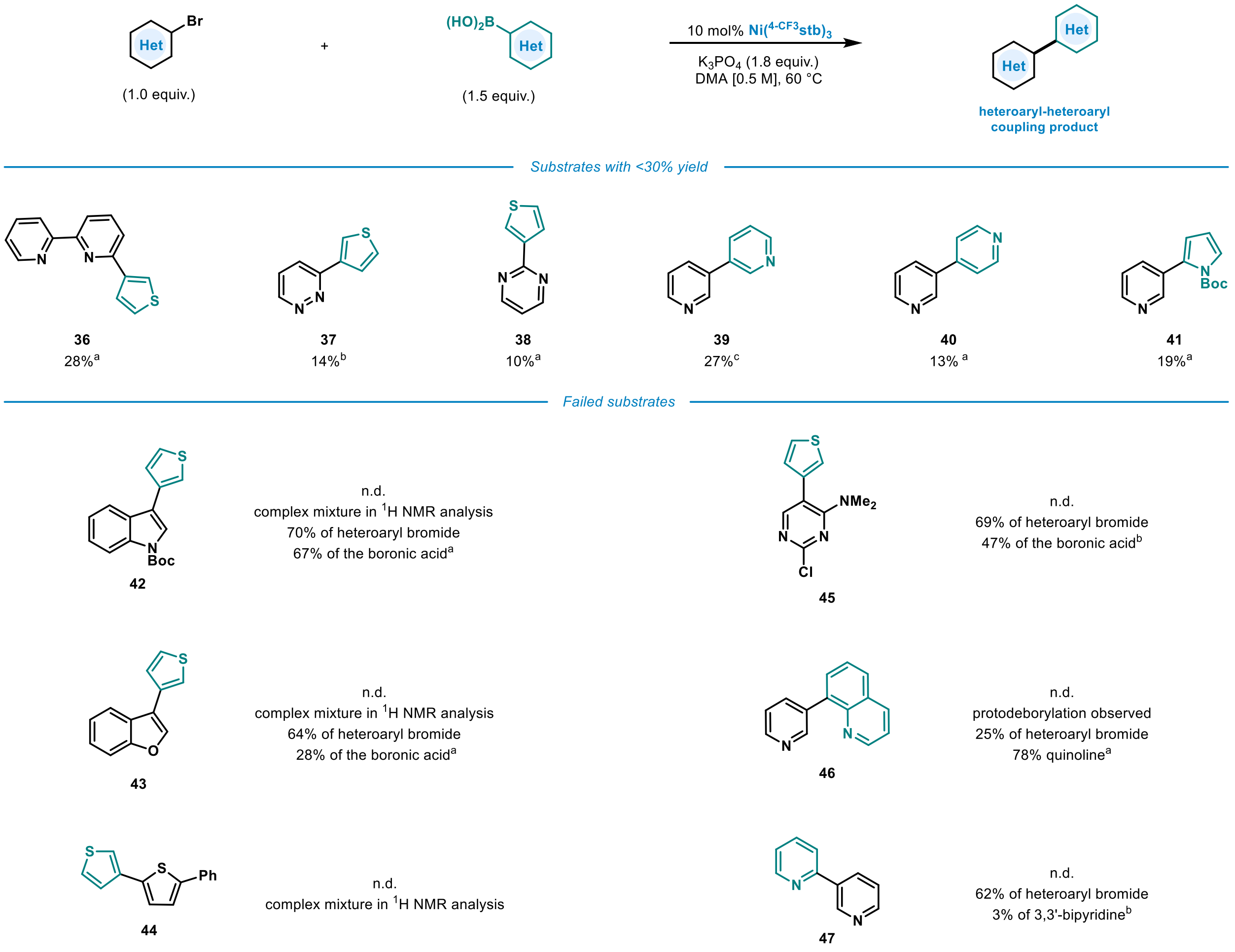

[a] ^{1}H NMR yield as determined by using 1,3,5-trimethoxybenzene as internal standard. [b] ^{1}H NMR yield as determined by using $^{4\text{-}CF3}$stb as internal standard.
[c] ^{1}H NMR yield as determined by using CBr_2H_2 as internal standard. n.d. = not detected

Note: All results shown here are based on ^{1}H NMR analysis after aqueous work-up.

Addition of Water

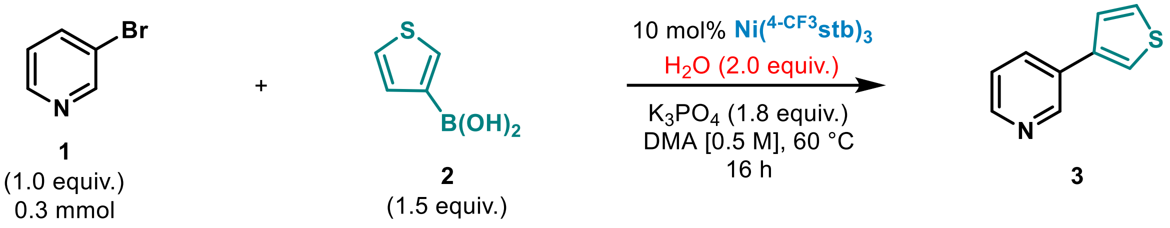

A 12-mL screwcap vial equipped with a stirring bar was dried under vacuum using a heat gun set to 550 °C, followed by three cycles of vacuum/argon. The vial was charged with Ni($^{4\text{-}CF_3}$stb)$_3$ (30.3 mg,10 mol%), K_3PO_4 (115 mg, 0.540 mmol, 1.79 equiv.) and with and 3-thienylboronic acid (57.8 mg, 0.452 mmol, 1.50 equiv.) weighed under air. One cycle of vacuum/argon was performed. The reaction vessel was charged with 3-bromopyridine (29 µL, 0.30 mmol, 1.0 equiv.) and water (11 µL, 0.61 mmol, 2.0 equiv.) using Hamilton syringes through the rubber septum, while bubbling argon through the liquid materials. DMA (0.6 mL) was added using a syringe through the rubber septum, the cap of the vial was switched for a new one, and the reaction tube was placed into a pre-heated heating block held at 60 °C. The reaction was stirred for 16 h at that temperature and was then allowed to cool to 25 °C. The reaction was diluted with MTBE, and quenched by the addition of a saturated aqueous solution of LiCl (10 mL). The phases were separated and the aqueous layer was extracted with MTBE (4 × 10 mL). The combined organic layers were dried over $MgSO_4$ and concentrated under reduced pressure. 1,3,5-Trimethoxybenzene was added as internal standard, the mixture was dissolved in $CDCl_3$ and an aliquot was transferred into an NMR tube. The yield of **3** was determined to be 87%.

Zero-Precaution Experiment: Reaction under Air

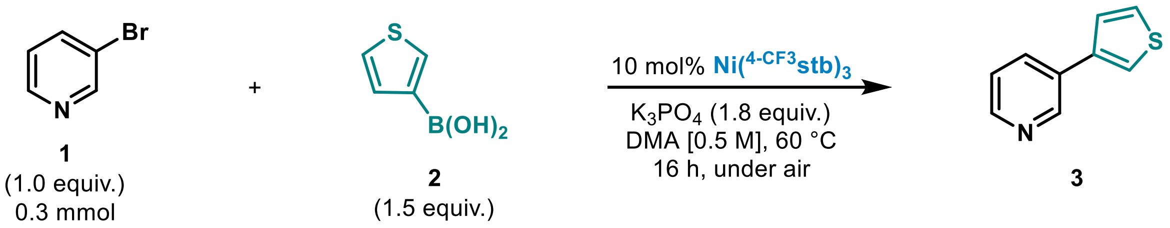

An oven-dried 12-mL screwcap vial equipped with a stirring bar was charged with Ni($^{4\text{-}CF_3}$stb)$_3$ (30.3 mg,10 mol%), K_3PO_4 (115 mg, 0.540 mmol, 1.79 equiv.) and 3-thienylboronic acid (57.8 mg, 0.452 mmol, 1.50 equiv.) weighed under air. The reaction vessel was charged with 3-bromopyridine (29 µL, 0.30 mmol, 1.0 equiv.) using a Hamilton syringe through the rubber septum. DMA which was stored without any precautions on the benchtop (0.6 mL) was added using a syringe through the rubber septum, the cap of the vial was switched for a new one, and the reaction tube was placed into a pre-heated heating block held at 60 °C. The reaction was stirred for 16 h at that temperature and was then allowed to cool to 25 °C. The

reaction was diluted with MTBE, and quenched by the addition of a saturated aqueous solution of LiCl (10 mL). The phases were separated and the aqueous layer was extracted with MTBE (4 × 10 mL). The combined organic layers were dried over $MgSO_4$ and concentrated under reduced pressure. 1,3,5-Trimethoxybenzene was added as internal standard, the mixture was dissolved in $CDCl_3$ and an aliquot was transferred into an NMR tube. The yield of **3** was determined to be 25%.

Zero-Precaution Experiment: Argon-Flush

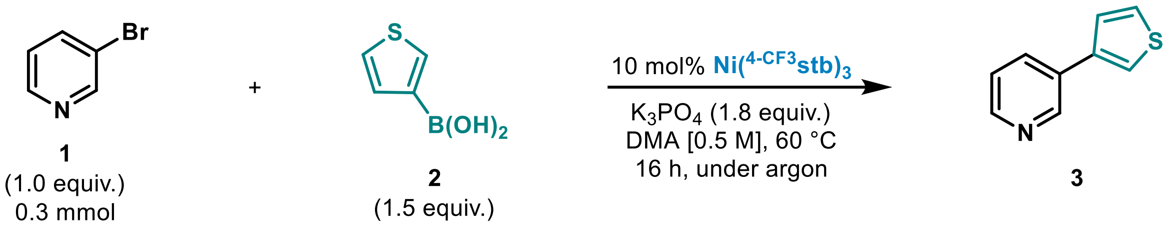

An oven-dried 12-mL screwcap vial equipped with a stirring bar was flushed with argon for 10 min and charged with Ni($^{4\text{-}CF3}$stb)$_3$ (30.3 mg,10 mol%), K_3PO_4 (115 mg, 0.540 mmol, 1.79 equiv.) and 3-thienylboronic acid (57.8 mg, 0.452 mmol, 1.50 equiv.) weighed under air. The reaction vessel was flushed with argon for 3 min and was then charged with 3-bromopyridine (29 µL, 0.30 mmol, 1.0 equiv.) using a Hamilton syringe through the rubber septum, while purging the reagent with argon. DMA, which was stored without any precautions on the benchtop (0.6 mL), was added using a syringe through the rubber septum while purging the solvent with argon, the cap of the vial was switched for a new one, and the reaction tube was placed into a pre-heated heating block held at 60 °C. The reaction was stirred for 16 h at that temperature and was then allowed to cool down to 25 °C. The reaction was diluted with MTBE, and quenched by the addition of a saturated aqueous solution of LiCl (10 mL). The phases were separated and the aqueous layer was extracted with MTBE (4 × 10 mL). The combined organic layers were dried over $MgSO_4$ and concentrated under reduced pressure. 1,3,5-Trimethoxybenzene was added as internal standard, the mixture was dissolved in $CDCl_3$ and an aliquot was transferred into an NMR tube. The yield of **3** was determined to be 89%.

Scale-Up of 4-(thiophen-3-yl)isoquinoline (10)

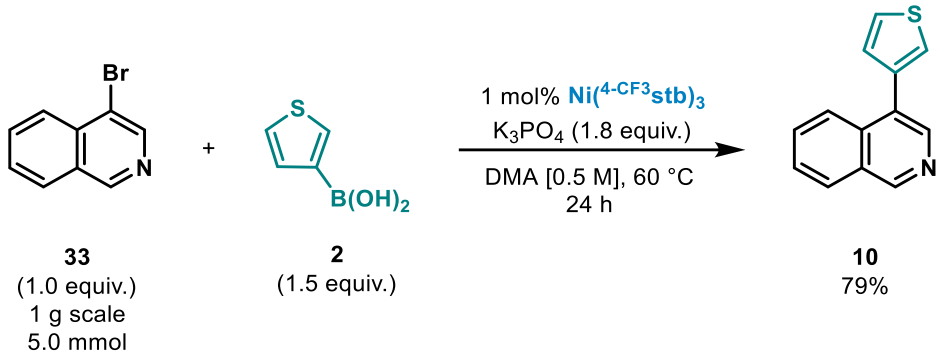

A 100-mL flat-bottomed tube equipped with a cross-shaped stirring bar was capped with a rubber septum and dried under vacuum using a heat gun set to 550 °C, followed by three cycles of vacuum/argon. The vial was charged with Ni($^{4\text{-}CF_3}$stb)$_3$ (50.4 mg, 1 mol%), K_3PO_4 (1.91 g, 9.00 mmol, 1.80 equiv.), 4-bromoisoquinoline (1.0 g, 5.0 mmol, 1.0 equiv.), and 3-thienylboronic acid (0.96 g, 7.50 mmol, 1.50 equiv.), weighed under air. One cycle of vacuum/argon was performed. DMA (10 mL) was added using a syringe and the reaction tube was placed into a pre-heated oil bath held at 60 °C. The reaction was vigorously stirred for 24 h at that temperature and was then allowed to cool to 25 °C. The reaction was diluted with MTBE (15 mL), and quenched by the addition of a saturated aqueous solution of LiCl (150 mL). The phases were separated and the aqueous layer was extracted with MTBE (3 × 200 mL). The combined organic layers were dried over $MgSO_4$ and concentrated under reduced pressure. The residue was dissolved in MTBE and dry-loaded onto Celite. Purification *via* column chromatography (silica gel, 70:30 pentane/MTBE) afforded the title compound as a colorless oil (0.83 g, 79%).

Scale-Up of 2-Methoxy-5-(pyridin-3-yl)pyrimidine (24):

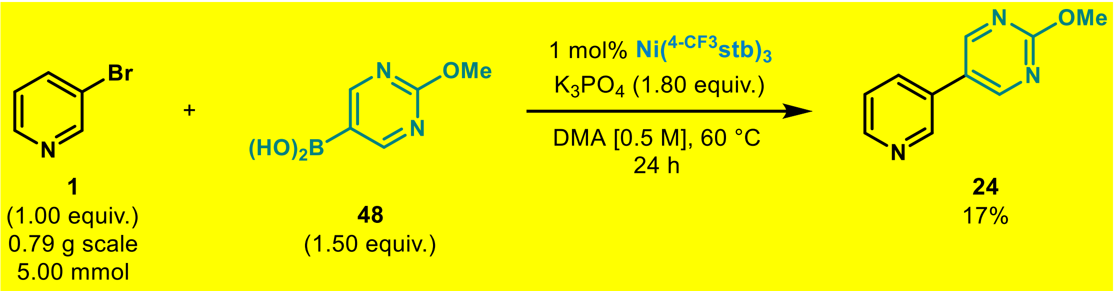

A 100-mL flat-bottomed tube equipped with a cross-shaped stirring bar was capped with a rubber septum and dried under vacuum using a heat gun set to 550 °C, followed by three cycles of vacuum/argon. The vial was charged with Ni($^{4\text{-}CF_3}$stb)$_3$ (50.4 mg, 1 mol%), K_3PO_4 (1.91 g, 9.00 mmol, 1.80 equiv.), 3-bromopyridine (790 mg, 0.482 mL, 5.00 mmol, 1.00 equiv.), and (2-methoxypyrimidin-5-yl)boronic acid (1.15 g, 7.50 mmol, 1.50 equiv.), weighed under air. One cycle of vacuum/argon was performed. DMA

(10 mL) was added using a syringe and the reaction tube was placed into a pre-heated oil bath held at 60 °C. The reaction was vigorously stirred for 24 h at that temperature and was then allowed to cool to 25 °C. The reaction was diluted with EtOAc (15 mL), and quenched by the addition of a saturated aqueous solution of LiCl (150 mL). The phases were separated and the aqueous layer was extracted with EtOAc (3 × 200 mL). The combined organic layers were dried over $MgSO_4$ and concentrated under reduced pressure. Purification *via* column chromatography (silica gel, EtOAc) afforded the title compound as a white solid (0.159 g, 17%).

Ligand-Exchange Study in Ni($^{4\text{-}CF_3}$stb)$_3$ with 3-bromopyridine:

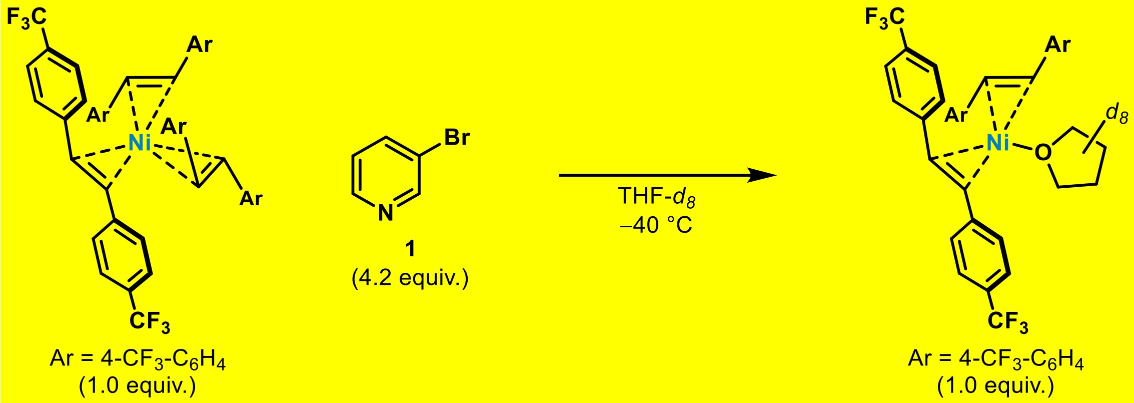

A 12-mL screwcap vial was dried under vacuum using a heat gun set to 550 °C, followed by three cycles of vacuum/argon. The vial was charged with Ni($^{4\text{-}CF_3}$stb)$_3$ (20.0 mg, 0.020 mmol, 1.00 equiv.) and 3-bromopyridine (8.0 µL, 0.08 mmol, 4.2 equiv.) at −80 °C. THF-d_8 (1.0 mL) was added at this temperature and an aliquot was transferred into an NMR tube held under argon. NMR analysis was performed at −40 °C.

^{1}H NMR (600 MHz, THF-d_8, 233K) δ 8.68 (s, 1H), 8.53 (s, 1H), 7.99 (s, 1H), 7.84 (s, 2H), 7.74 (s, 5H), 7.52 (s, 1H), 7.32 (s, 1H), 6.20 (s, 1H), 4.61 – 4.58 (m, 1H), 4.27 (s, 1H).

^{13}C NMR (151 MHz, THF-d_8, 233K) δ 151.7, 149.1, 141.7, 130.4, 128.1, 121.6, 73.5, 70.6.

^{19}F NMR (565 MHz, THF-d_8, 233K) δ −62.56, −62.76, −62.94.

Note: Due to bad signal quality detailed characterization of the reaction mixture was not possible. However some observations made in the spectra hint toward the formation of Ni($^{4\text{-}CF_3}$stb)$_2$(THF-d_8). In ^{1}H NMR analysis broad signals were detected (6.20 (s, 1H), 4.61 – 4.58 (m, 1H), 4.27 (s, 1H) ppm), resembling those previously reported in a variable temperature NMR of Ni($^{4\text{-}CF_3}$stb)$_3$ in THF-d_8.[1a] Moreover, in ^{13}C NMR, signals at 151.7, 149.1, 141.7, 121.6 ppm were assigned to uncoordinated **1**, the signal at 130.4 ppm was assigned to uncoordinated $^{3,5\text{-}CF_3}$stb and the signal at 128.1 ppm was assigned to Ni($^{4\text{-}CF_3}$stb)$_2$(THF-d_8). Based on these results we speculate that no ligand exchange with 3-bromopyridine occurred, instead an exchange with one molecule of THF-d_8 results in the formation of Ni($^{4\text{-}CF_3}$stb)$_2$(THF-d_8). This might be a result of the high concentration difference between 3-bromopyridine and THF-d_8, and might reflect the situation in catalysis which involves similar conditions. However, at this point we cannot discard the possibility of

substrate coordination within the catalytic cycle as a fast equilibrium between solvent and substrate coordination might be in place.

^{1}H NMR of the ligand exchange study (600 MHz, THF-d_8, 233K)

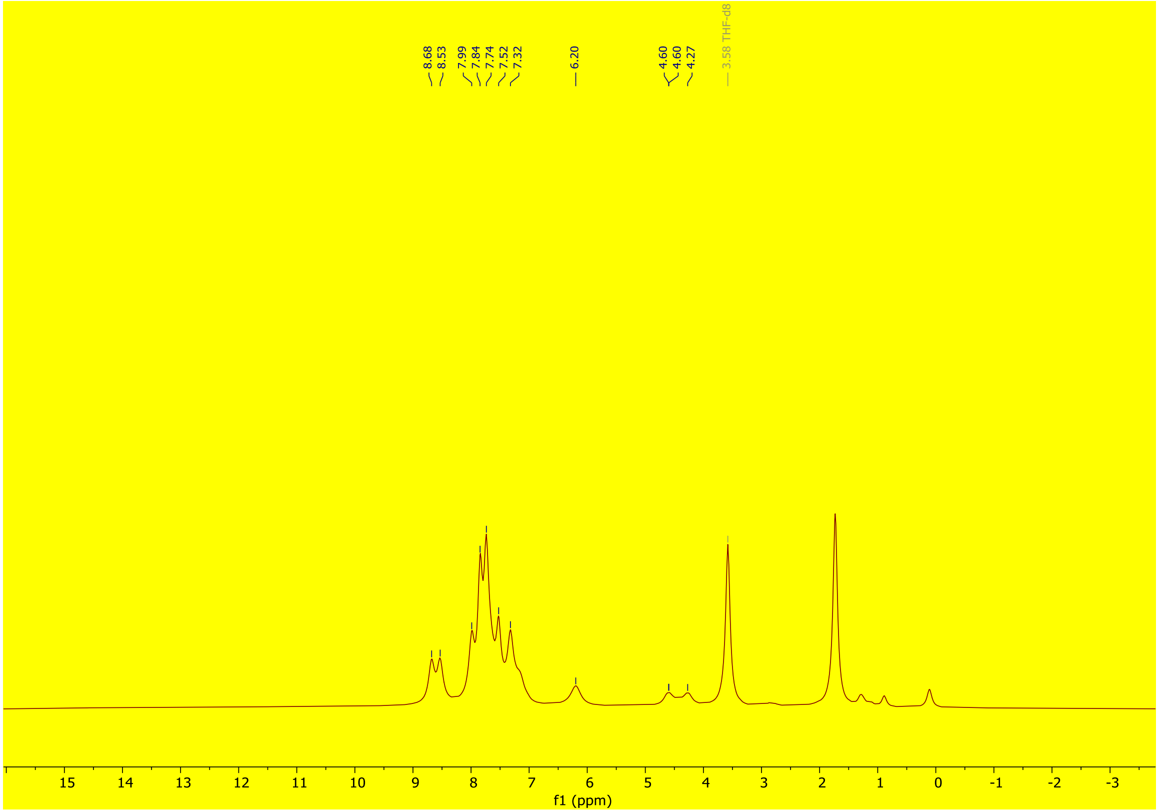

^{13}C NMR of the ligand exchange study (151 MHz, THF-d_8, 233K)

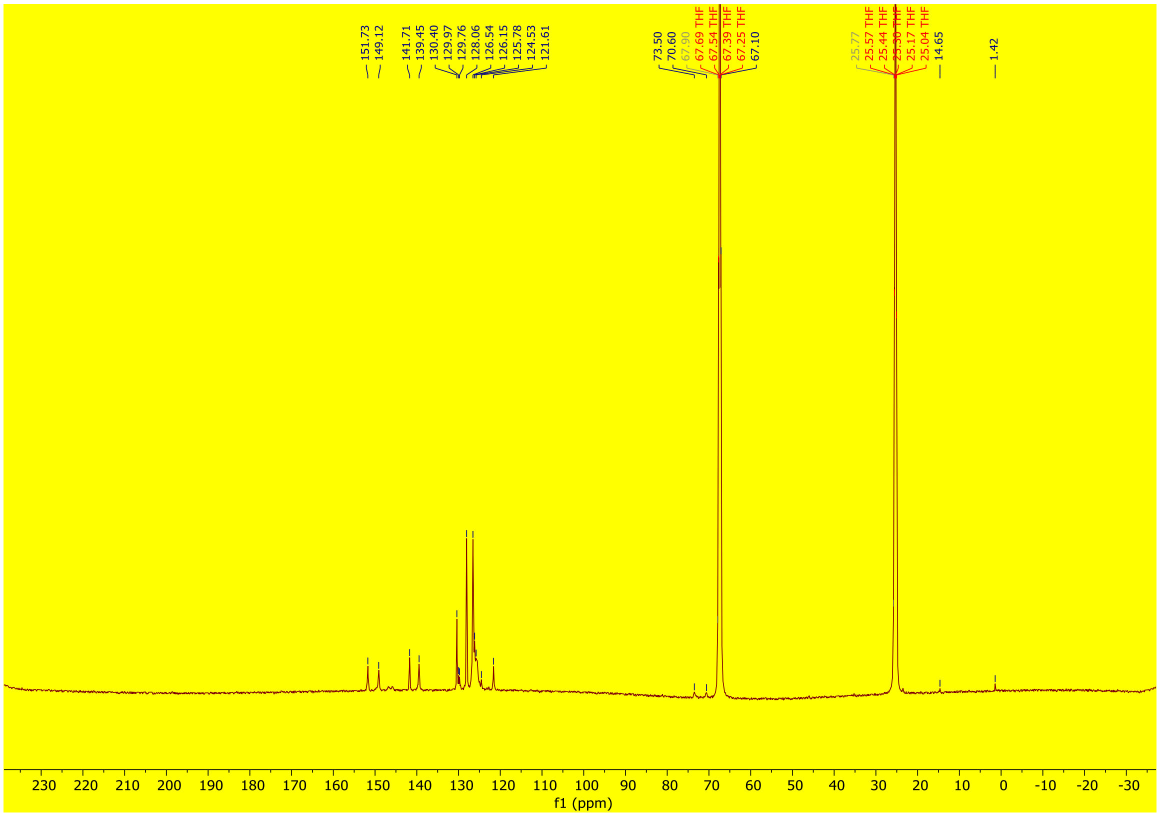

^{19}F NMR of the ligand exchange study (565 MHz, THF-d_8, 233K)

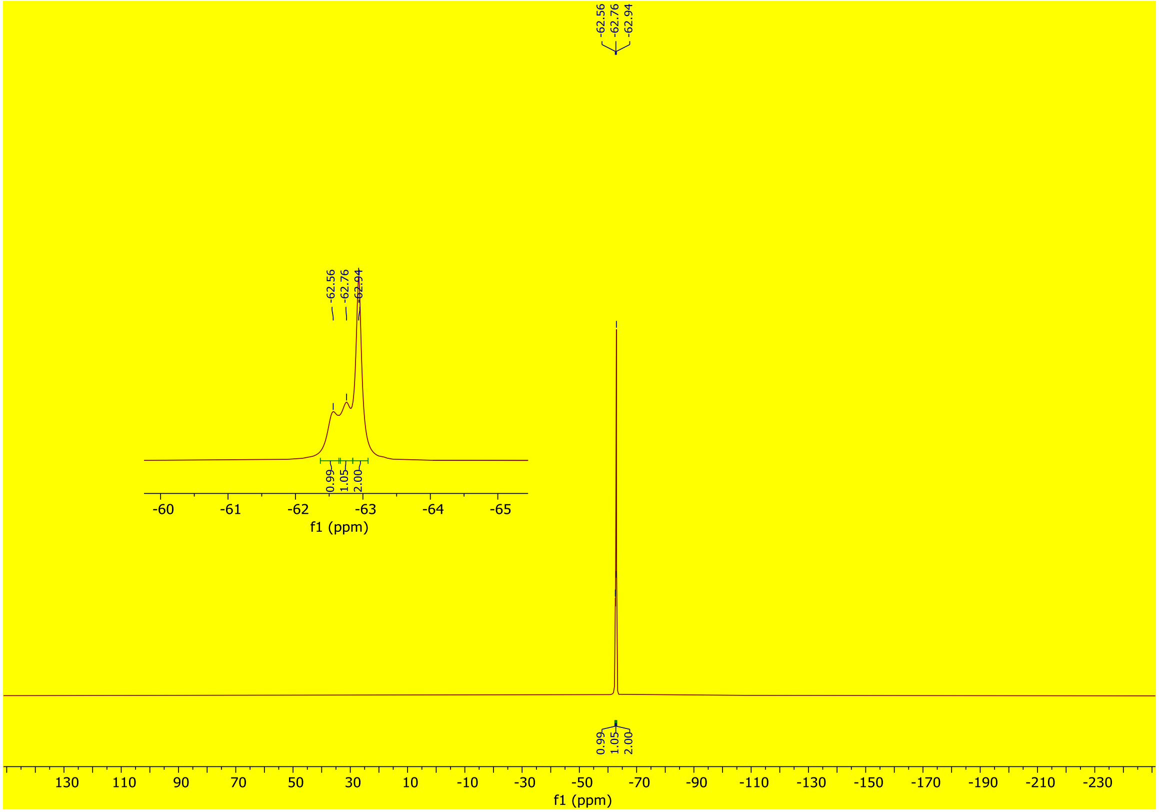

References

[1] a) L. Nattmann, R. Saeb, N. Nöthling, J. Cornella, *Nat. Catal.* **2020**, *3*, 6–13; b) R. Saeb, B. Boulenger, J. Cornella, *Org. Lett.* **2024**, *26*, 5928–5933.

[2] N. Oka, T. Yamada, H. Sajiki, S. Akai, T. Ikawa, *Org. Lett.* **2022**, *24*, 3510–3514.

[3] S. Ge, J. F. Hartwig, *Angew. Chem., Int. Ed.* **2012**, *51*, 12837–12841; *Angew. Chem.* **2012**, *124*, 13009–13013.

[4] B. H. Lipshutz, A. R. Abela, *Org. Lett.* **2008**, *10*, 5329–5332.

[5] J.-S. Ouyang, Y.-F. Li, F.-D. Huang, D.-D. Lu, F.-S. Liu, *ChemCatChem* **2018**, *10*, 371–375.

[6] K. M. Dawood, A. Kirschning, *Tetrahedron* **2005**, *61*, 12121–12130.

[7] M. Kuriyama, S. Matsuo, M. Shinozawa, O. Onomura, *Org. Lett.* **2013**, *15*, 2716–2719.

[8] R. R. Surgenor, H. Lee, *Chem. – Eur. J.* **2024**, *30*, e202401552.

[9] S. Bernhardt, Z.-L. Shen, P. Knochel, *Chem. – Eur. J.* **2013**, *19*, 828–833.

[10] X. Chen, L. Zhou, Y. Li, T. Xie, S. Zhou, *J. Org. Chem.* **2014**, *79*, 230–239.

[11] P. Bhattacharjee, A. Dewan, P. K. Boruah, M. R. Das, S. P. Mahanta, A. J. Thakur, U. Bora, *Green Chem.* **2022**, *24*, 7208–7219.

[12] M. A. Pena, J. Pérez Sestelo, L. A. Sarandeses, *J. Org. Chem.* **2007**, *72*, 1271–1275.

[13] T. Markovic, B. N. Rocke, D. C. Blakemore, V. Mascitti, M. C. Willis, *Chem. Sci.* **2017**, *8*, 4437–4442.

[14] S. Maiti, P. Ghosh, D. Raja, S. Ghosh, S. Chatterjee, V. Sankar, S. Roy, G. K. Lahiri, D. Maiti, S. Maiti, P. Ghosh, D. Raja, S. Ghosh, S. Chatterjee, V. Sankar, S. Roy, G. K. Lahiri, D. Maiti, *Nat. Catal.* **2024**, *7*, 285–294.

NMR Spectra

^{1}H NMR of $^{4\text{-}CF3}$stb ($CDCl_3$, 400 MHz)

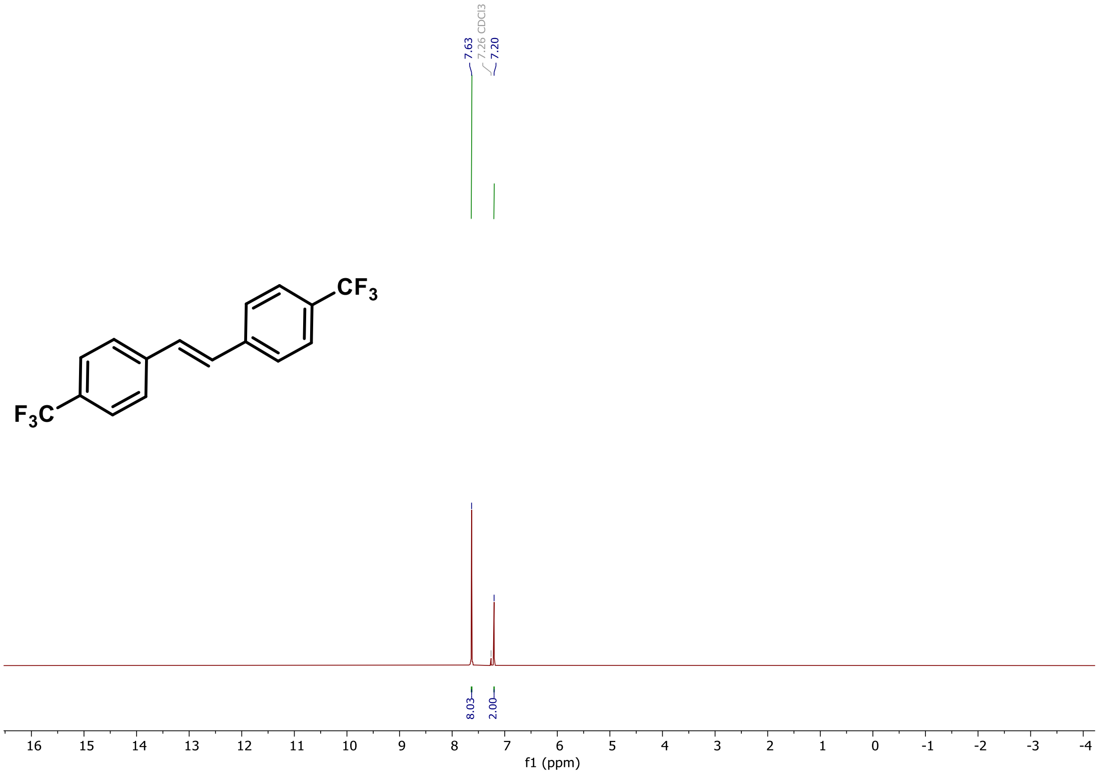

^{13}C NMR of $^{4\text{-}CF3}$stb ($CDCl_3$, 101 MHz)

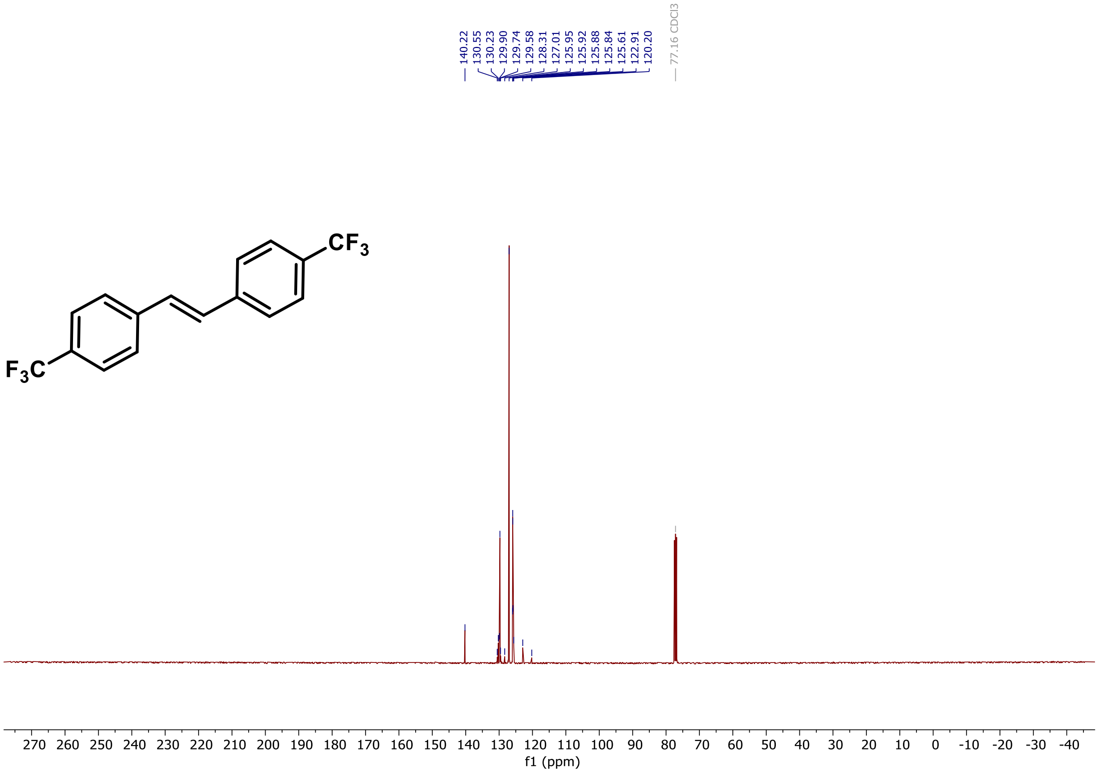

^{19}F NMR of $^{4\text{-}CF3}$stb (CDCl$_3$, 282 MHz)

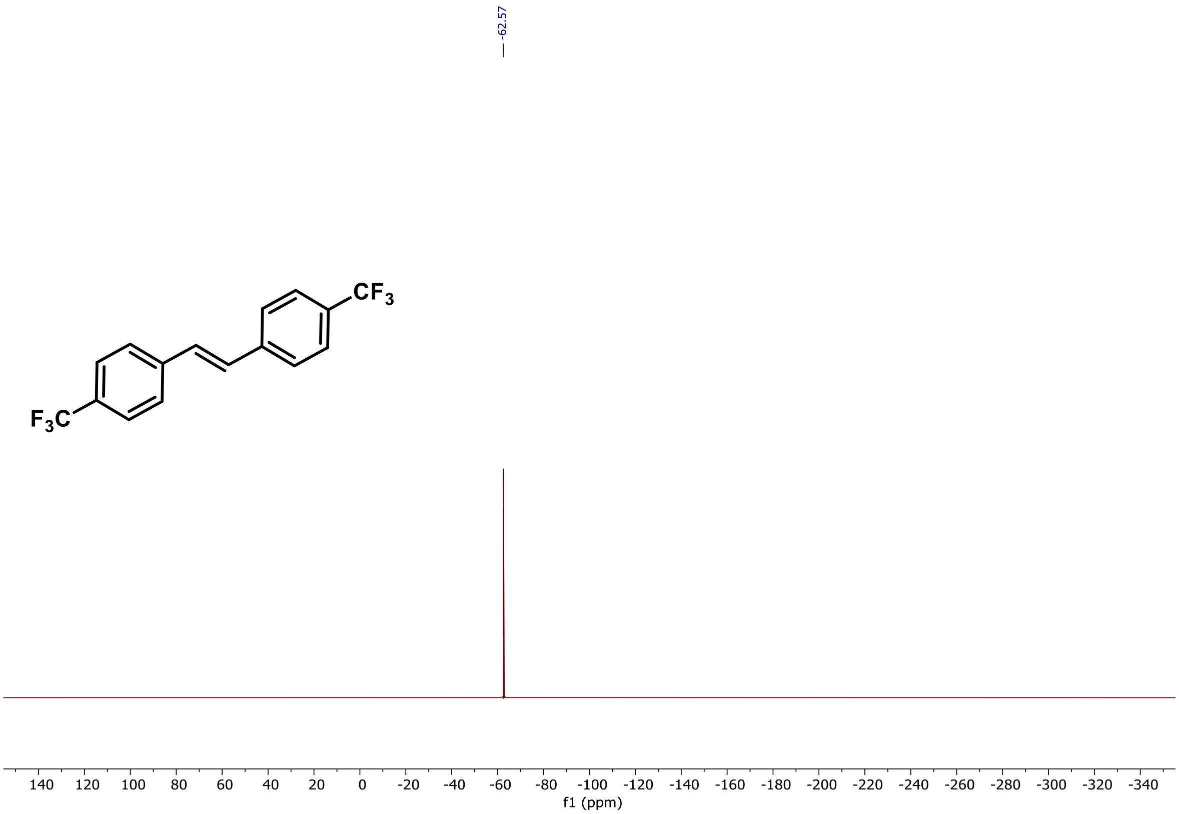

^{1}H NMR of SM1 ($CDCl_3$, 400 MHz)

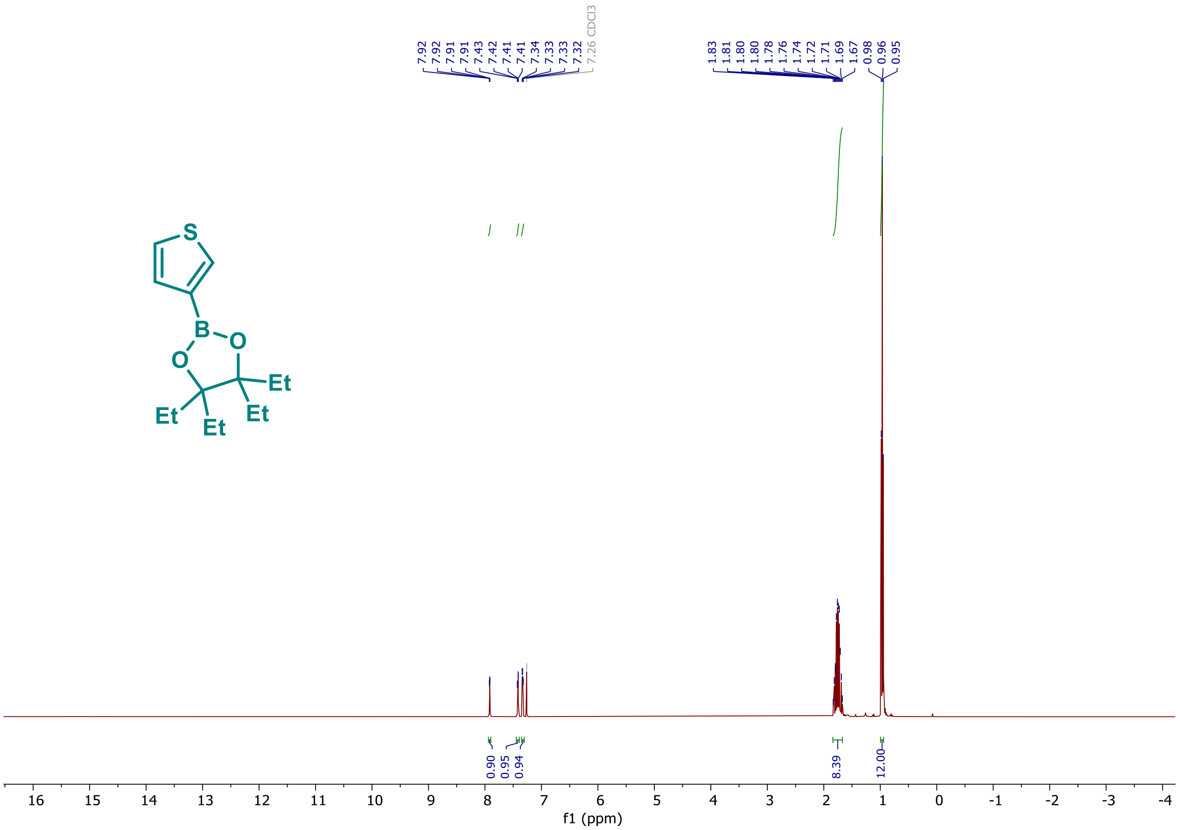

^{13}C NMR of SM1 ($CDCl_3$, 101 MHz)

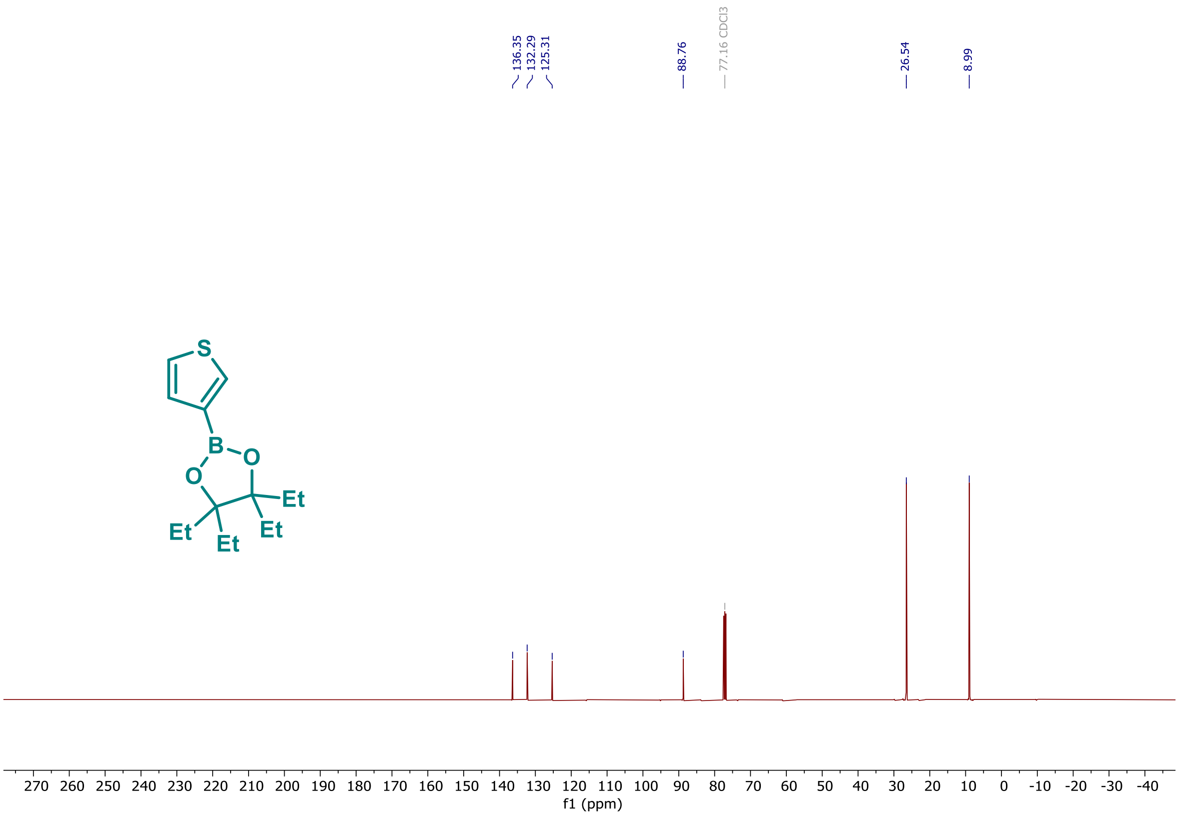

^{11}B NMR of SM1 ($CDCl_3$, 128 MHz)

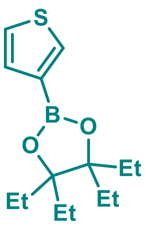

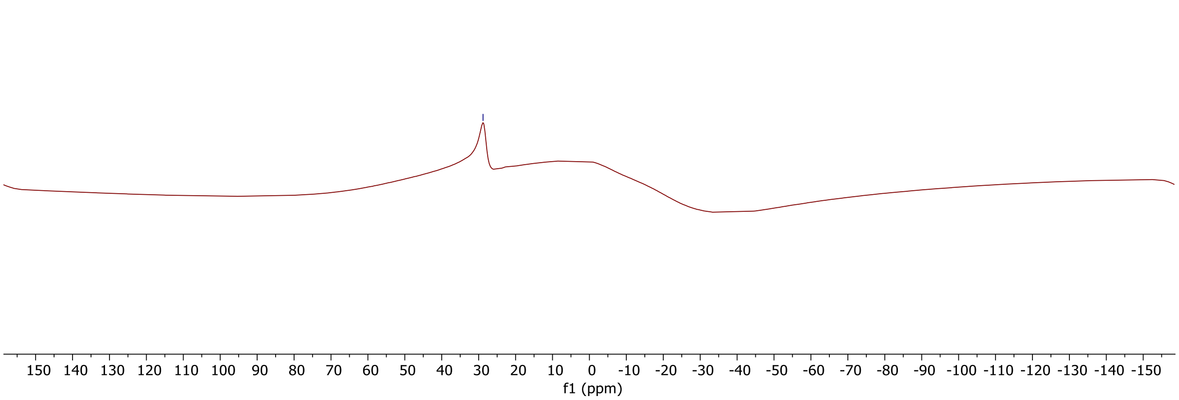

^{1}H NMR of SM2 ($CDCl_3$, 400 MHz)

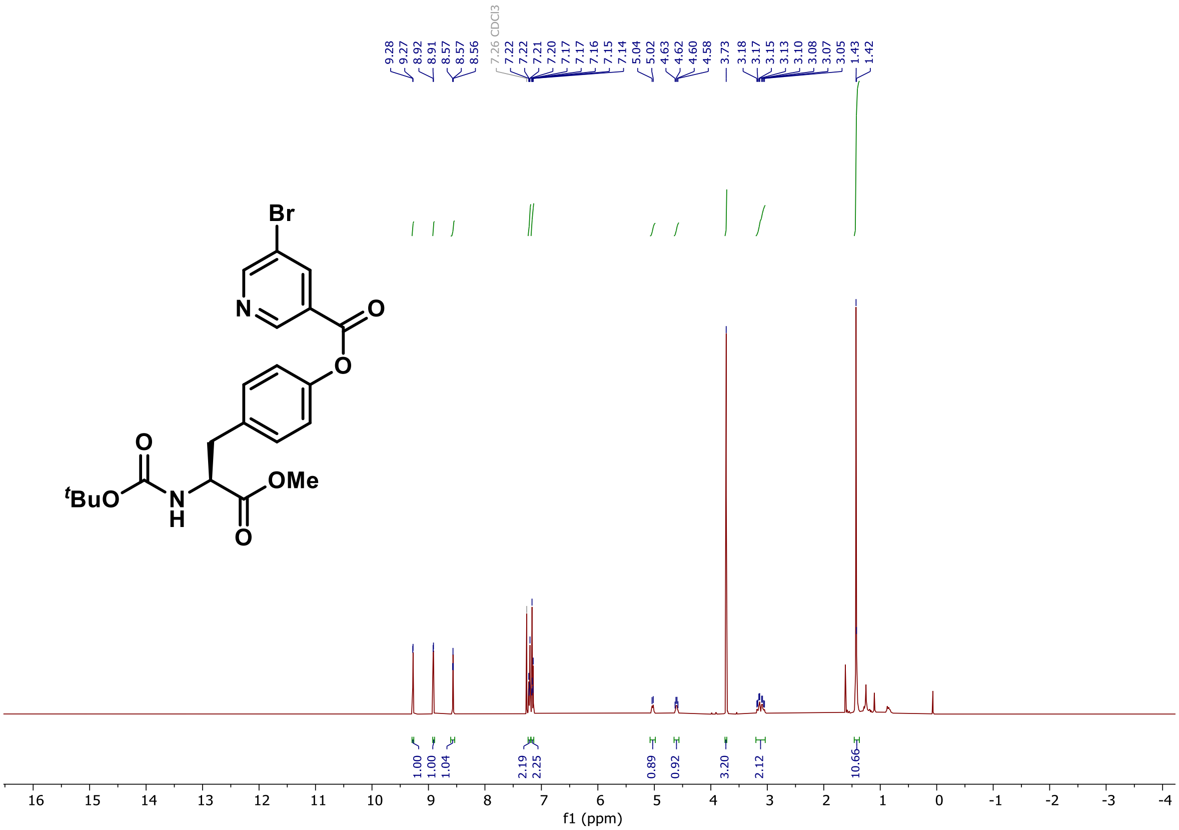

^{13}C NMR of SM2 ($CDCl_3$, 101 MHz)

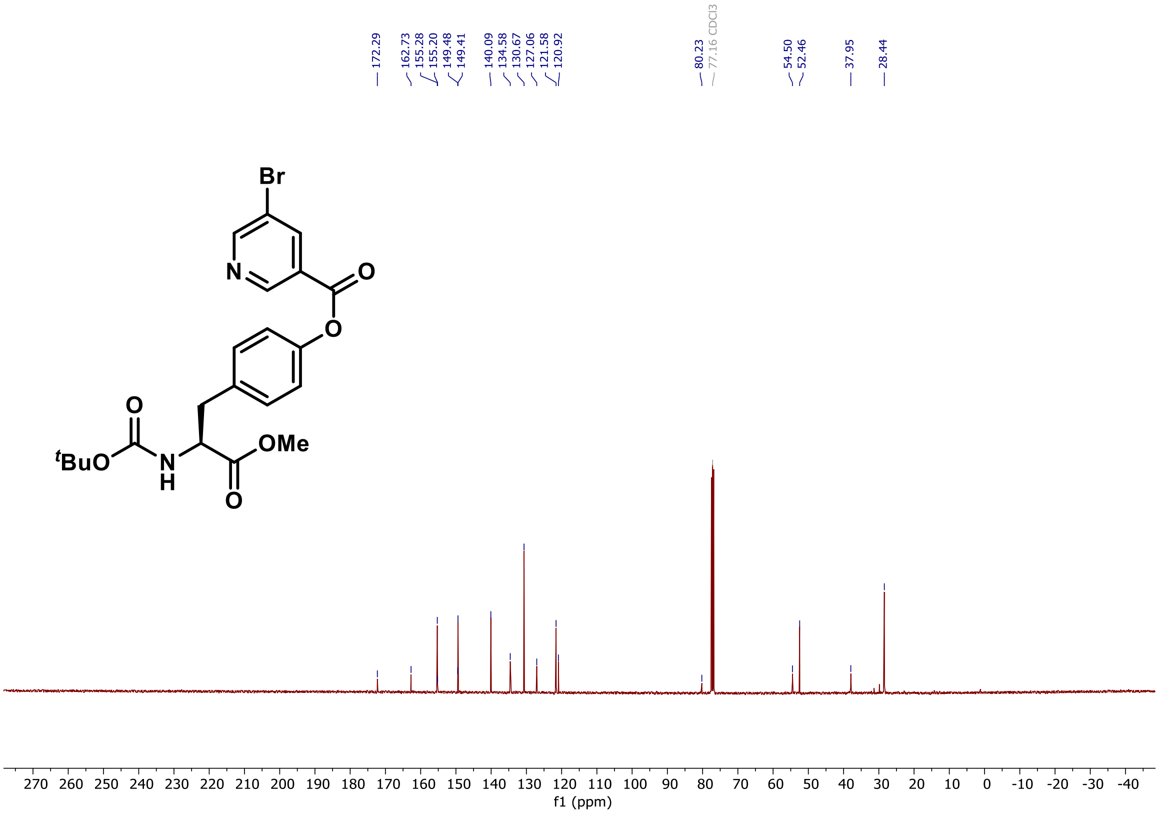

^{1}H NMR of 3 ($CDCl_3$, 400 MHz)

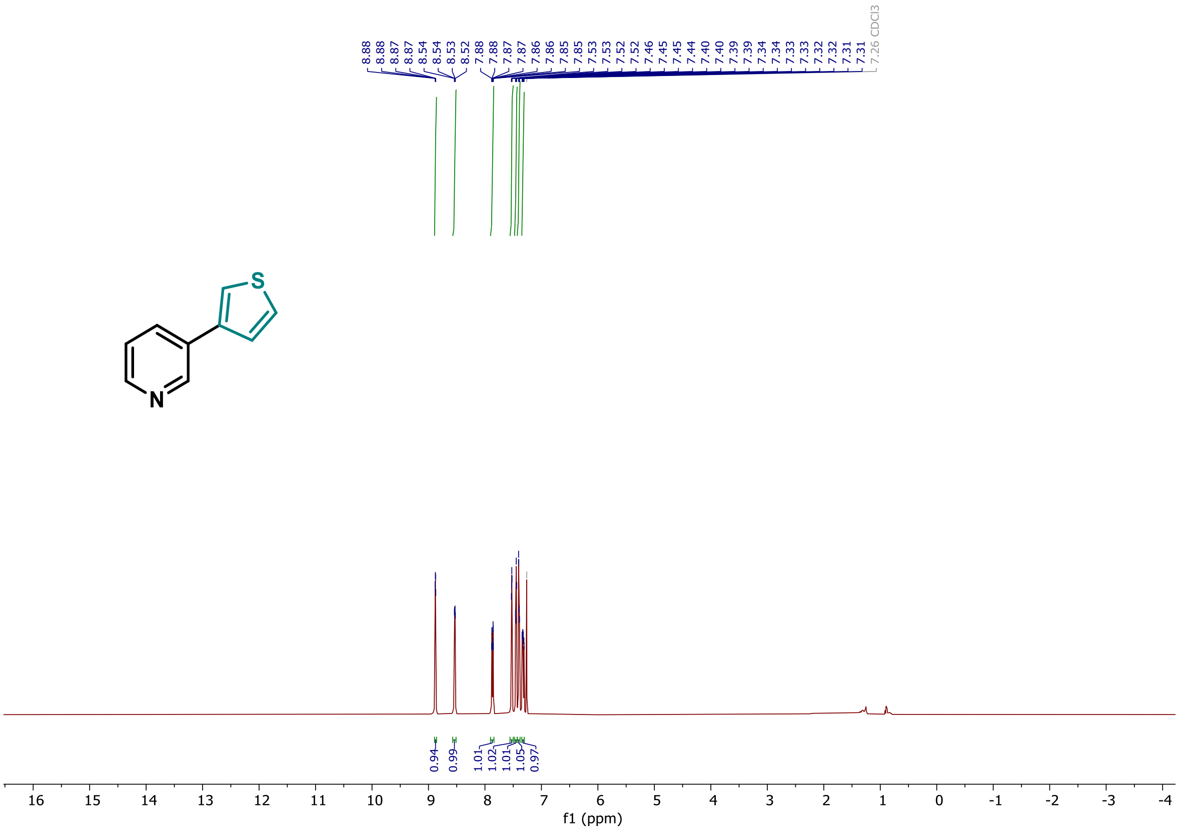

^{13}C NMR of 3 ($CDCl_3$, 101 MHz)

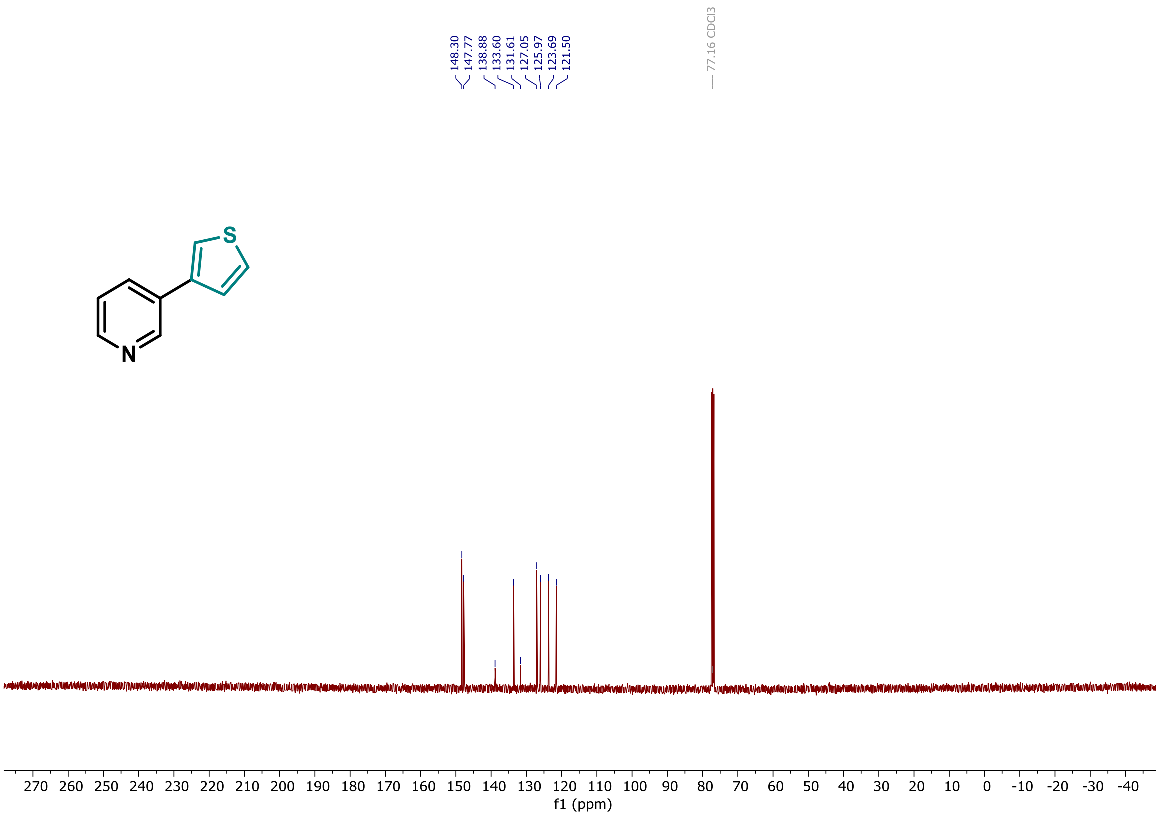

^{1}H NMR of 4 ($CDCl_3$, 400 MHz)

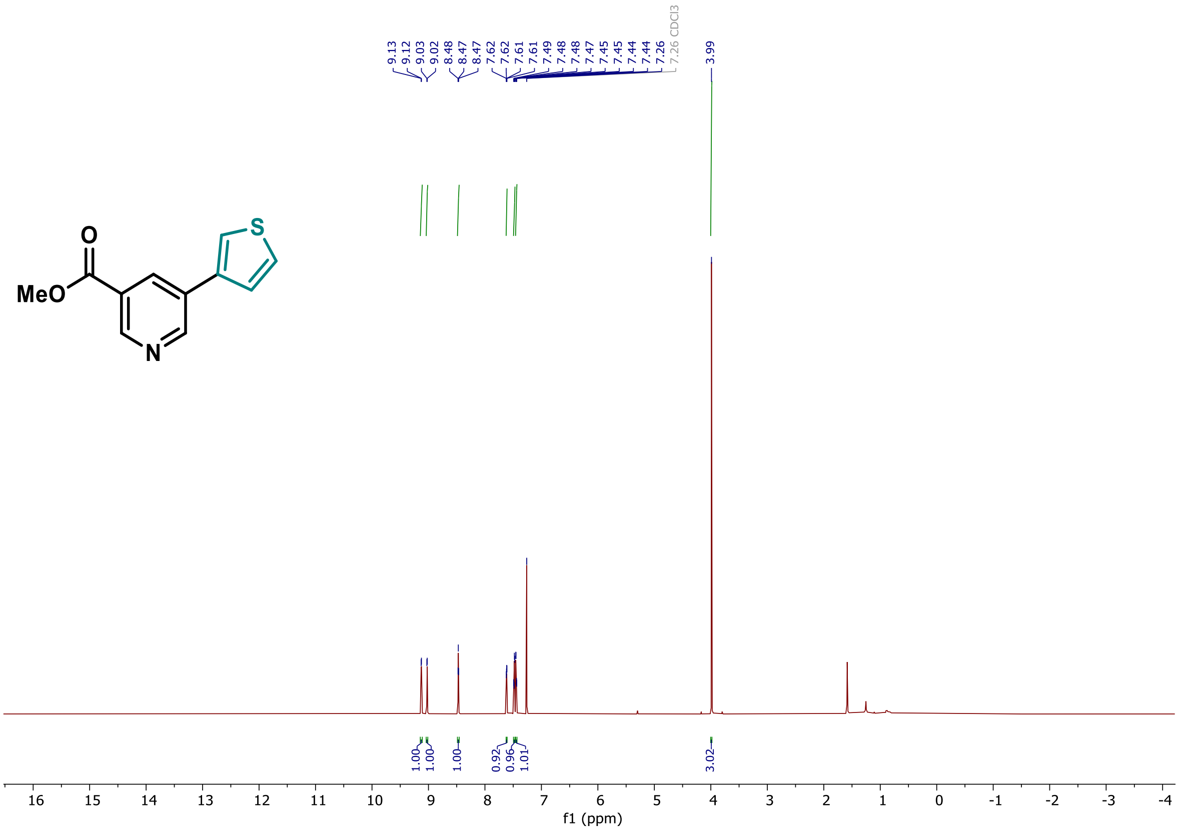

^{13}C NMR of 4 ($CDCl_3$, 101 MHz)

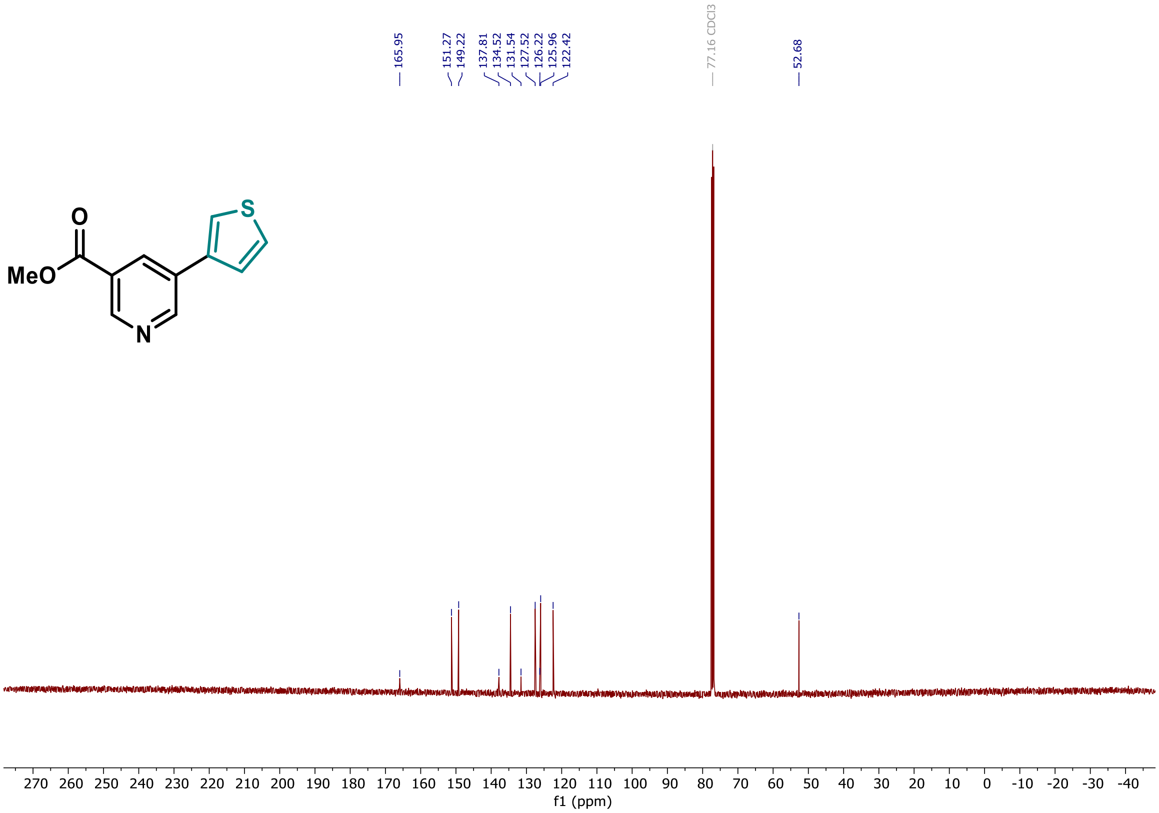

^{1}H NMR of 5 ($CDCl_3$, 400 MHz)

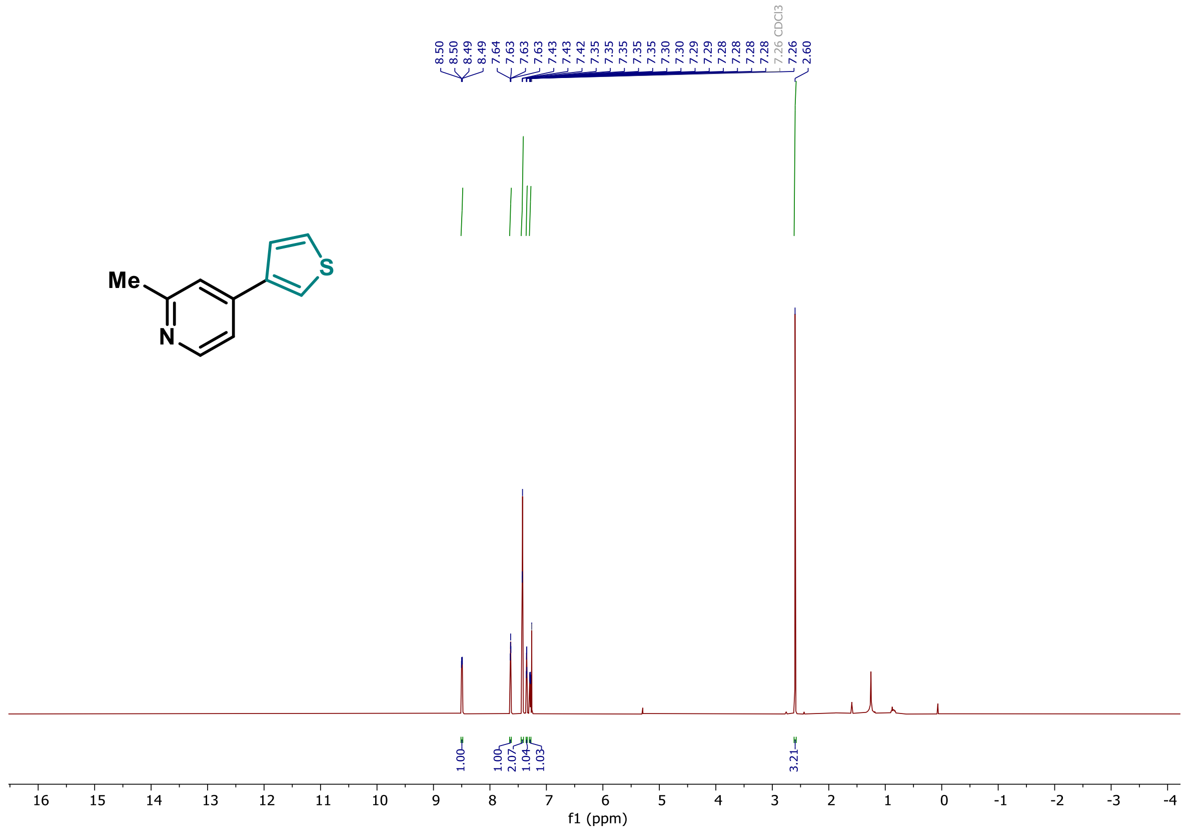

^{13}C NMR of 5 ($CDCl_3$, 101 MHz)

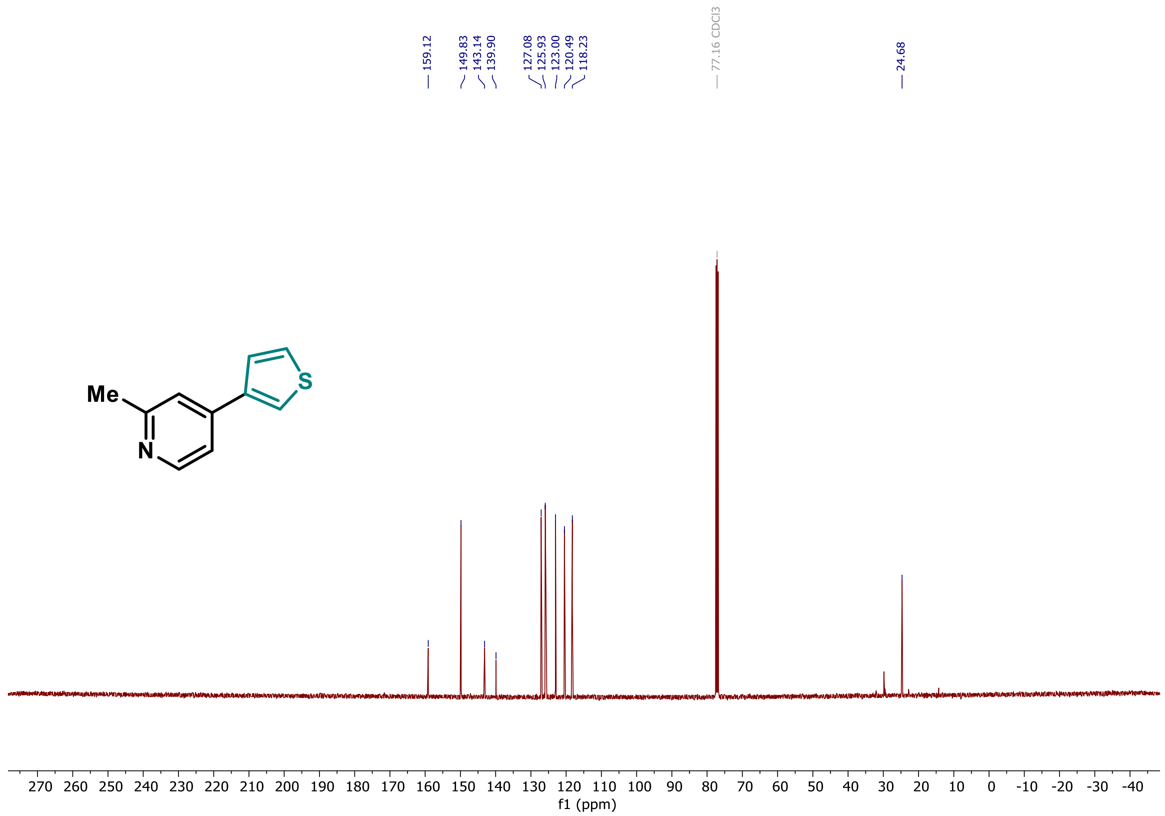

^{1}H NMR of 6 ($CDCl_3$, 400 MHz)

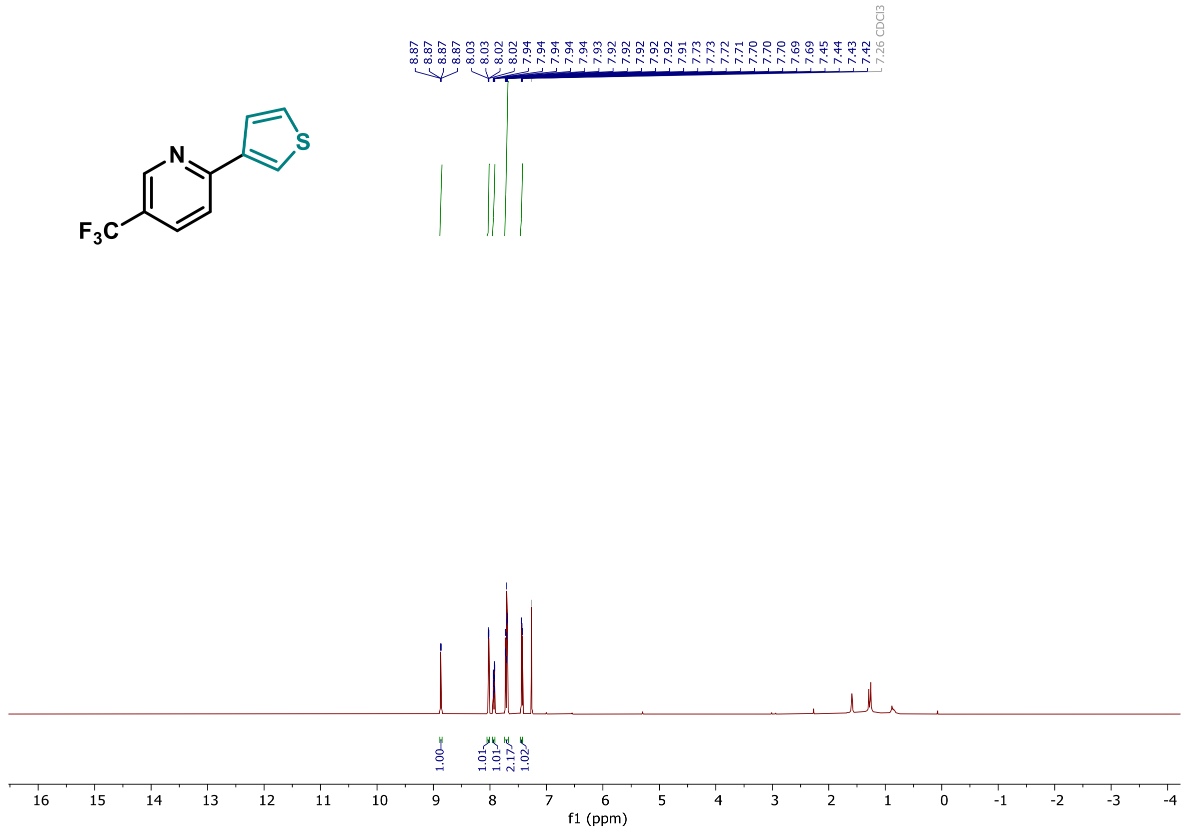

^{13}C NMR of 6 ($CDCl_3$, 101 MHz)

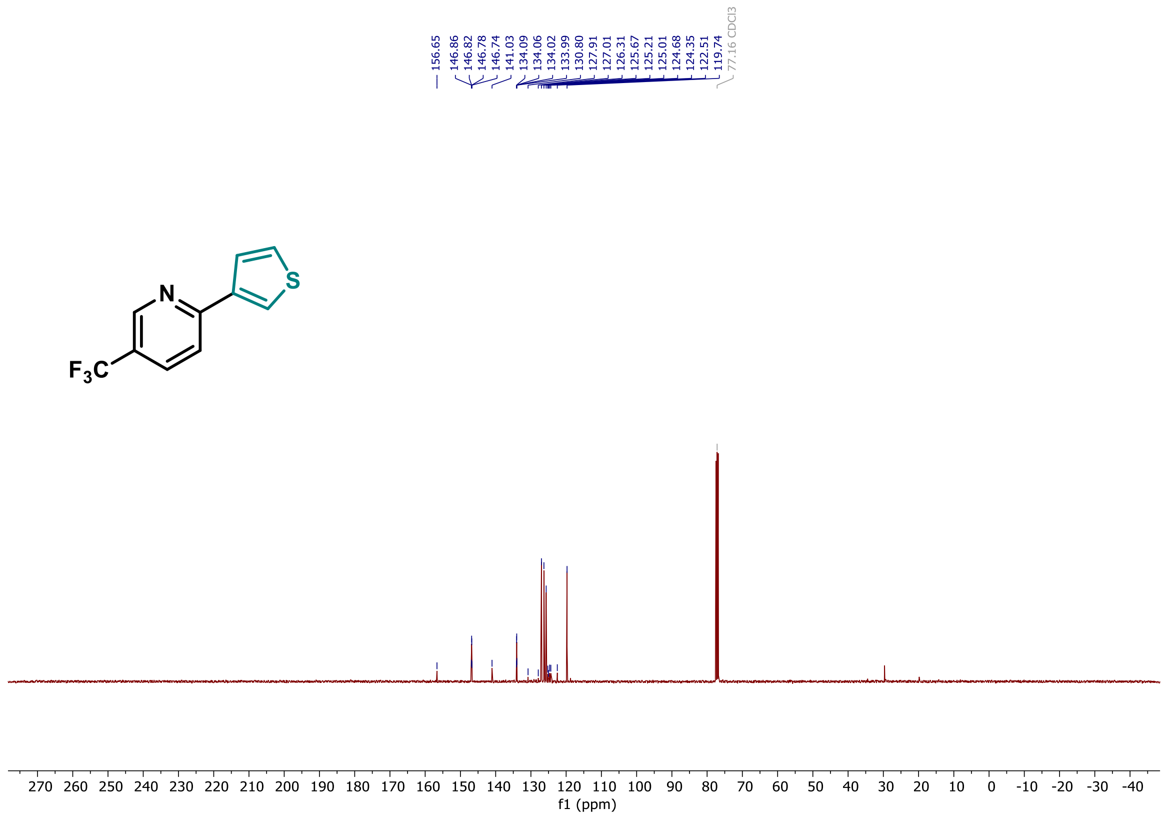

^{19}F NMR of 6 ($CDCl_3$, 282 MHz)

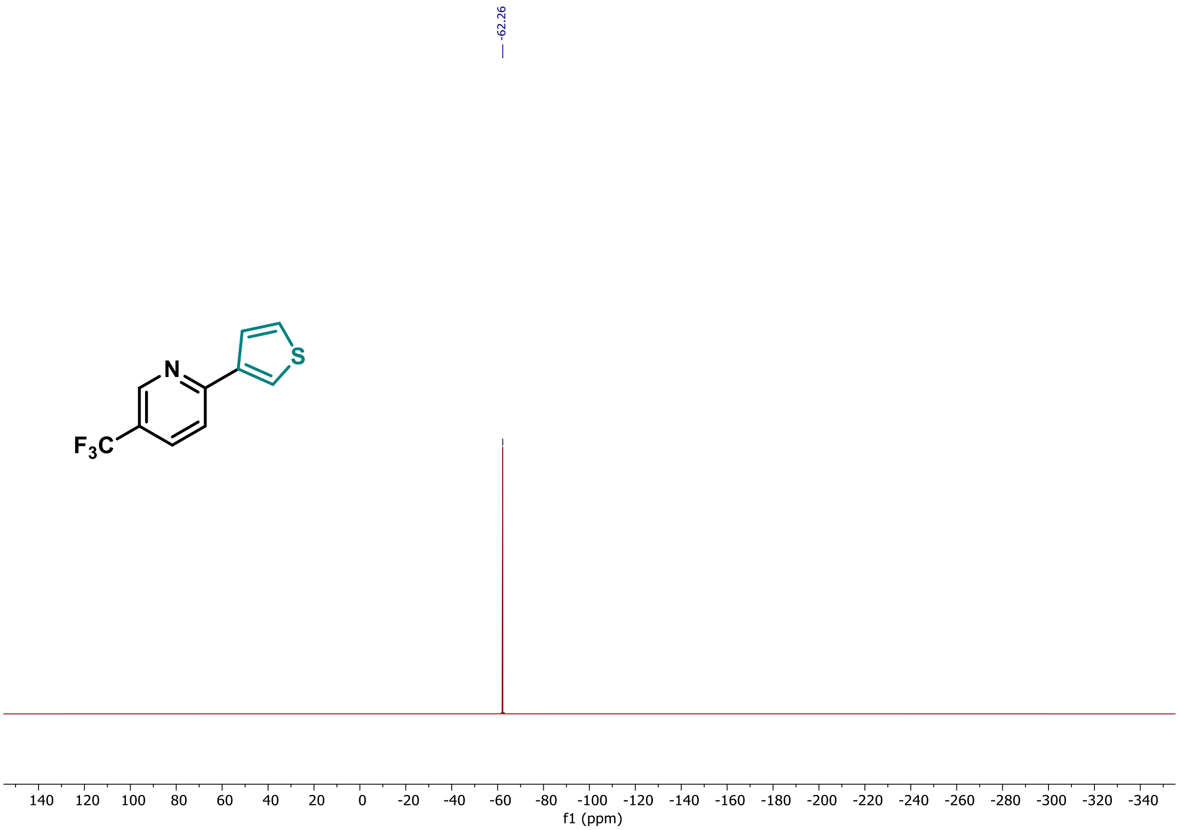

^{1}H NMR of 7 ($CDCl_3$, 400 MHz)

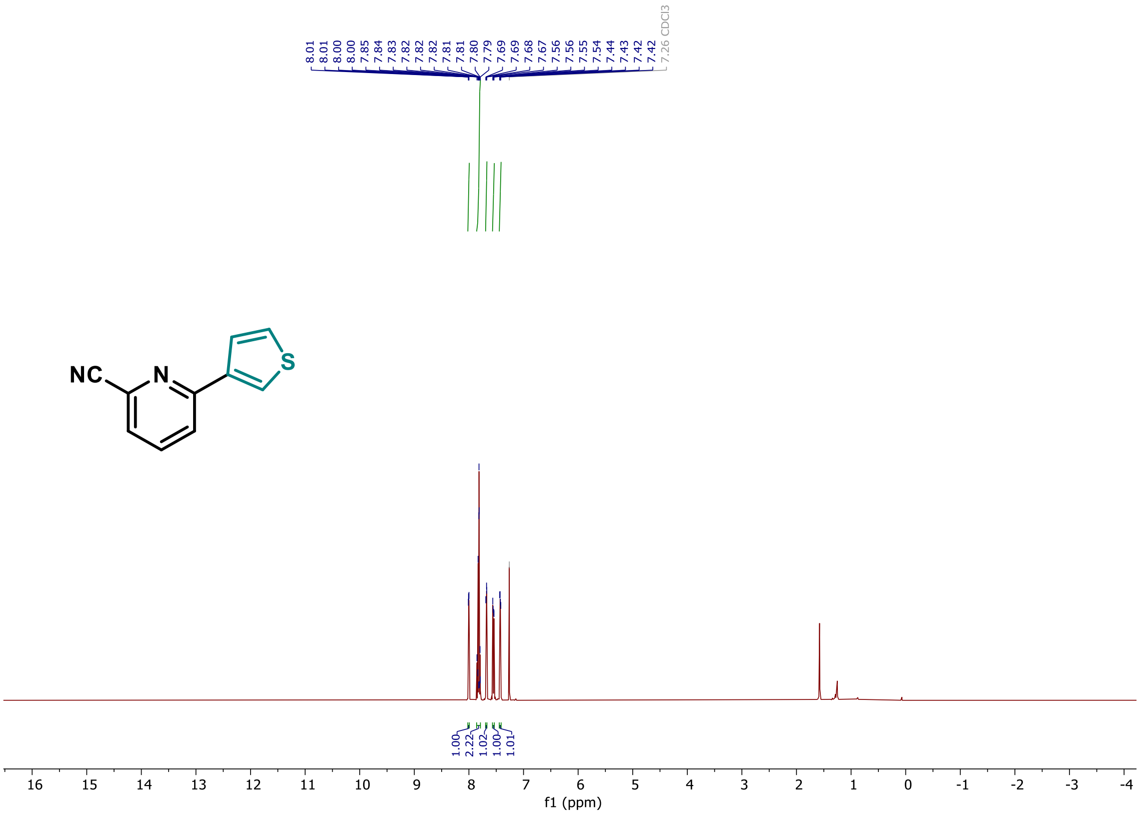

^{13}C NMR of 7 ($CDCl_3$, 101 MHz)

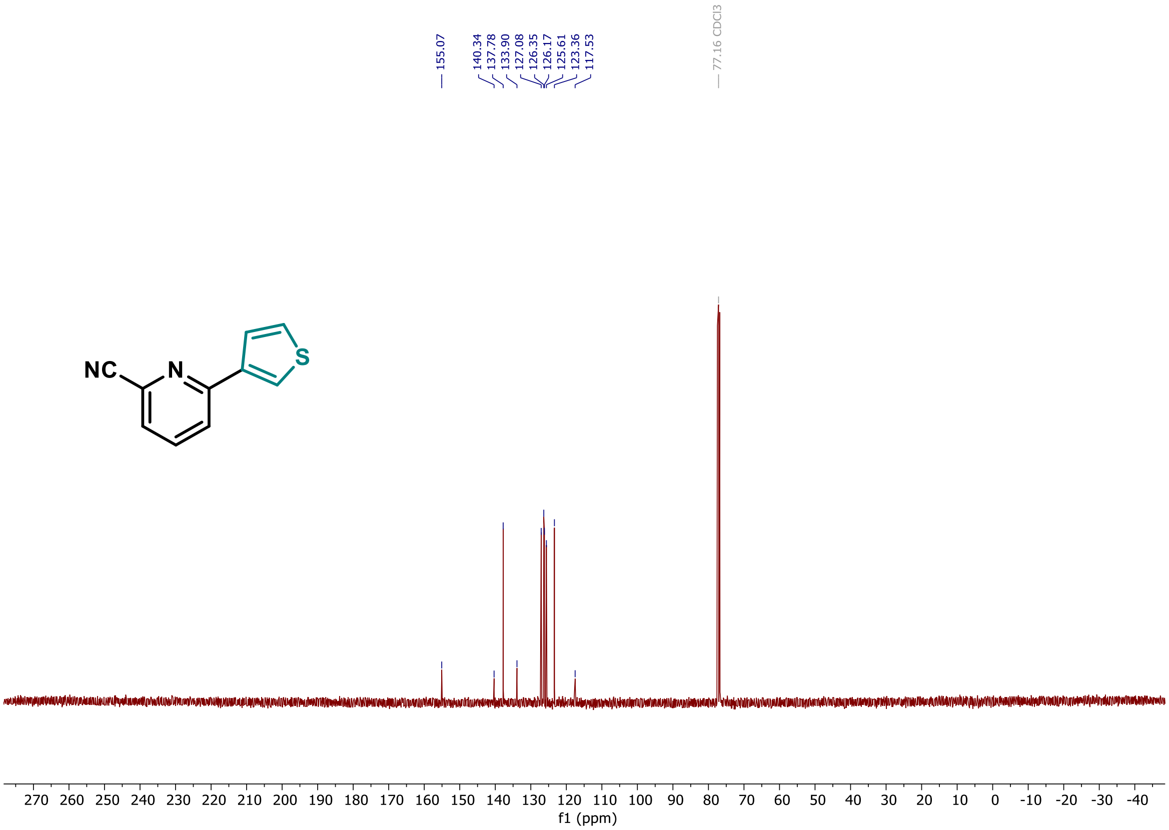

^{1}H NMR of 8 ($CDCl_3$, 400 MHz)

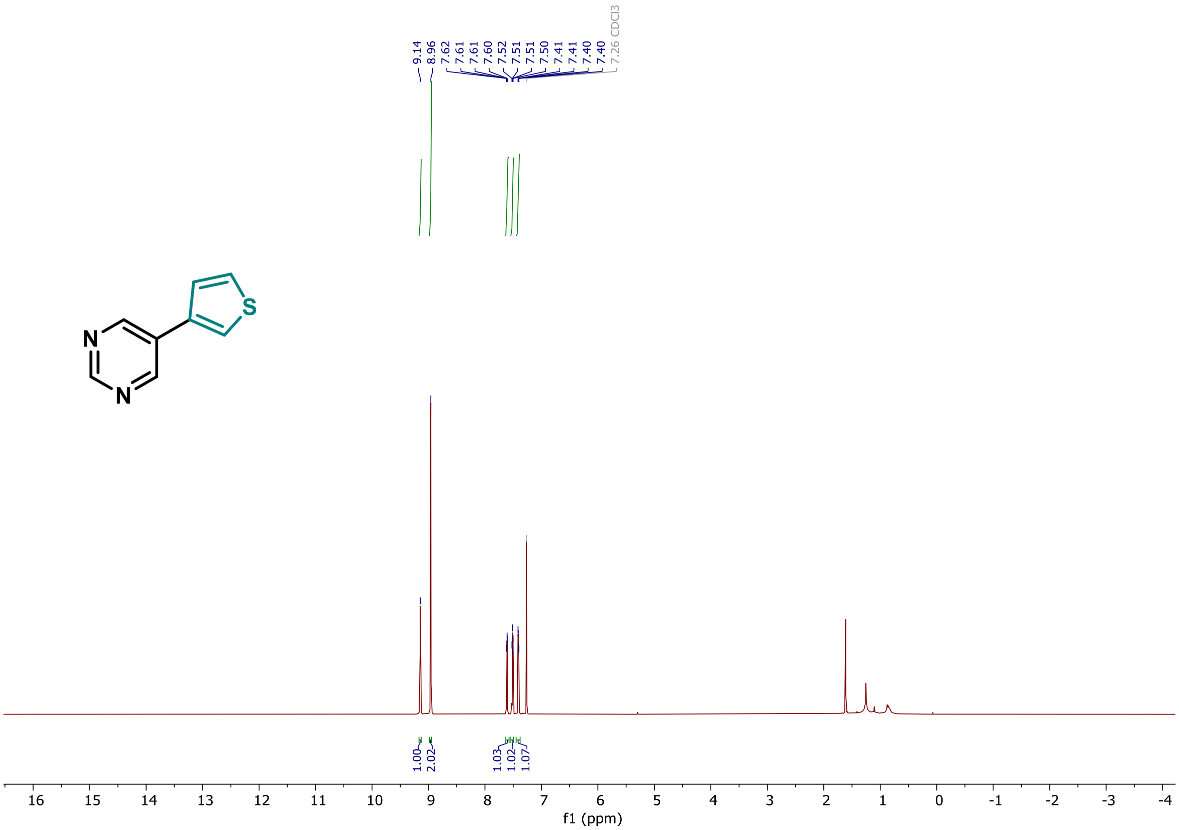

^{13}C NMR of 8 ($CDCl_3$, 101 MHz)

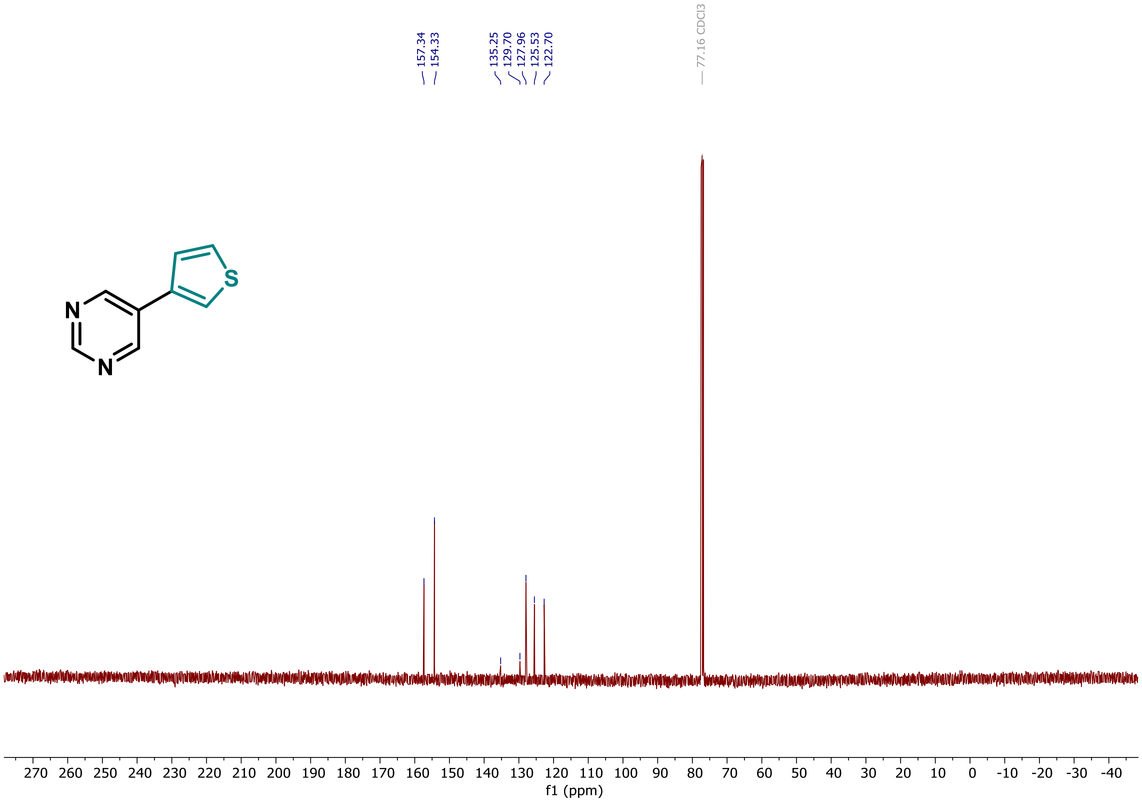

^{1}H NMR of 9 ($CDCl_3$, 400 MHz)

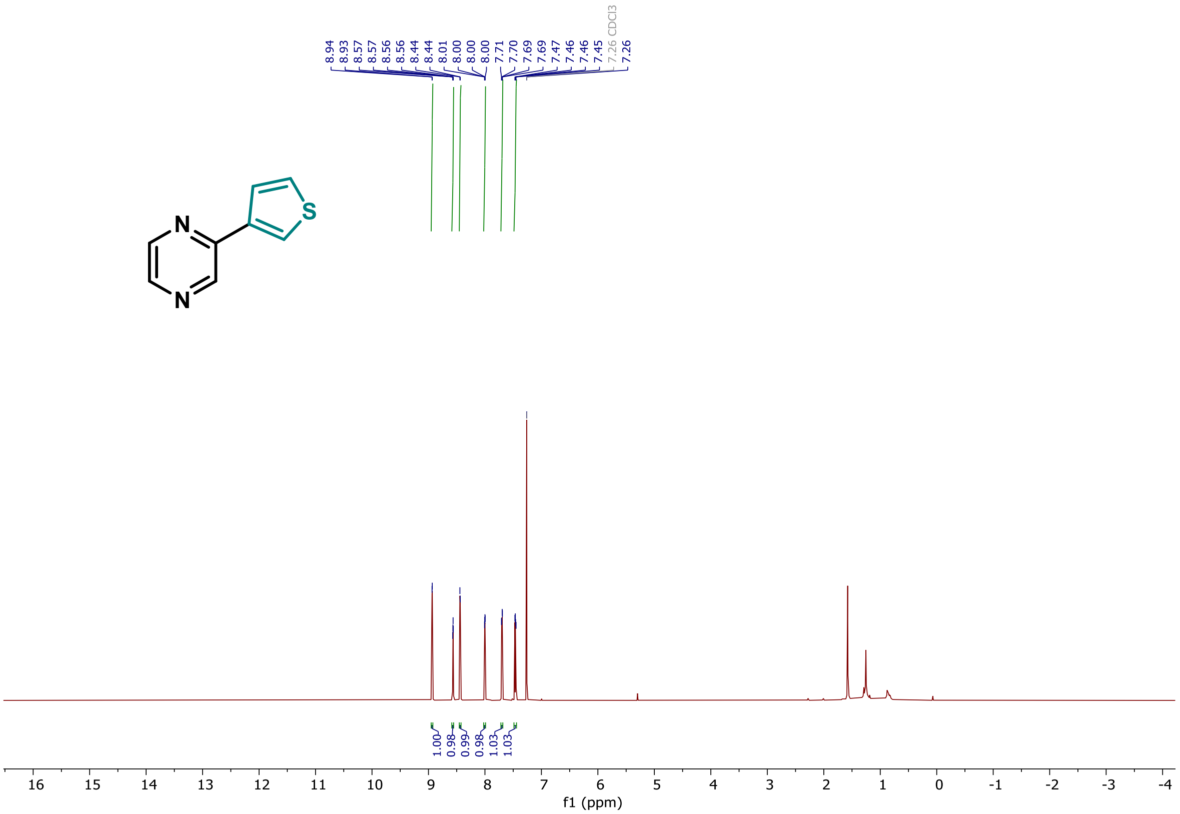

^{13}C NMR of 9 ($CDCl_3$, 101 MHz)

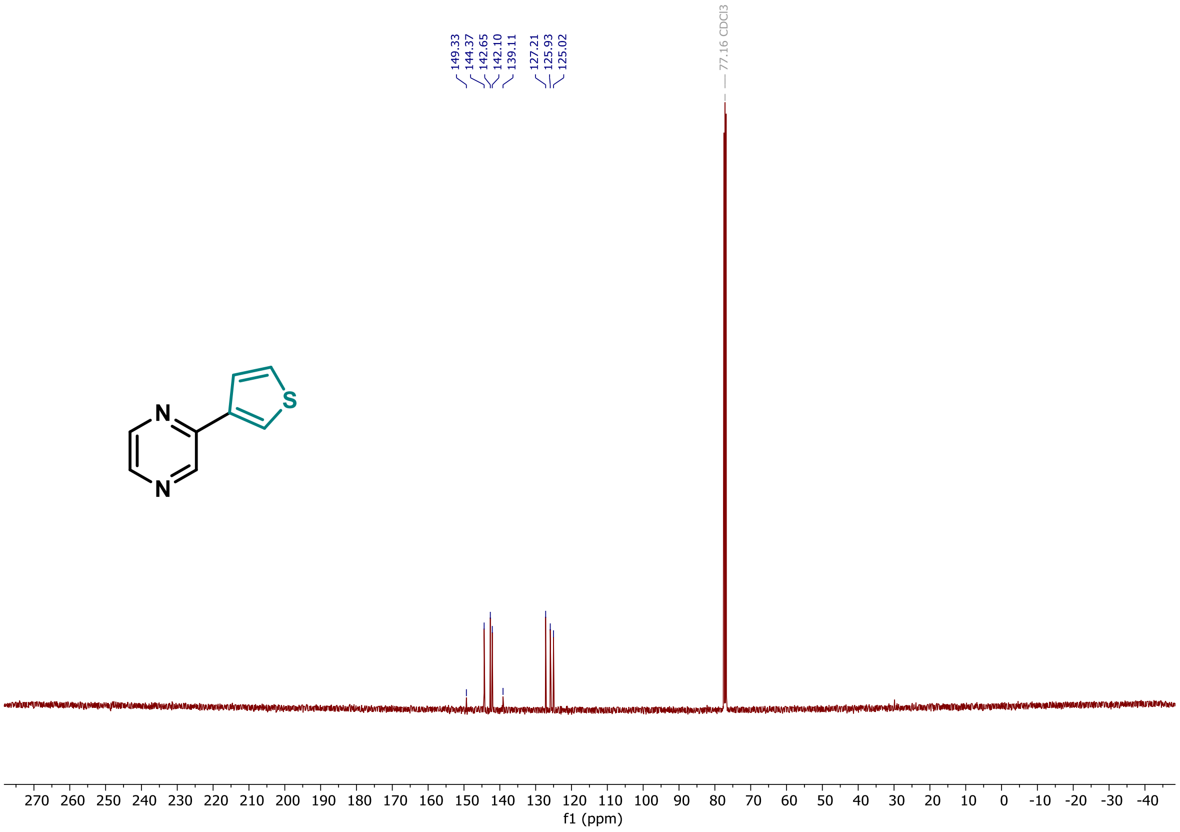

^{1}H NMR of 10 ($CDCl_3$, 400 MHz)

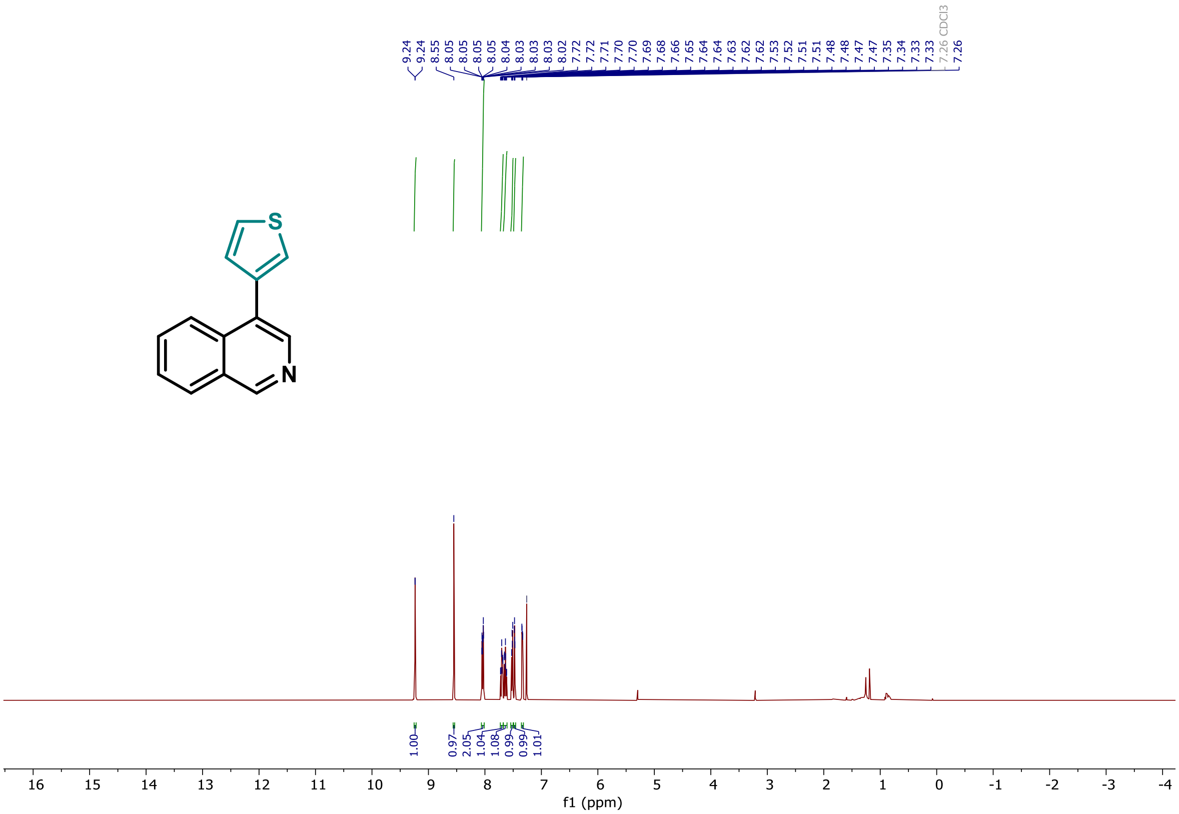

^{13}C NMR of 10 ($CDCl_3$, 101 MHz)

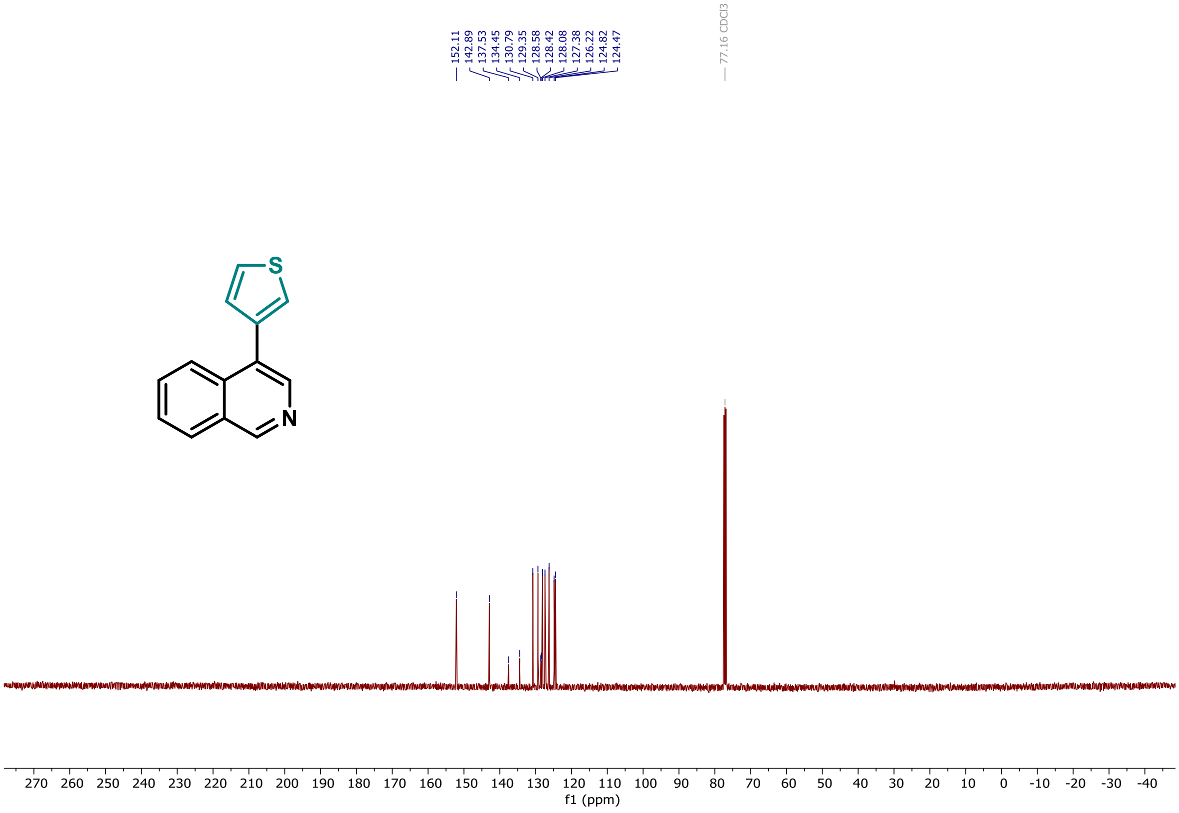

^{1}H NMR of 11 ($CDCl_3$, 400 MHz)

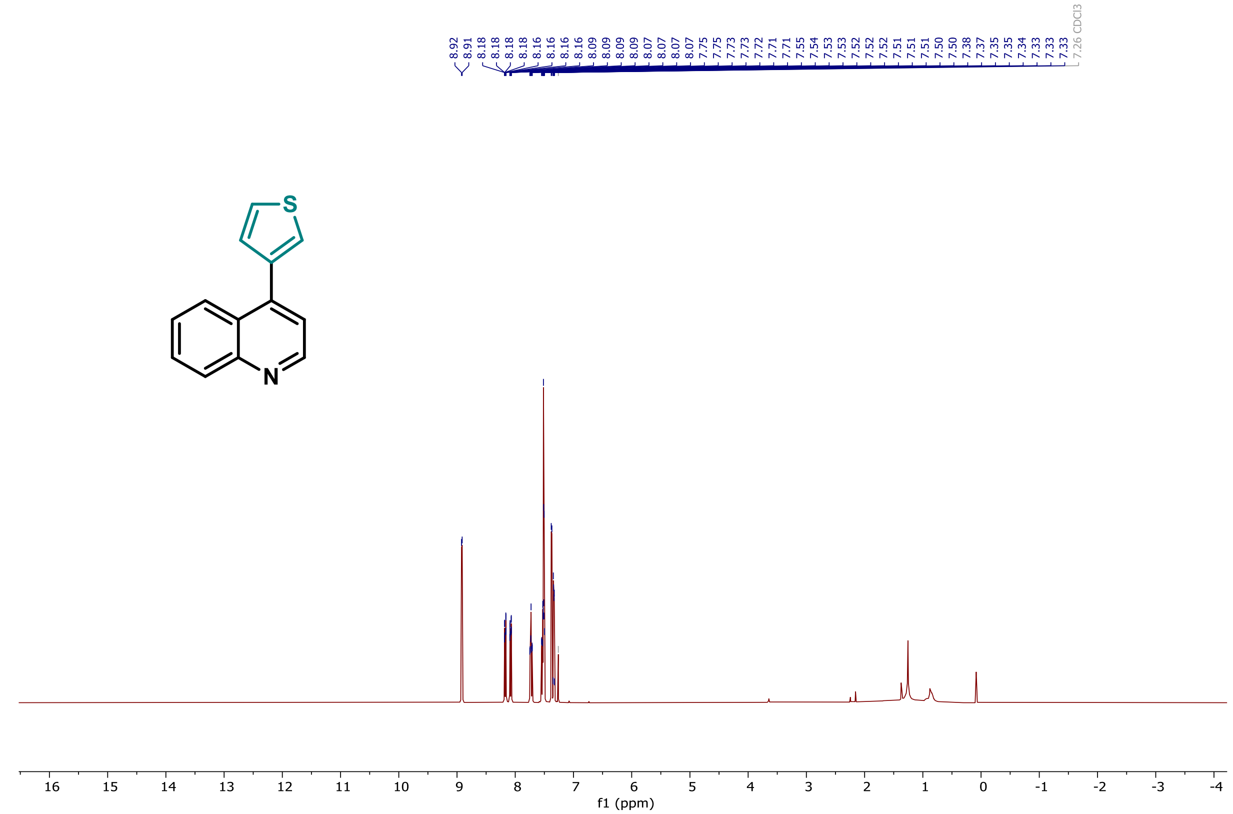

^{13}C NMR of 11 ($CDCl_3$, 75 MHz)

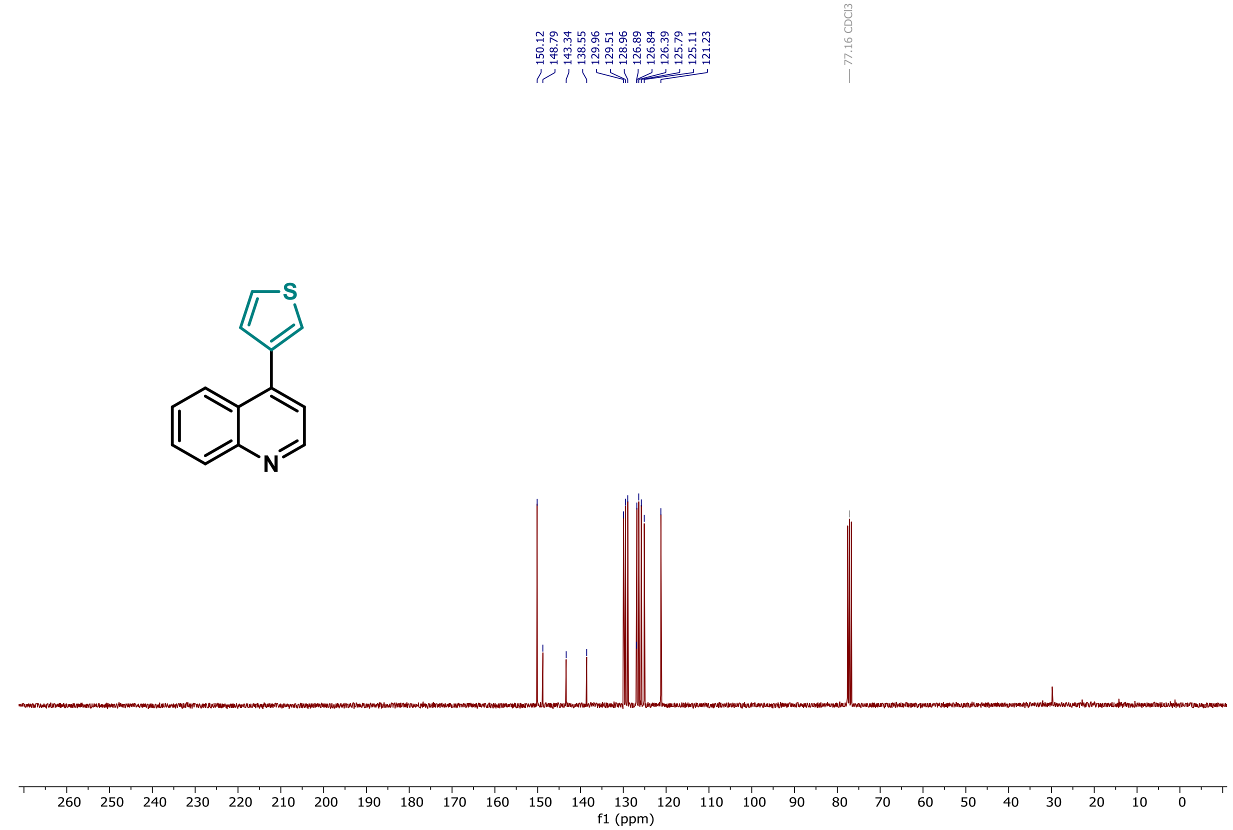

^{1}H NMR of 12 ($CDCl_3$, 400 MHz)

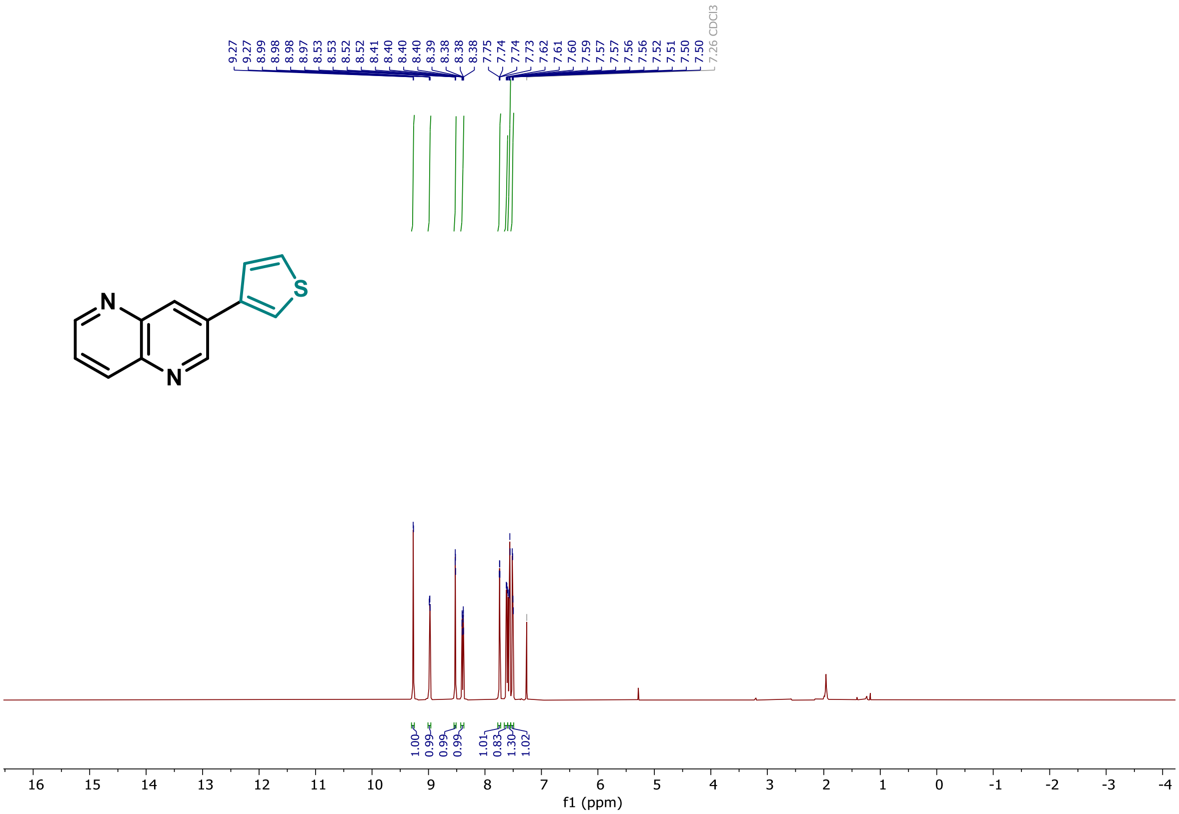

^{13}C NMR of 12 ($CDCl_3$, 101 MHz)

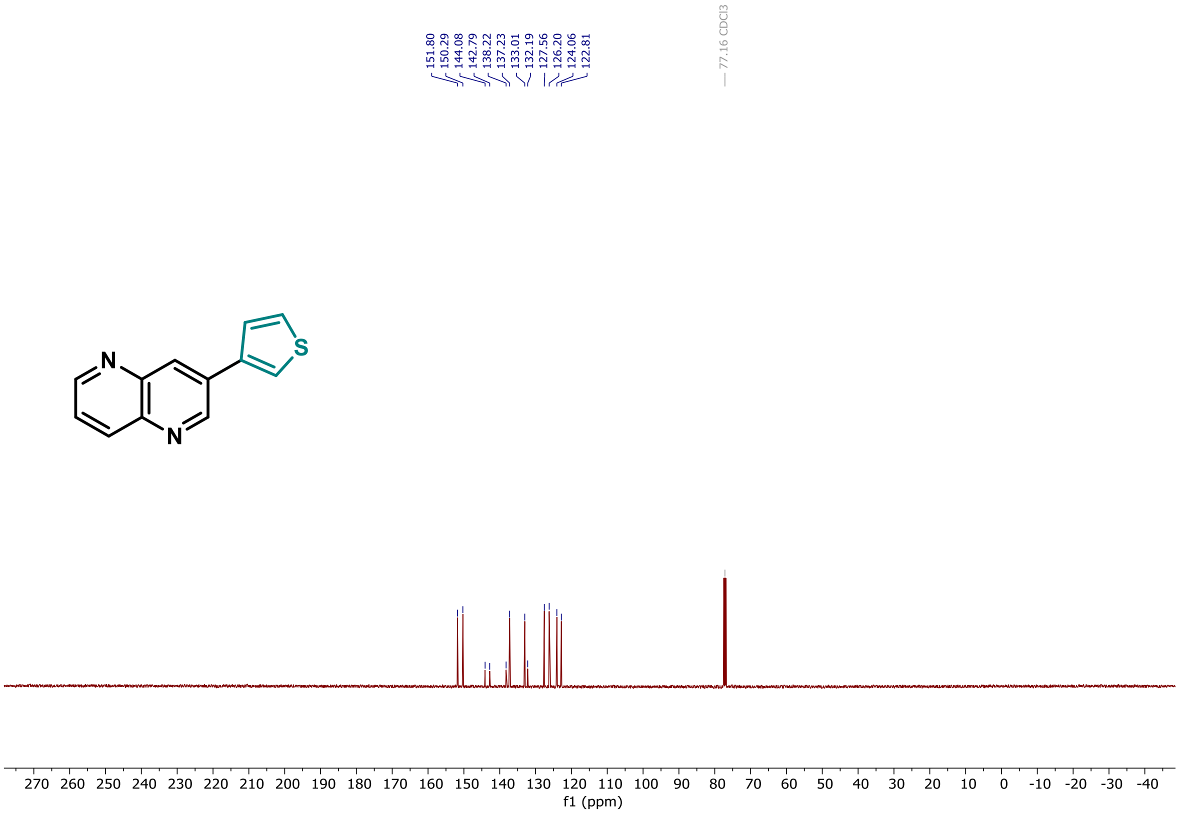

^{1}H NMR of 13 ($CDCl_3$, 400 MHz)

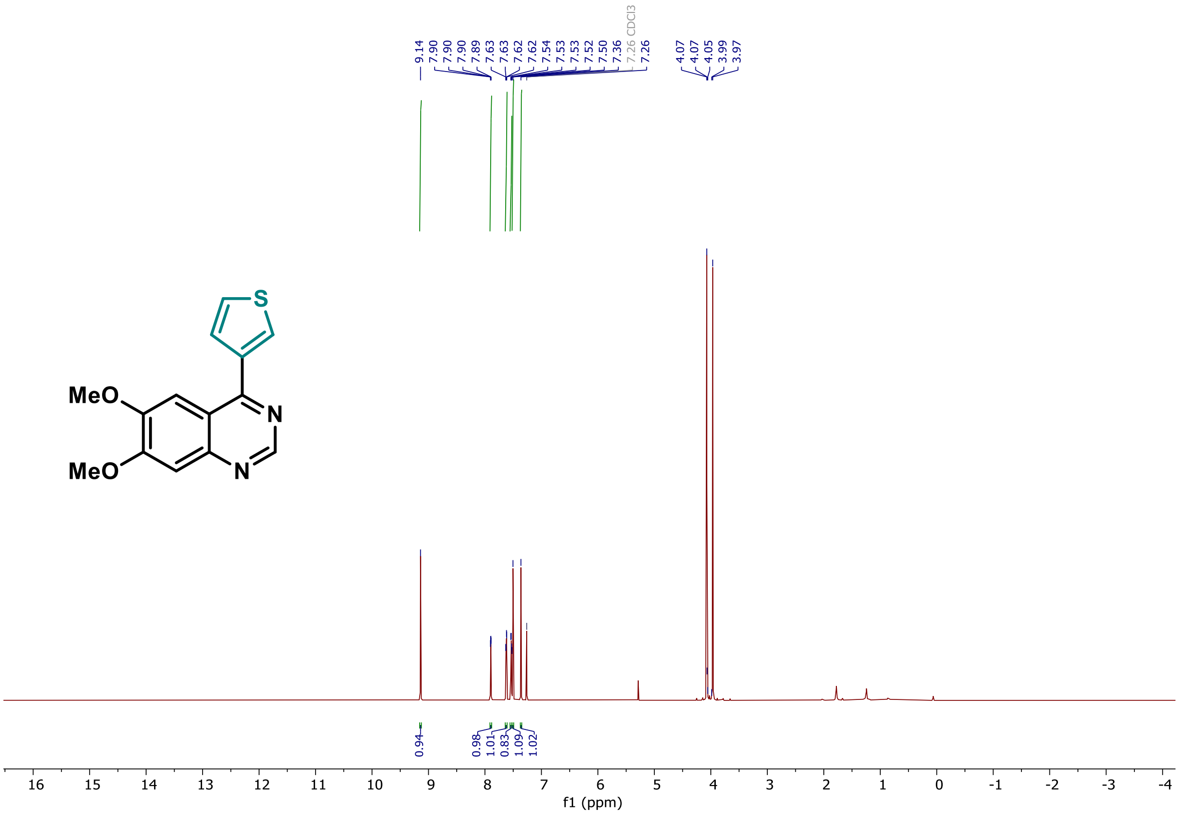

^{13}C NMR of 13 ($CDCl_3$, 101 MHz)

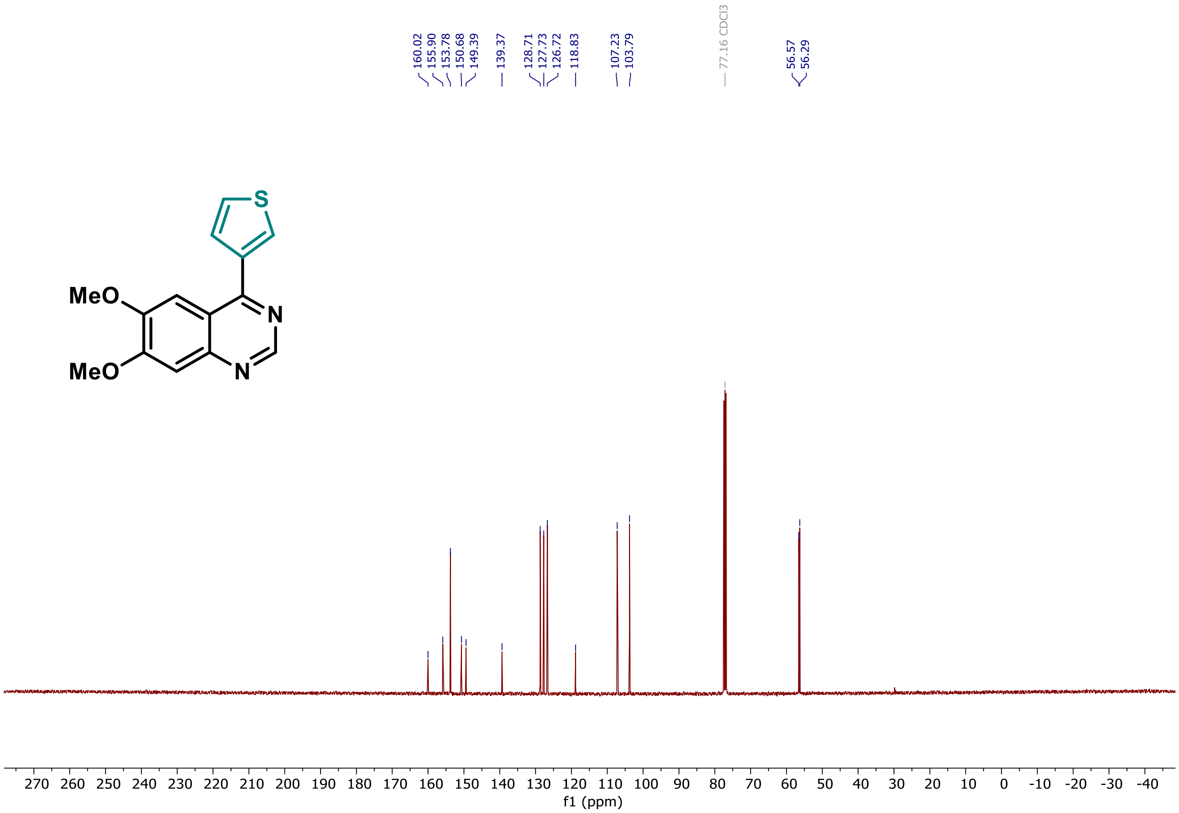

^{1}H NMR of 14 ($CDCl_3$, 400 MHz)

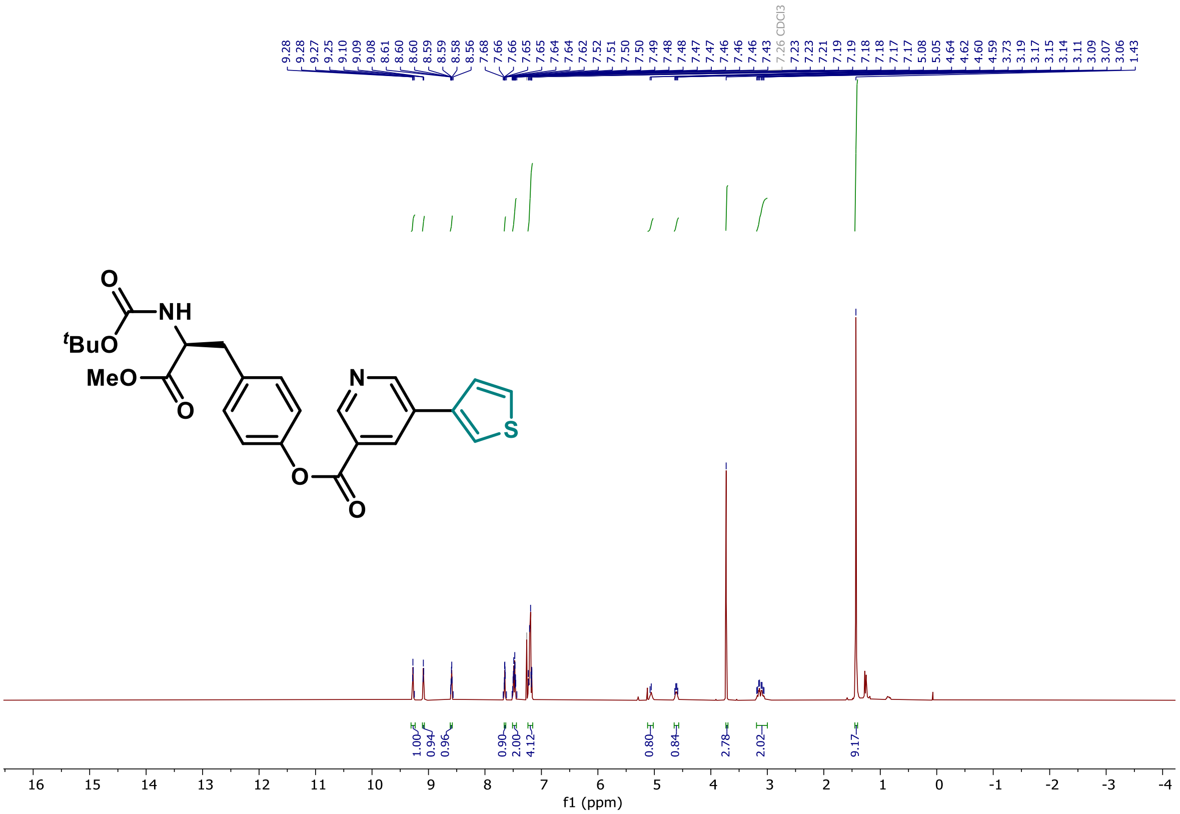

^{13}C NMR of 14 ($CDCl_3$, 101 MHz)

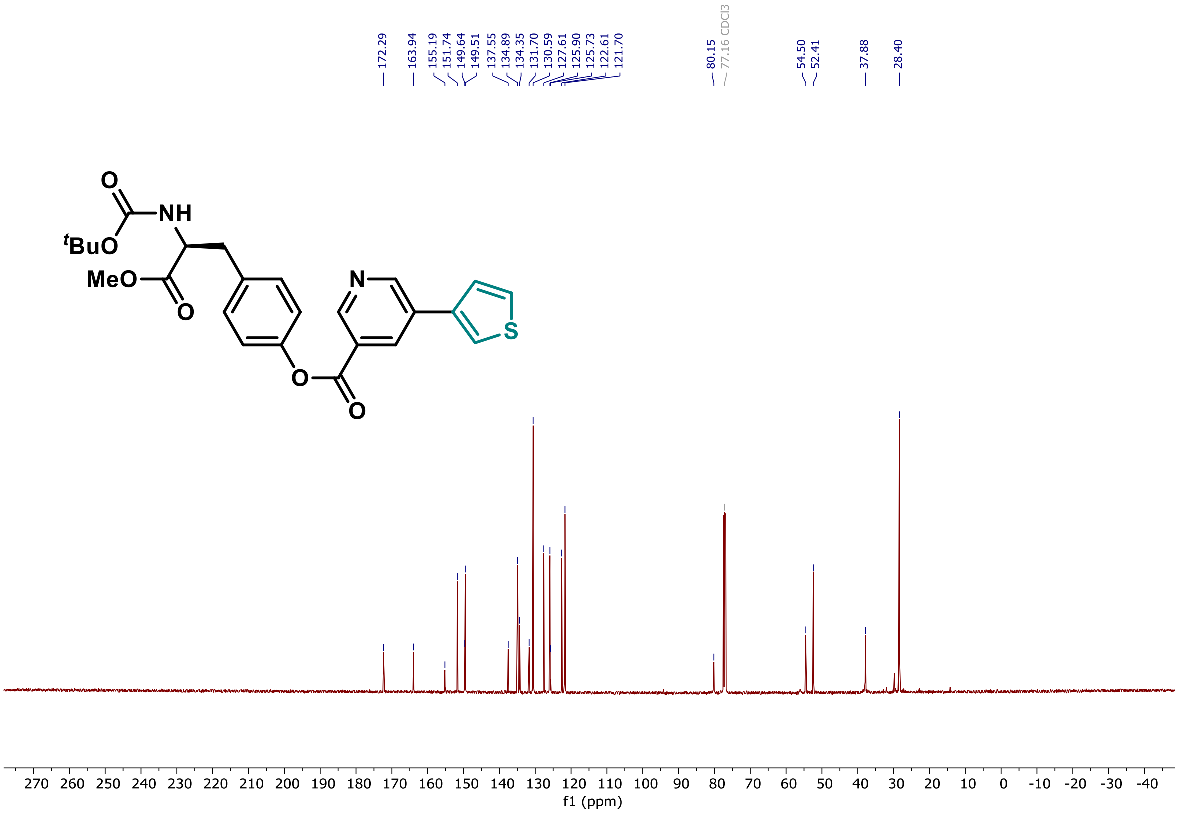

^{1}H NMR of 15 ($CDCl_3$, 400 MHz)

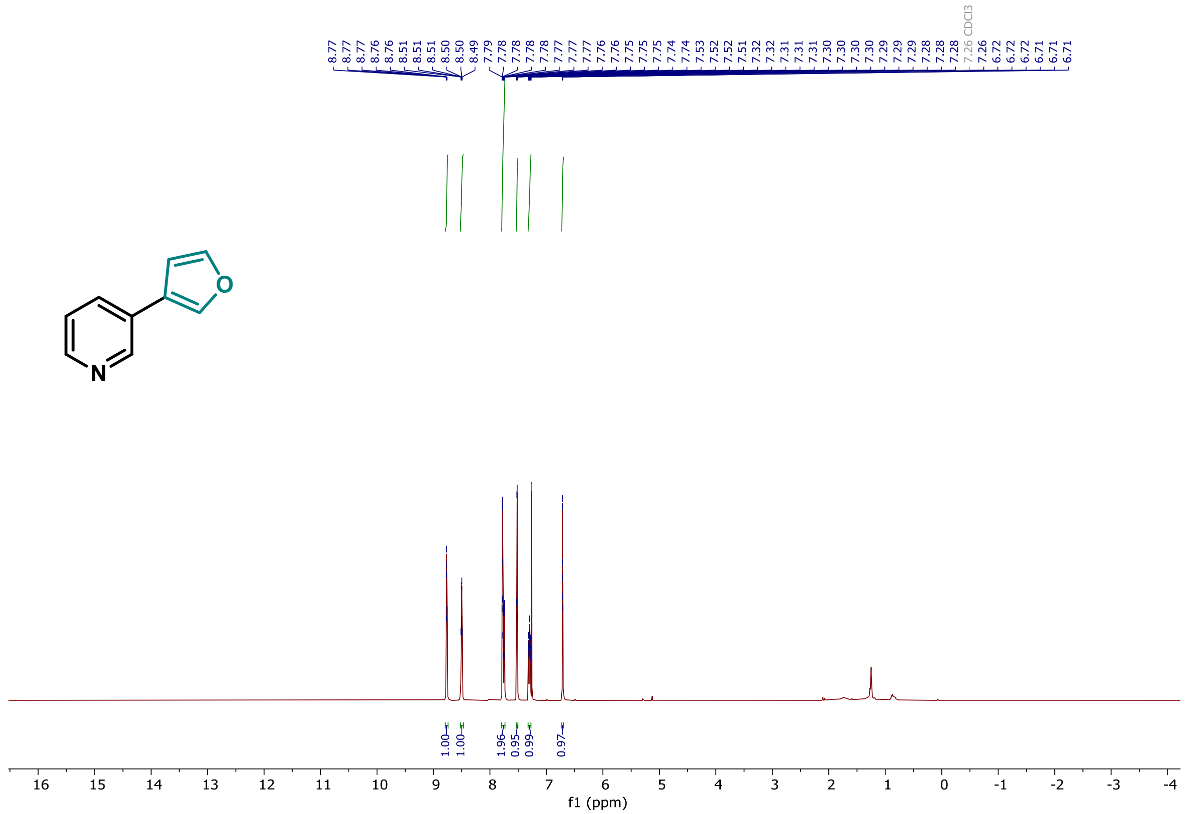

^{13}C NMR of 15 ($CDCl_3$, 101 MHz)

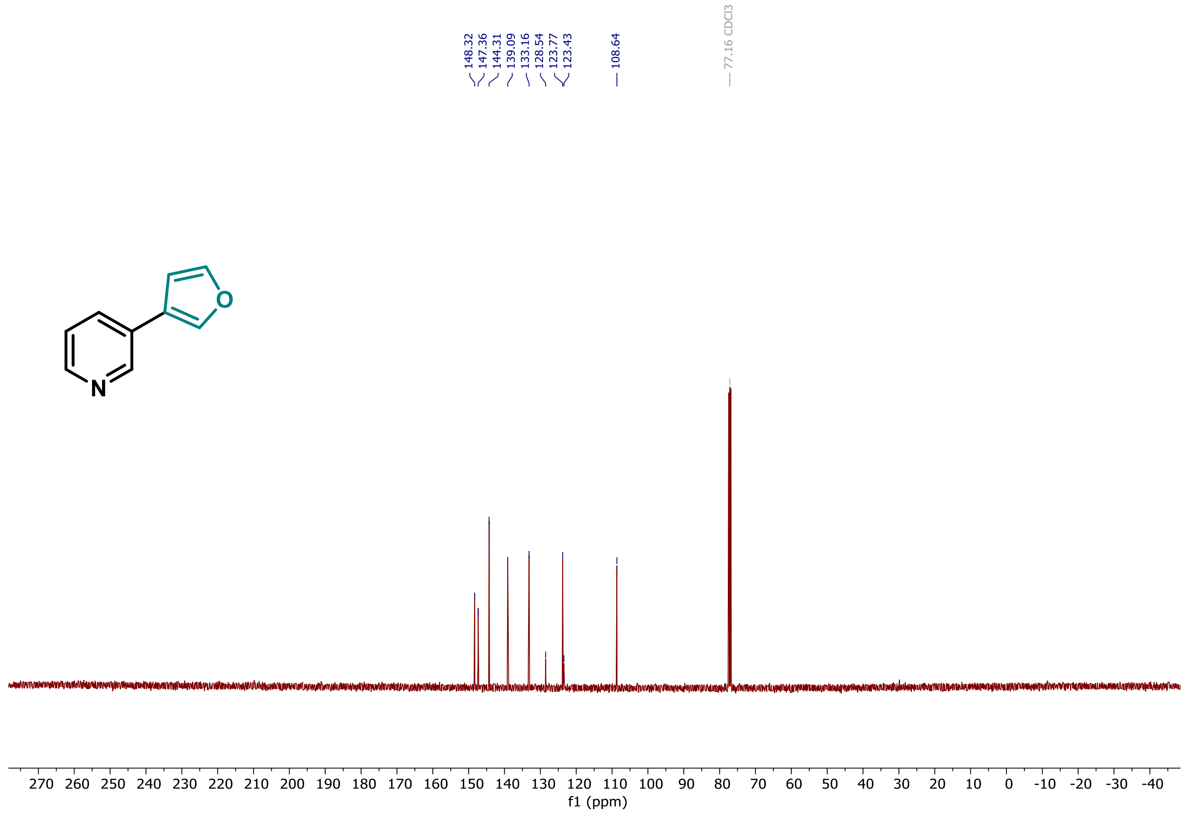

^{1}H NMR of 16 ($CDCl_3$, 400 MHz)

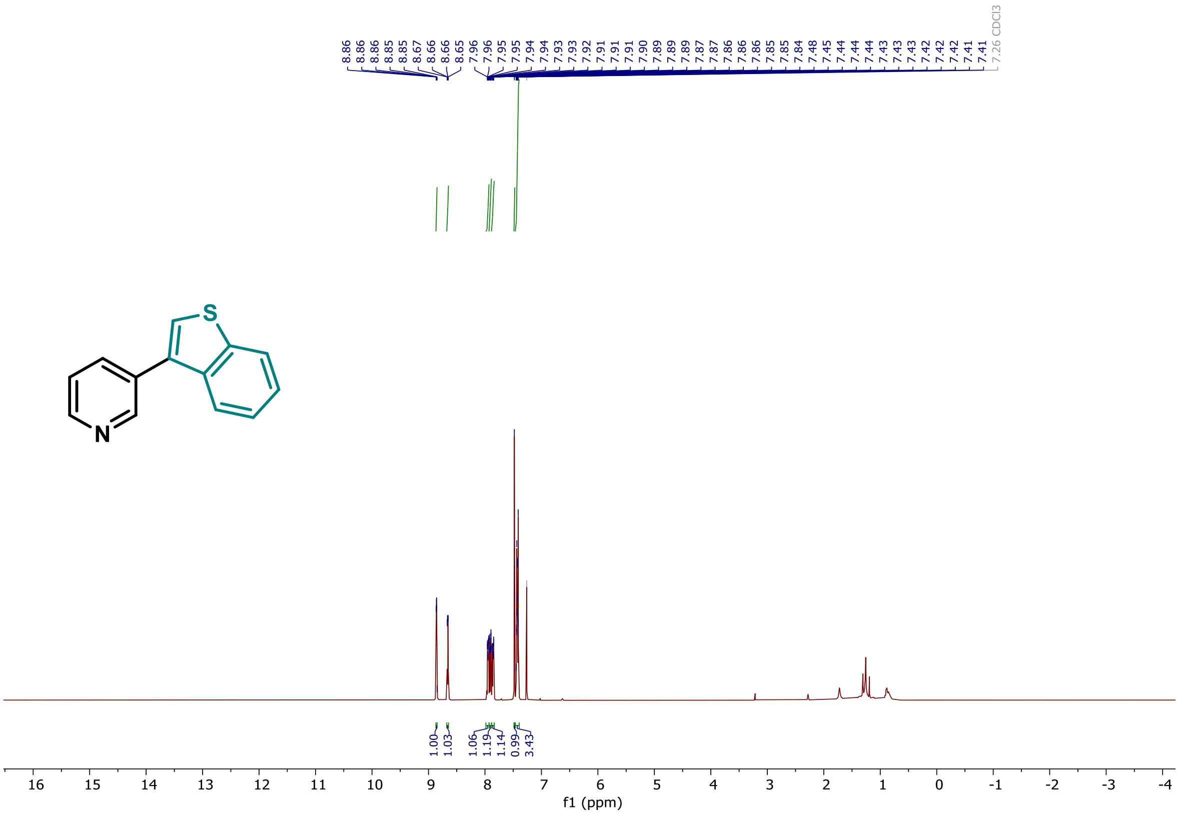

^{13}C NMR of 16 ($CDCl_3$, 101 MHz)

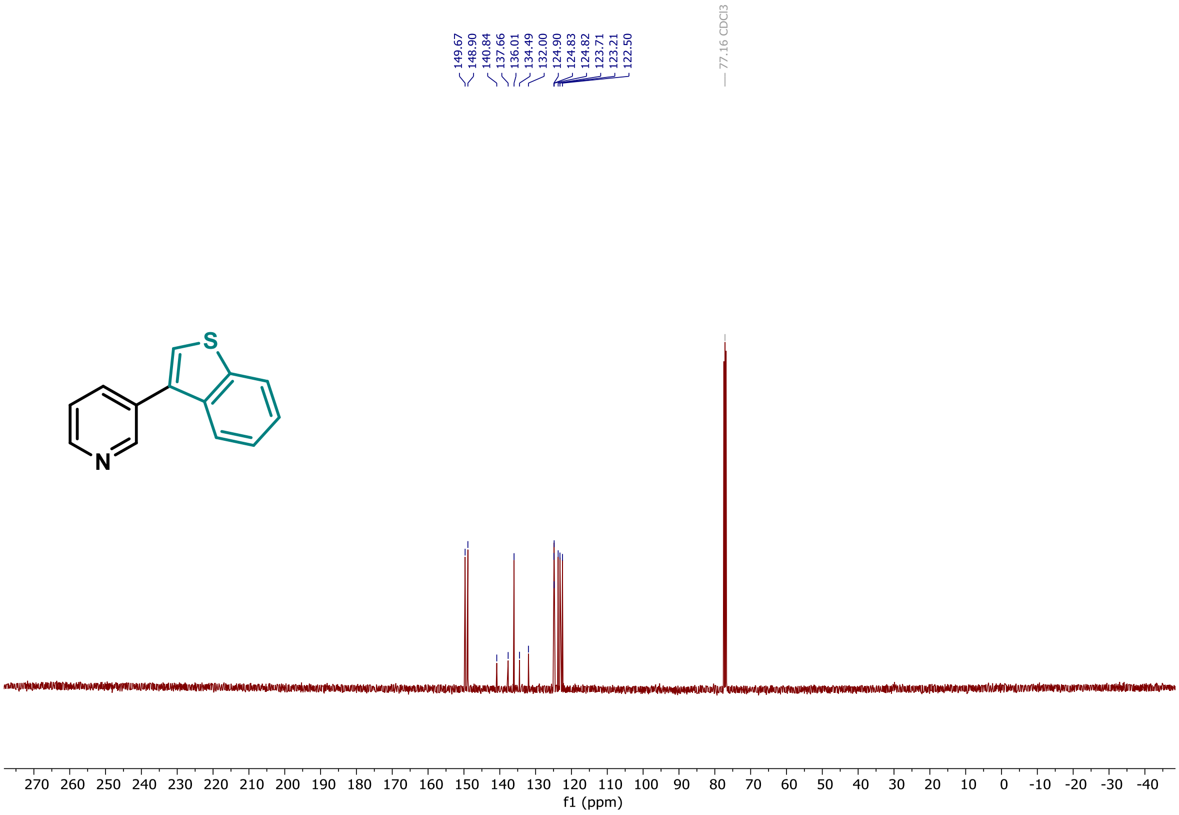

^{1}H NMR of 17 ($CDCl_3$, 400 MHz)

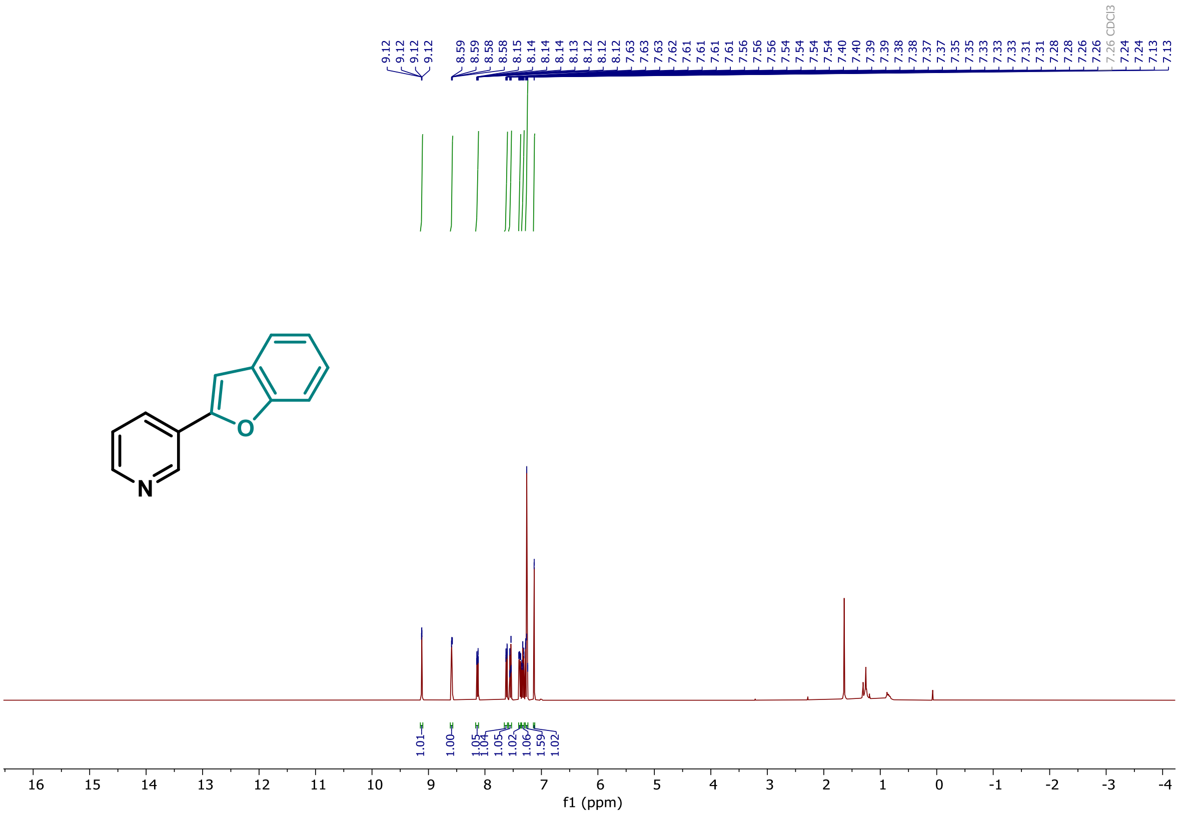

^{13}C NMR of 17 ($CDCl_3$, 101 MHz)

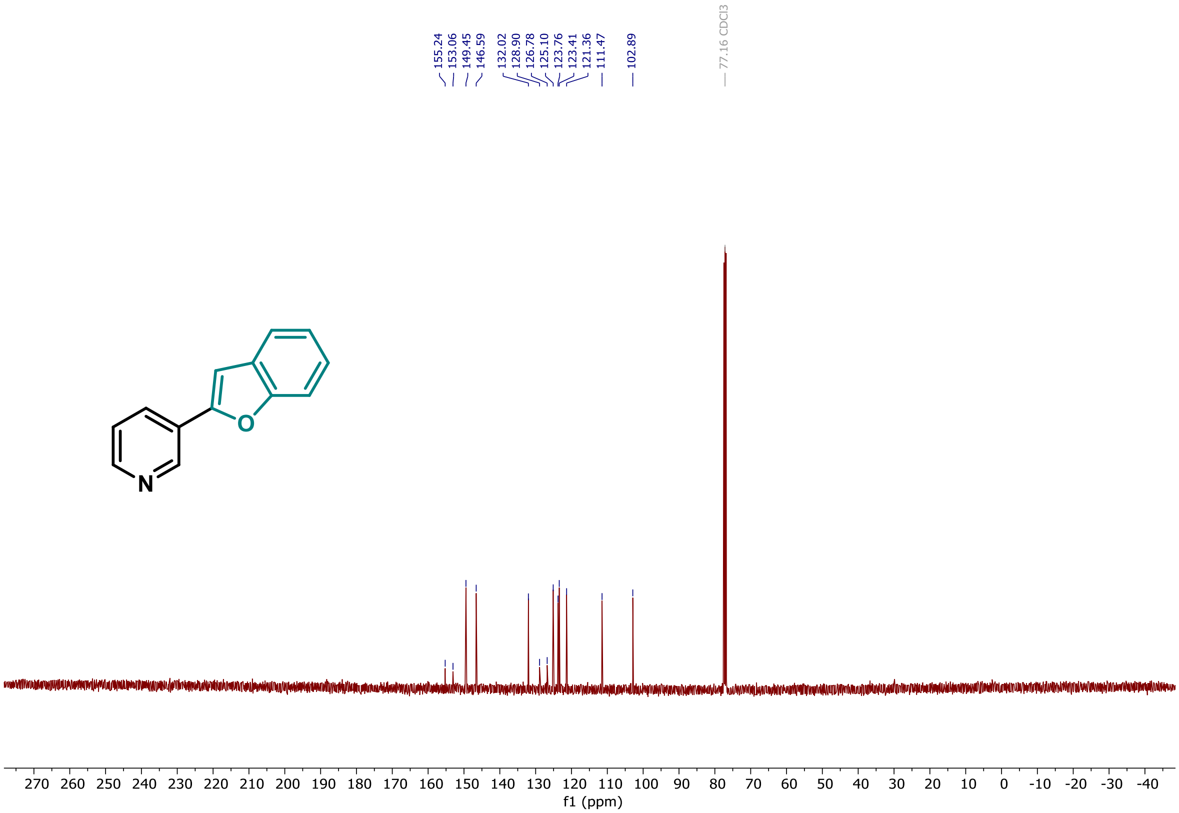

^{1}H NMR of 18 ($CDCl_3$, 400 MHz)

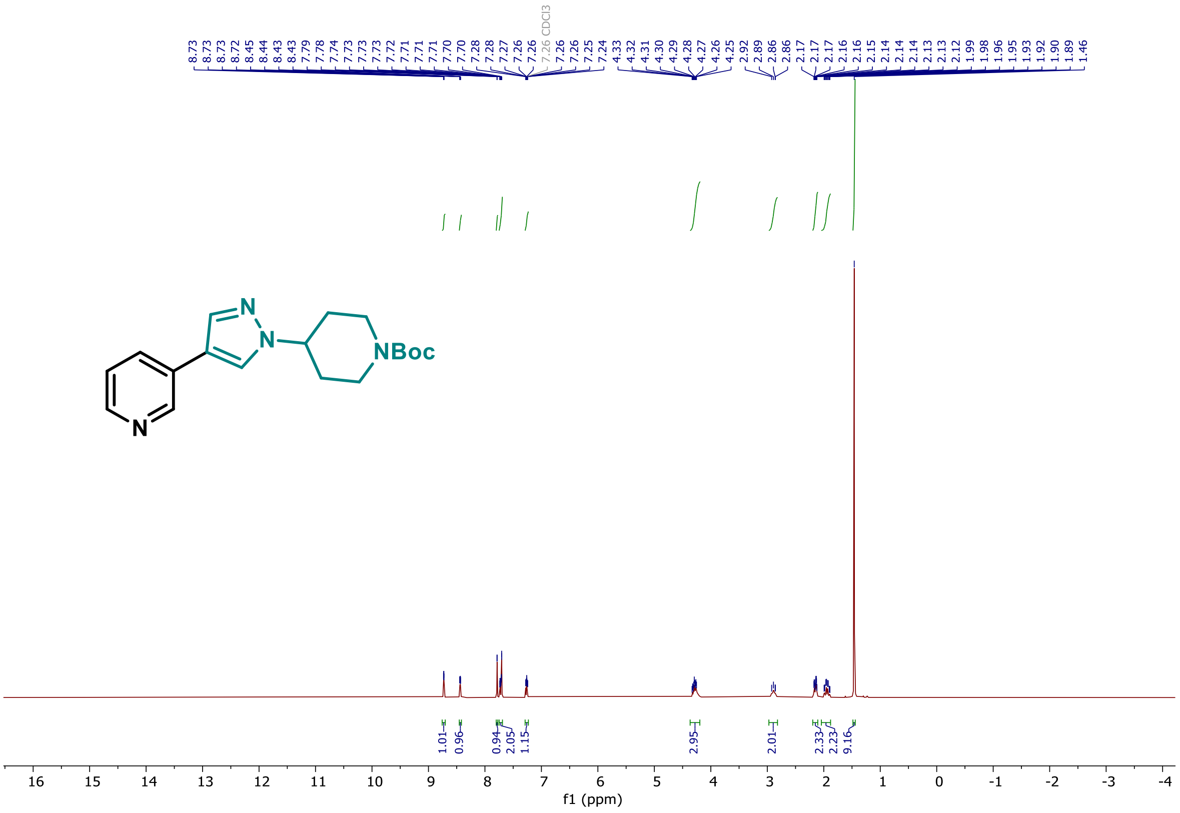

^{13}C NMR of 18 ($CDCl_3$, 101 MHz)

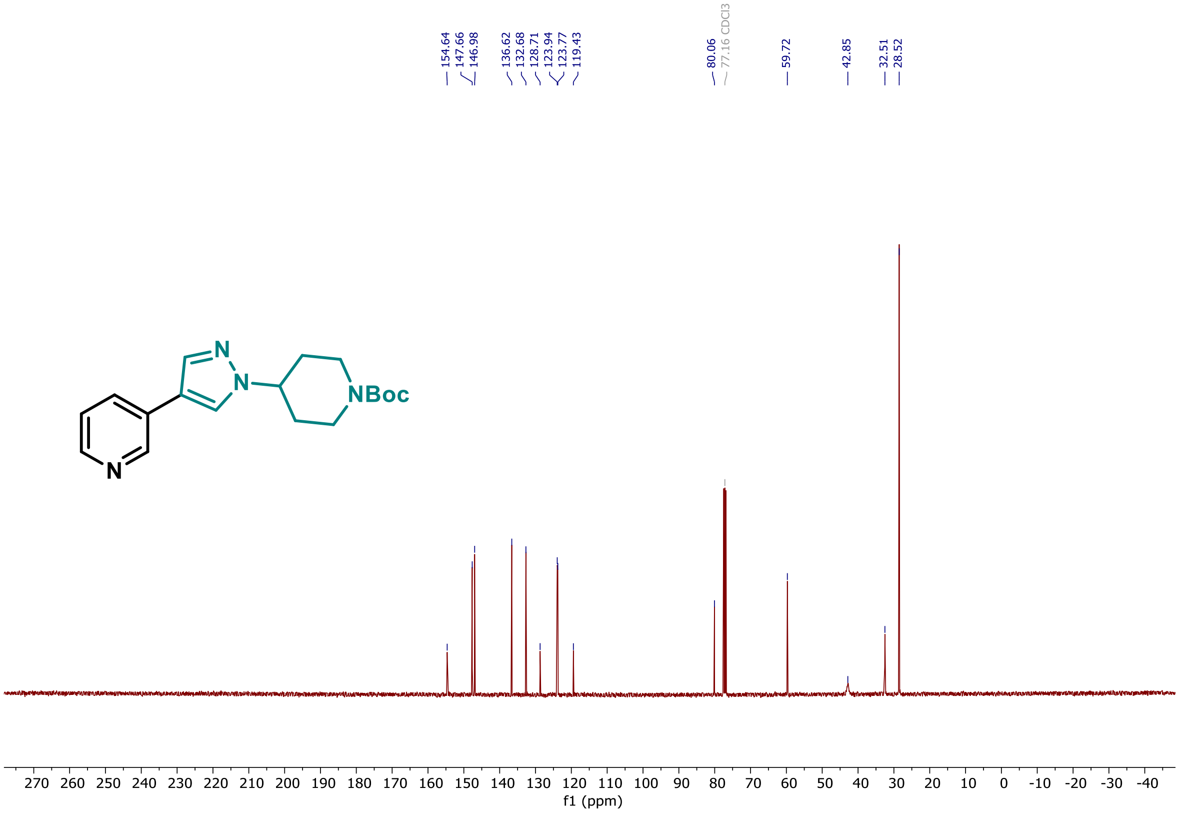

^{1}H NMR of 19 ($CDCl_3$, 400 MHz)

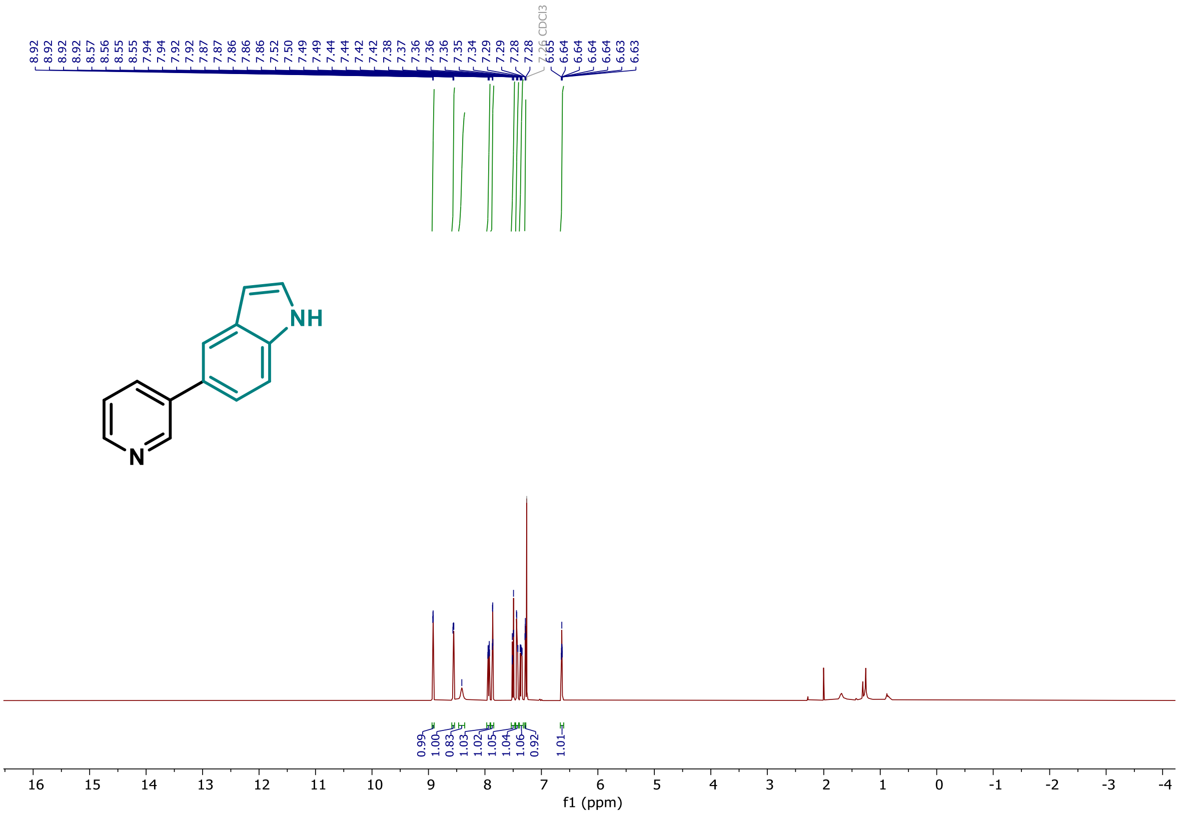

^{13}C NMR of 19 ($CDCl_3$, 101 MHz)

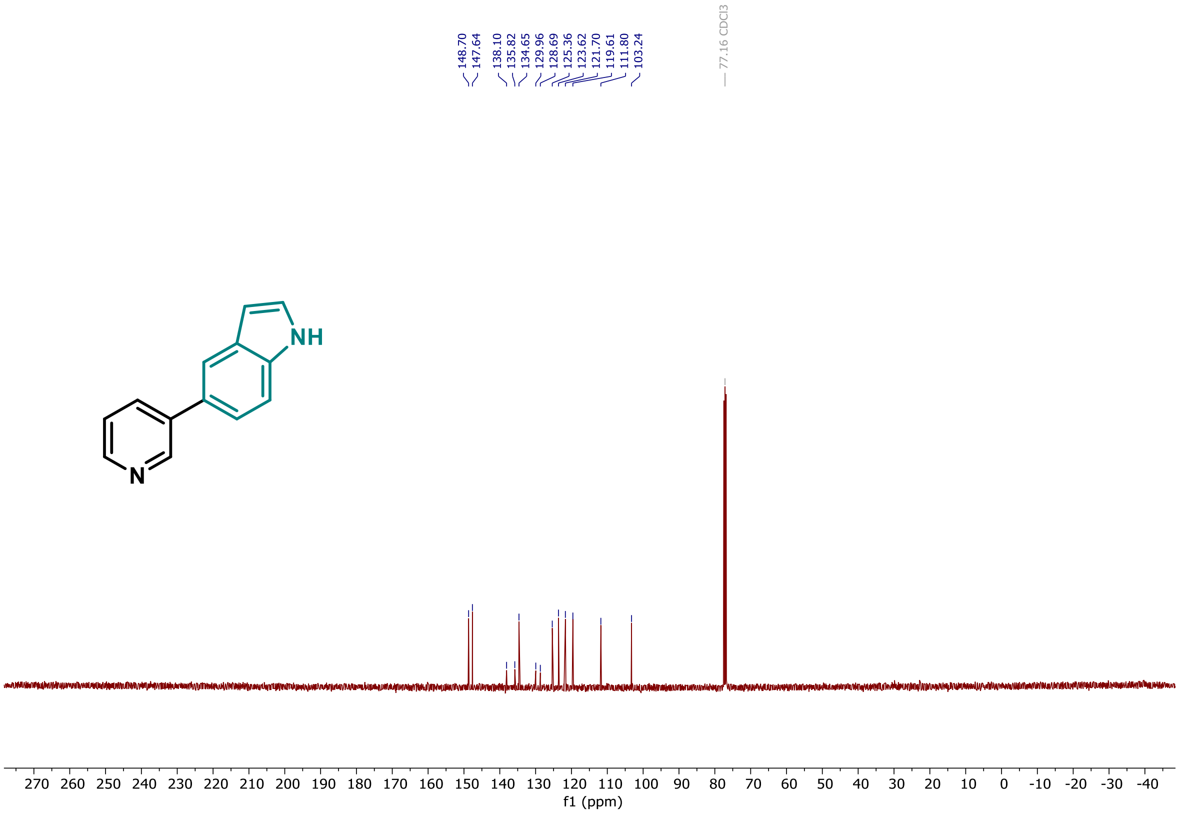

^{1}H NMR of 20 ($CDCl_3$, 400 MHz)

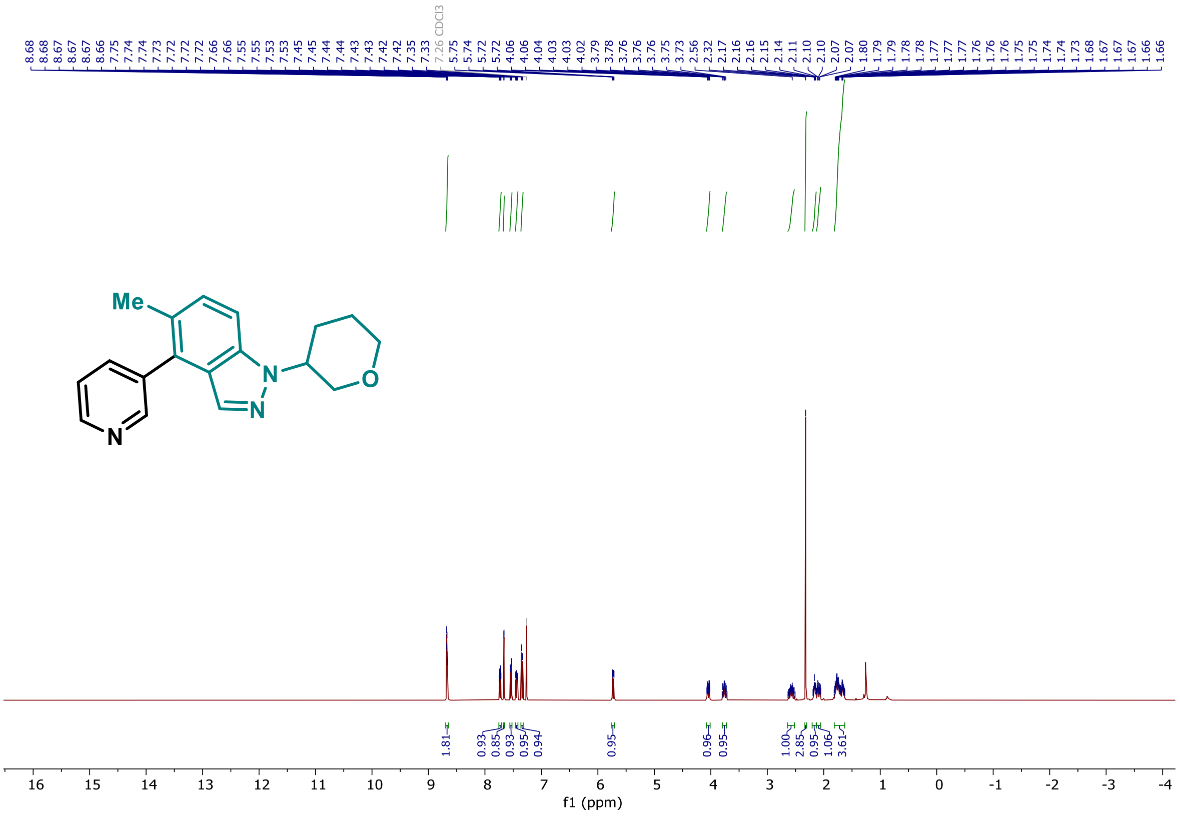

^{13}C NMR of 20 ($CDCl_3$, 101 MHz)

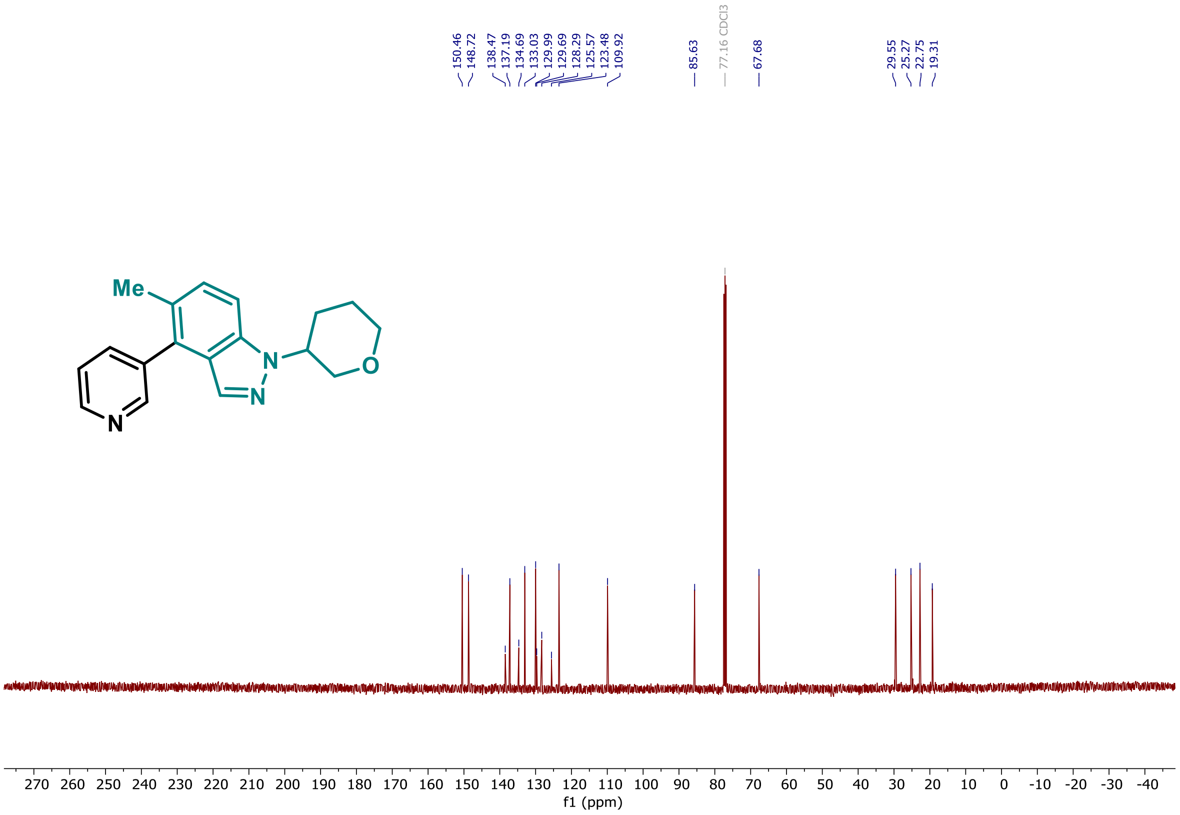

^{1}H NMR of 21 ($CDCl_3$, 300 MHz)

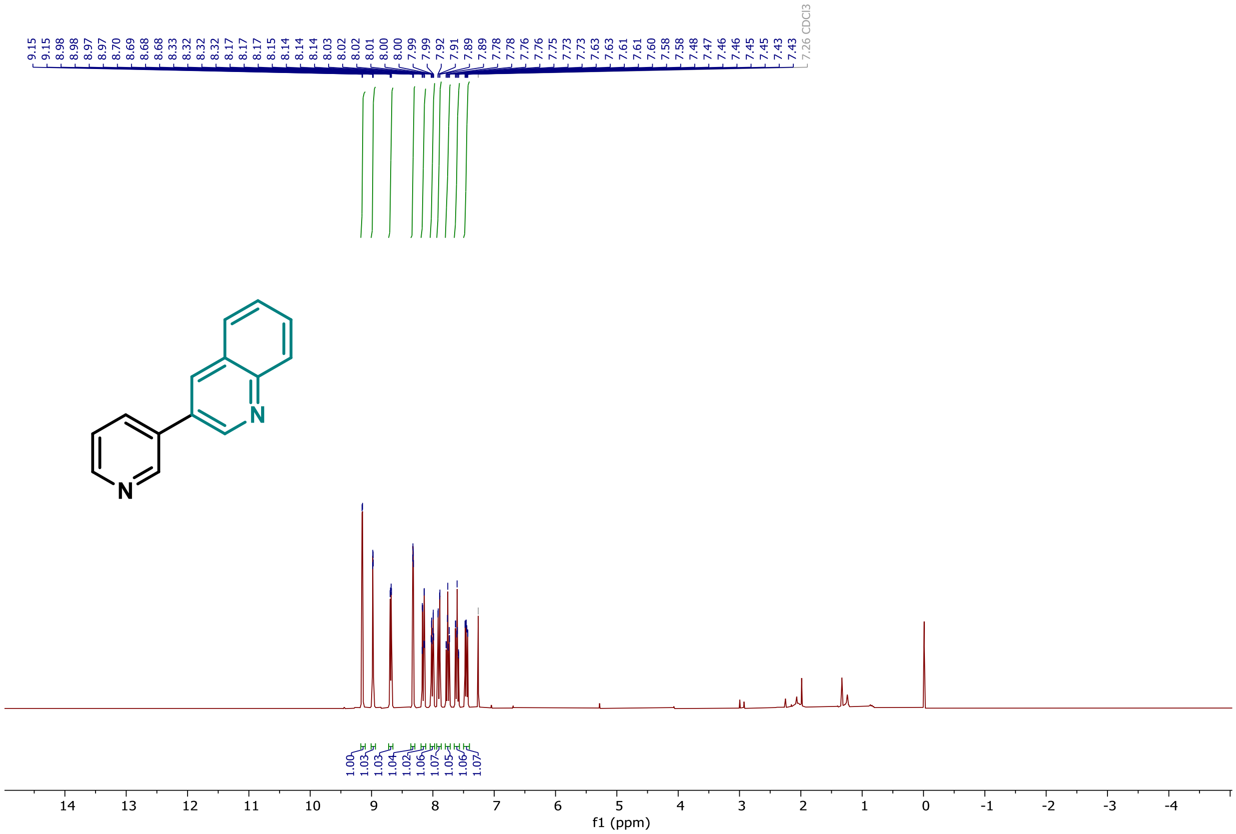

^{13}C NMR of 21 ($CDCl_3$, 75 MHz)

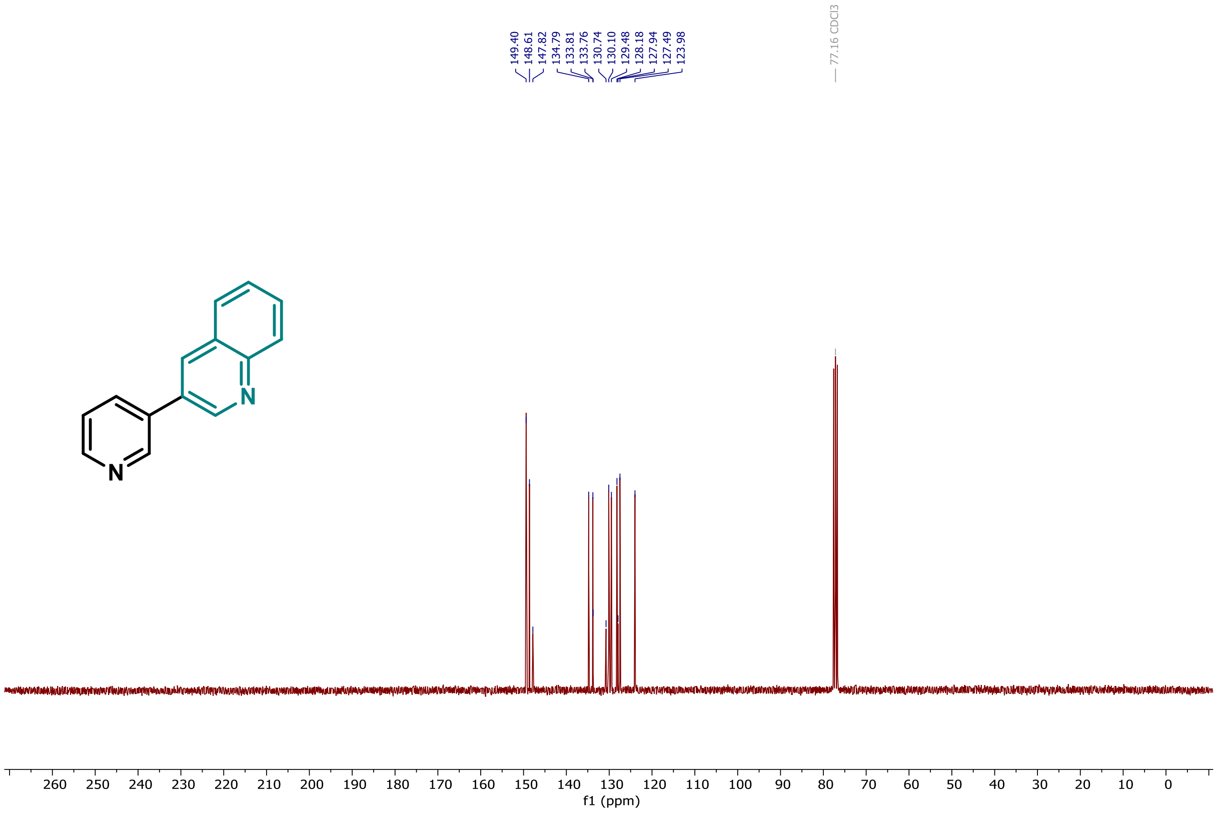

^{1}H NMR of 22 ($CDCl_3$, 400 MHz)

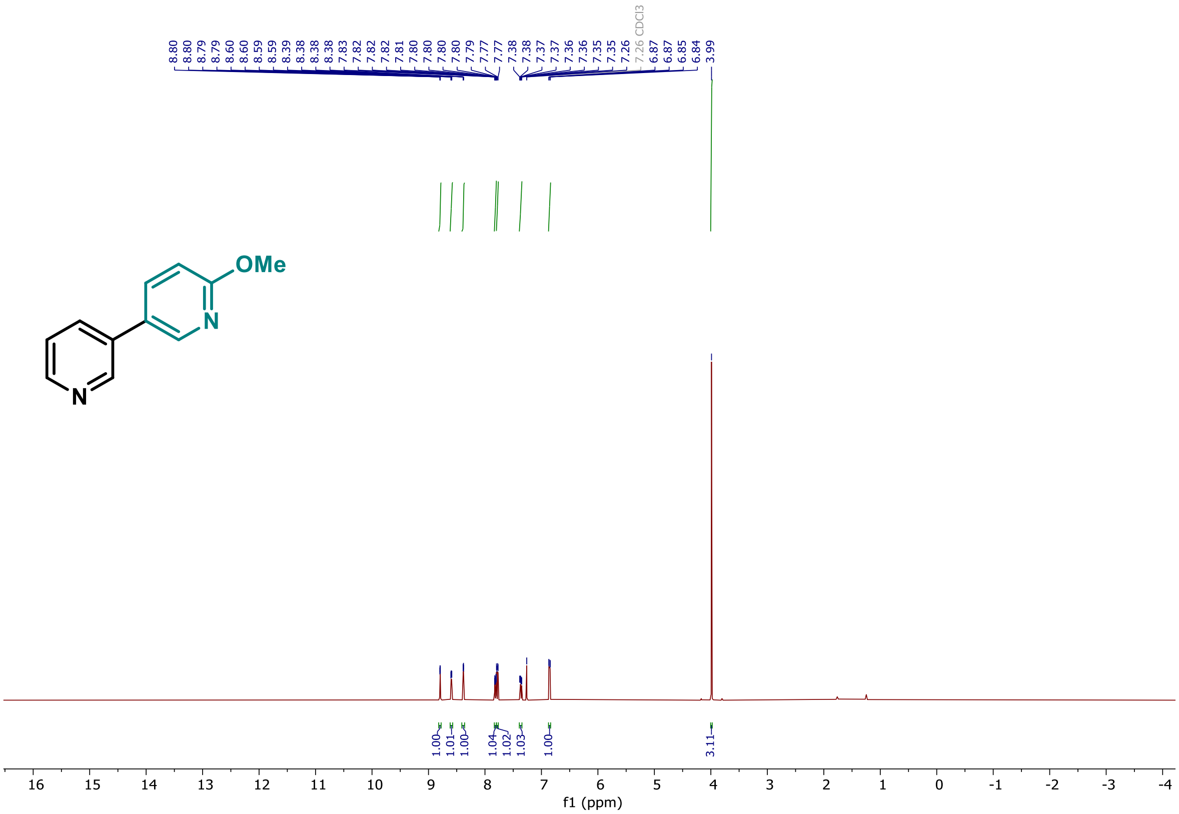

^{13}C NMR of 22 ($CDCl_3$, 101 MHz)

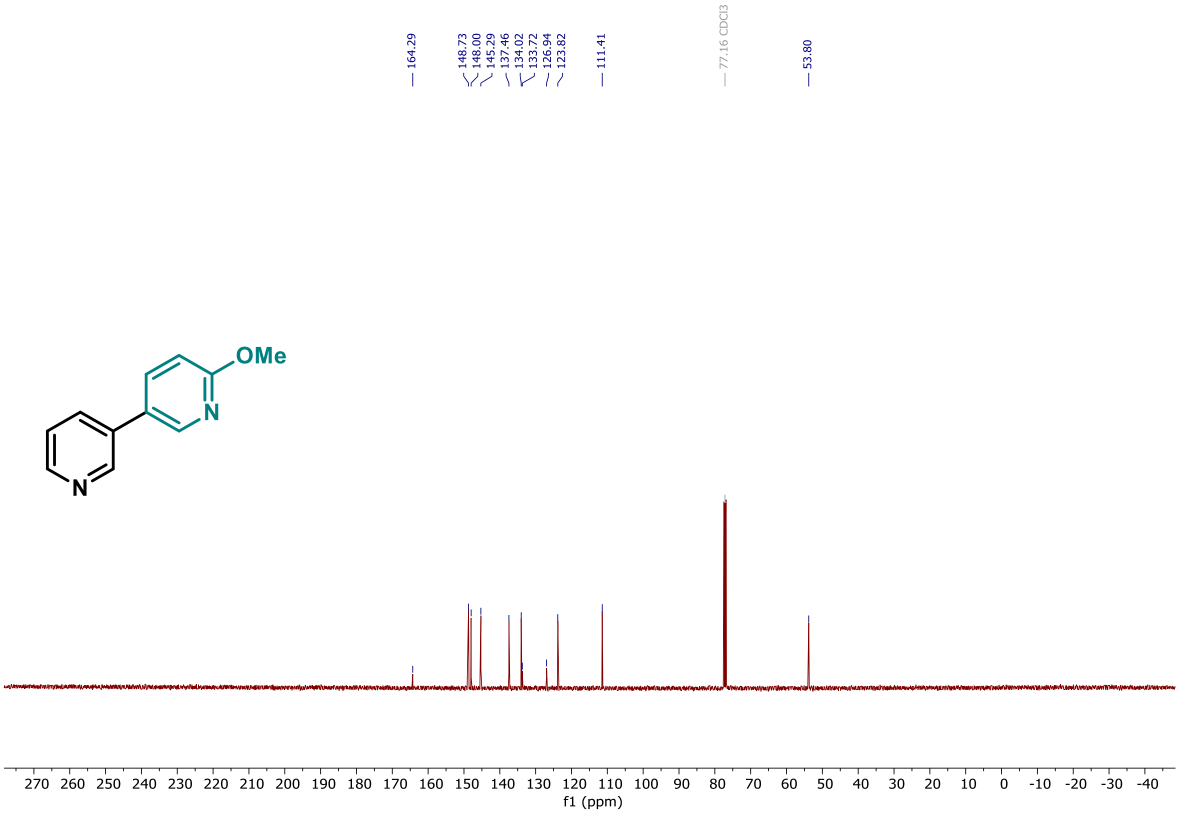

^{1}H NMR of 23 ($CDCl_3$, 400 MHz)

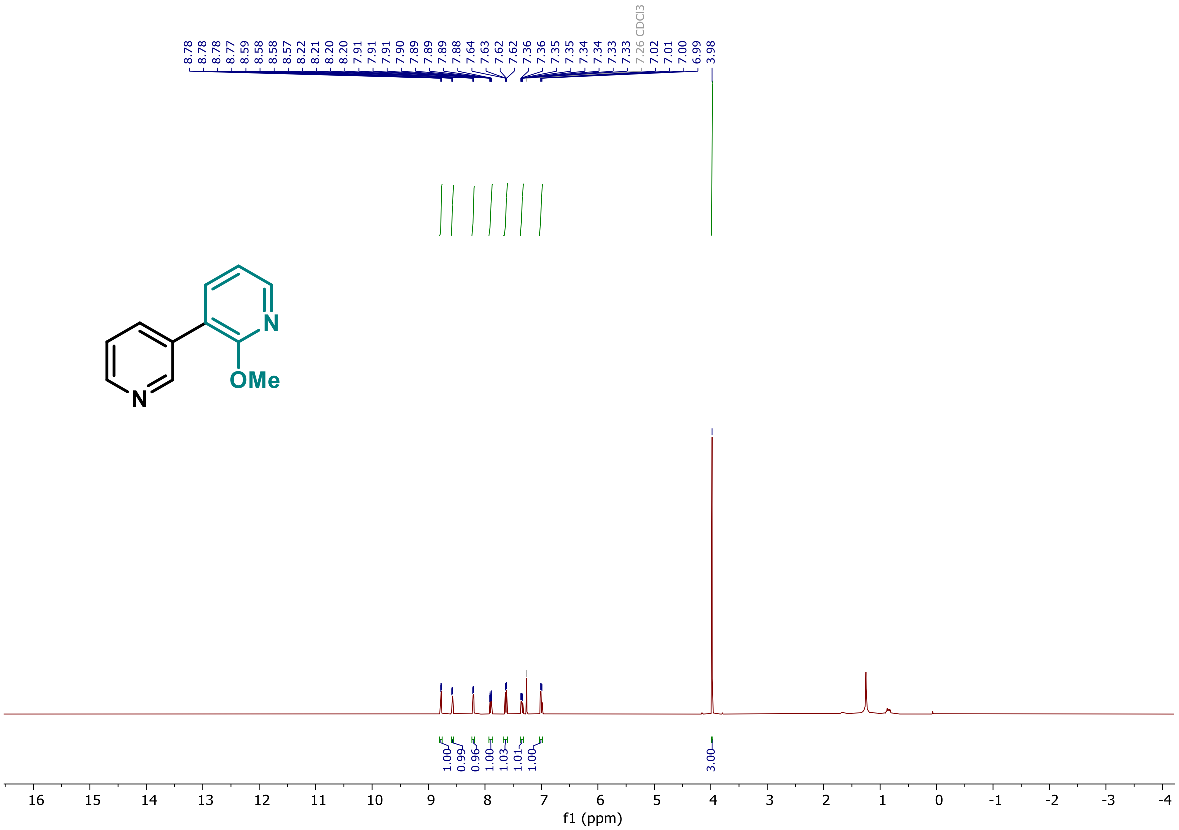

^{13}C NMR of 23 ($CDCl_3$, 101 MHz)

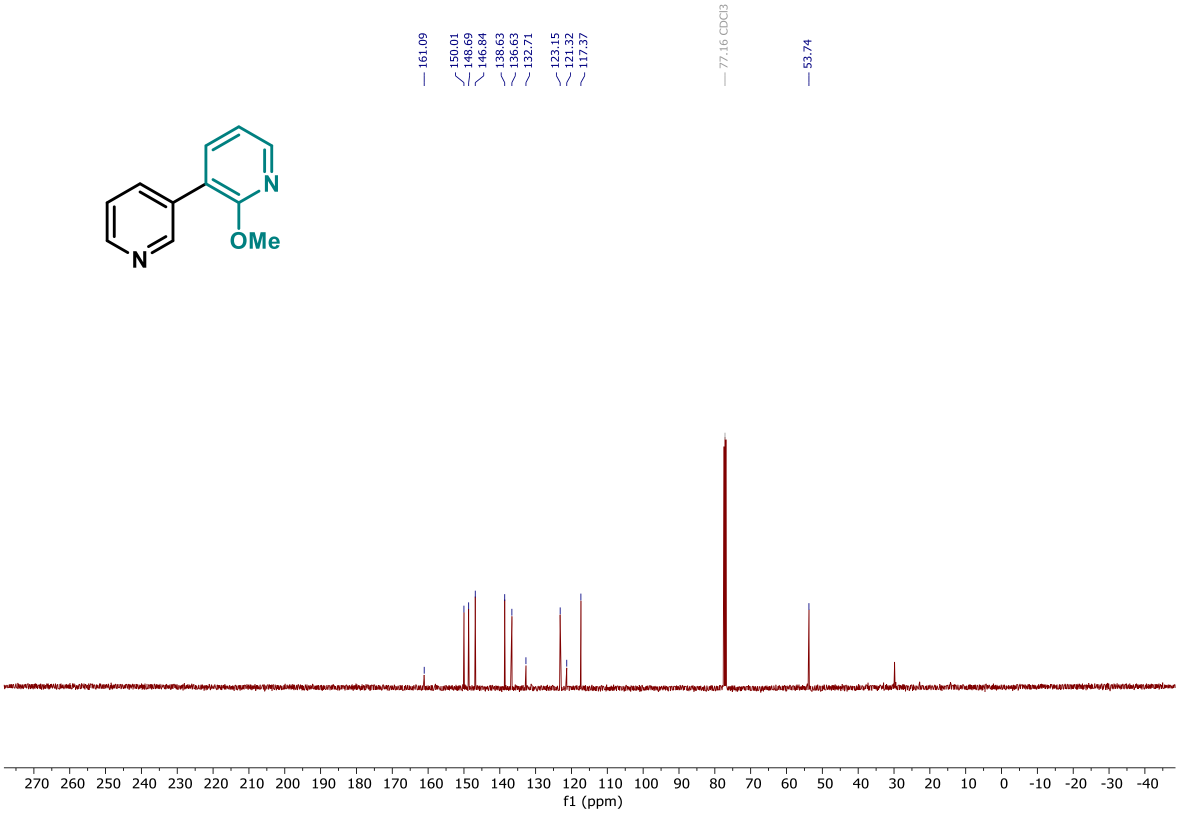

^{1}H NMR of 24 ($CDCl_3$, 300 MHz)

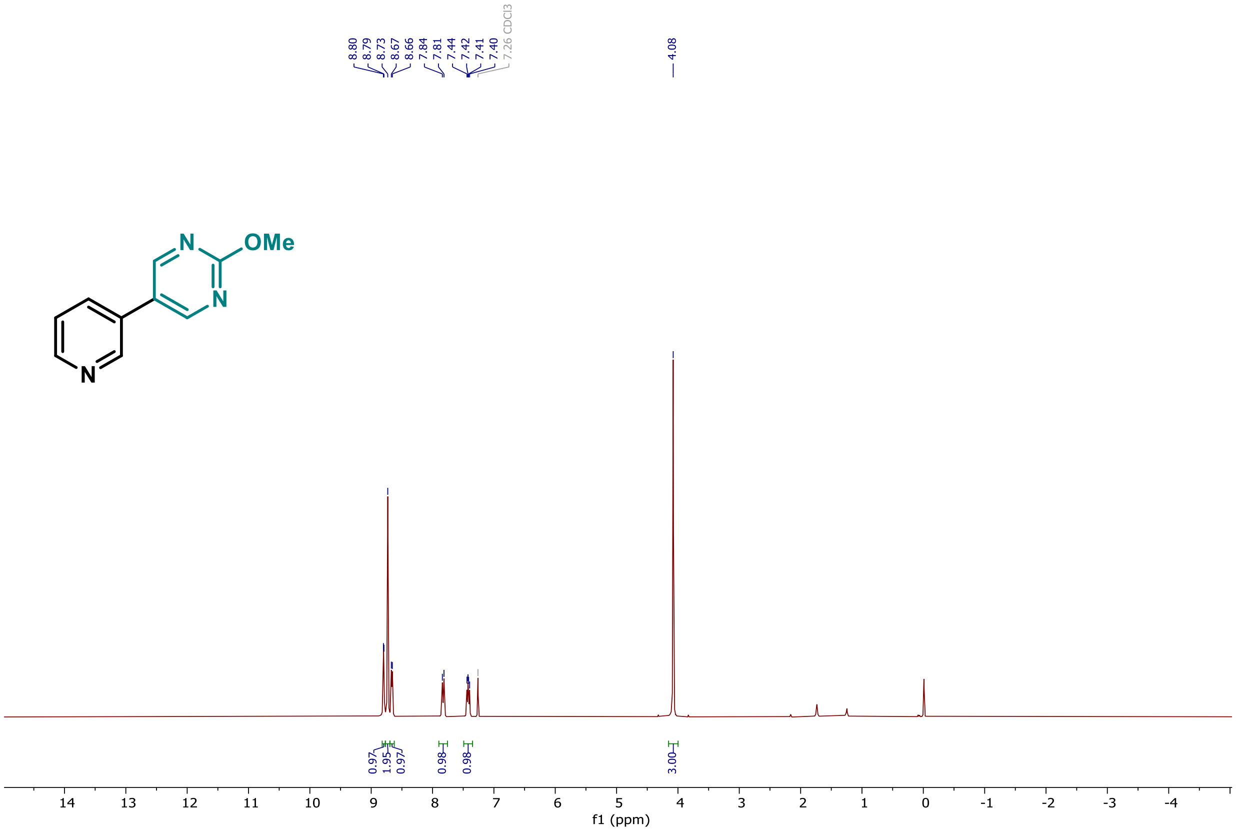

^{13}C NMR of 24 ($CDCl_3$, 75 MHz)

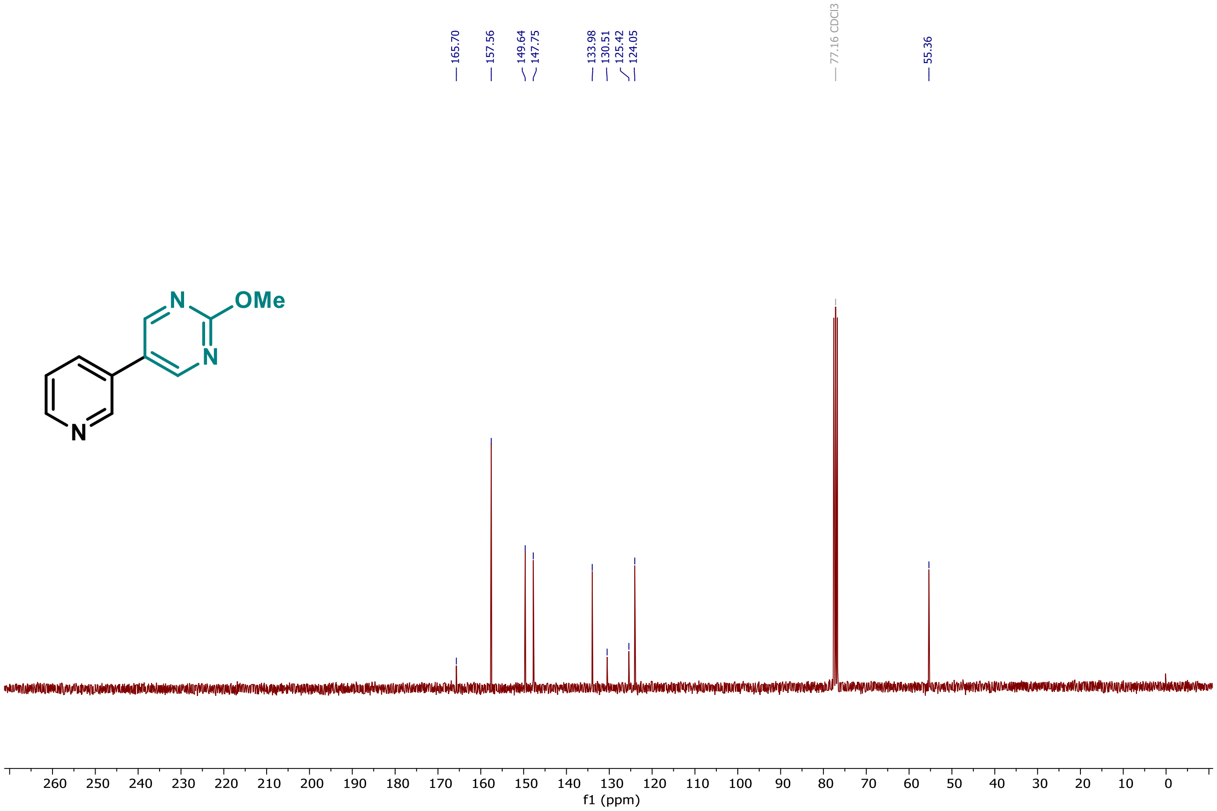

^{1}H NMR of 25 ($CDCl_3$, 400 MHz)

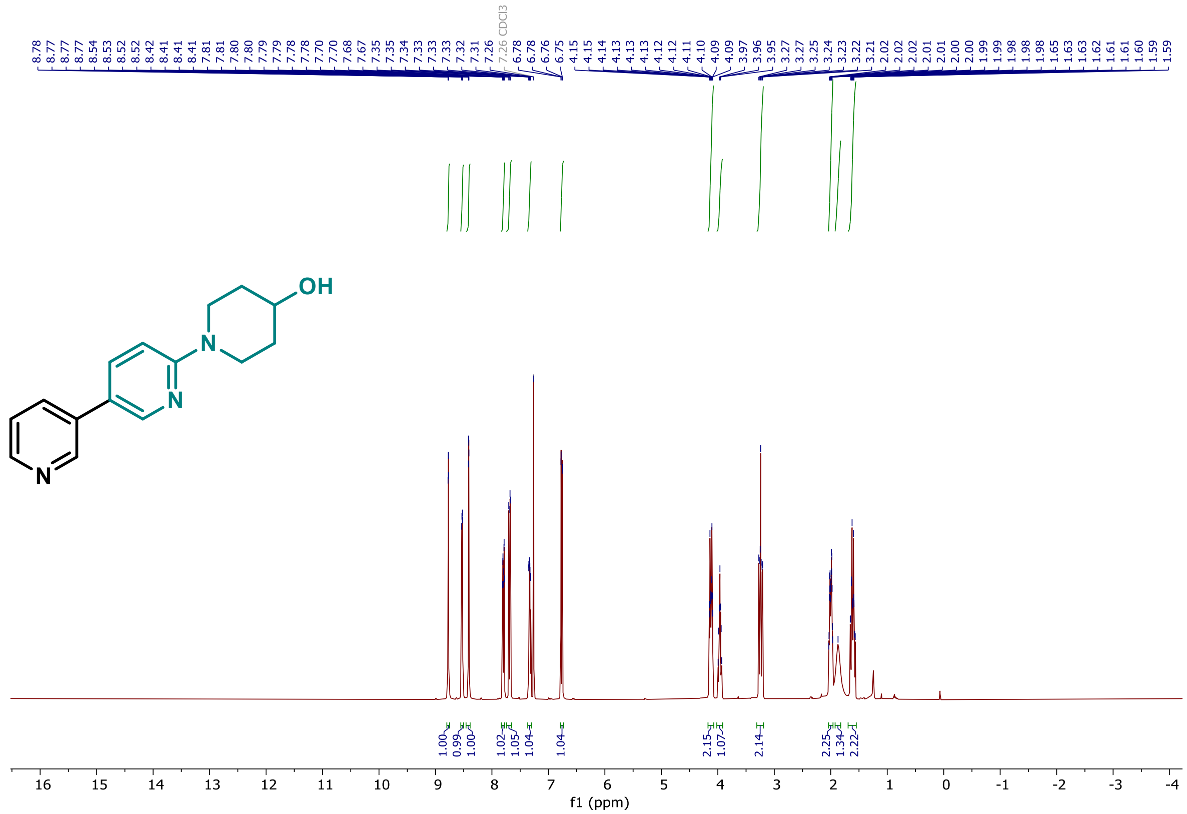

^{13}C NMR of 25 ($CDCl_3$, 101 MHz)

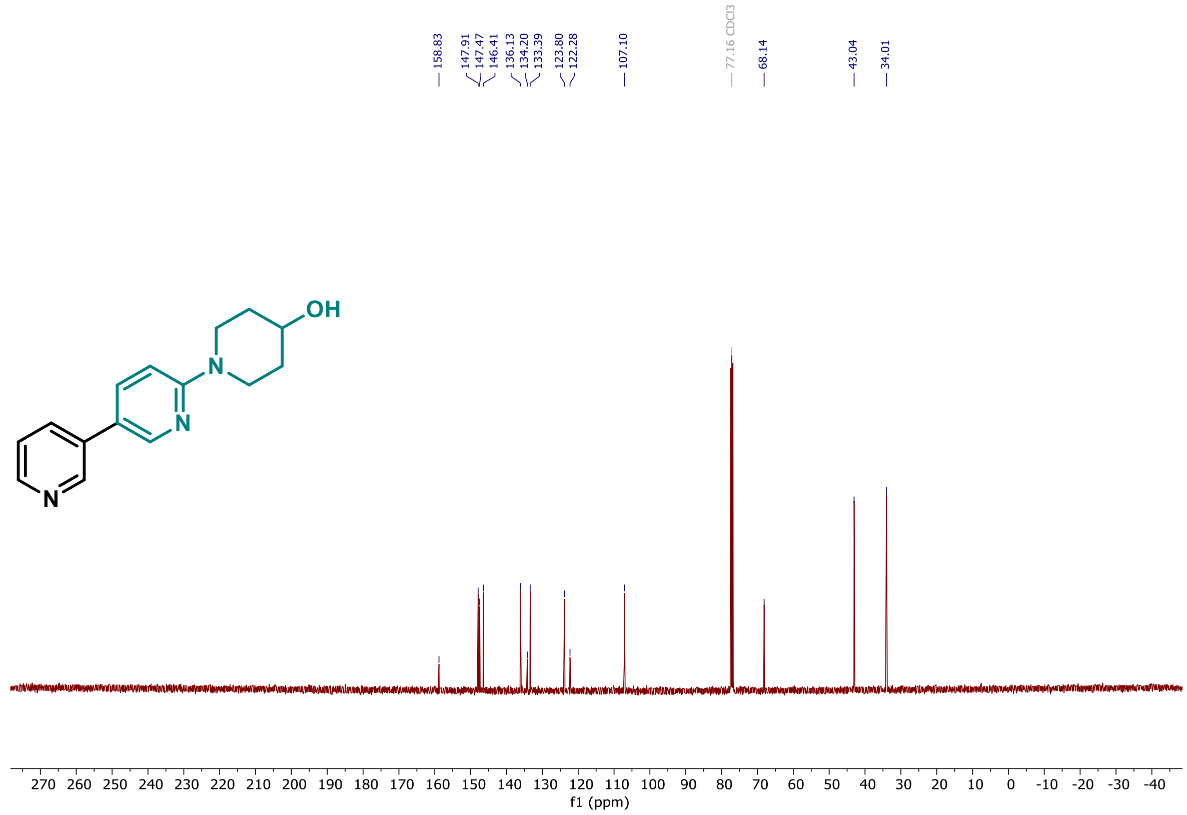

^{1}H NMR of 26 ($CDCl_3$, 400 MHz)

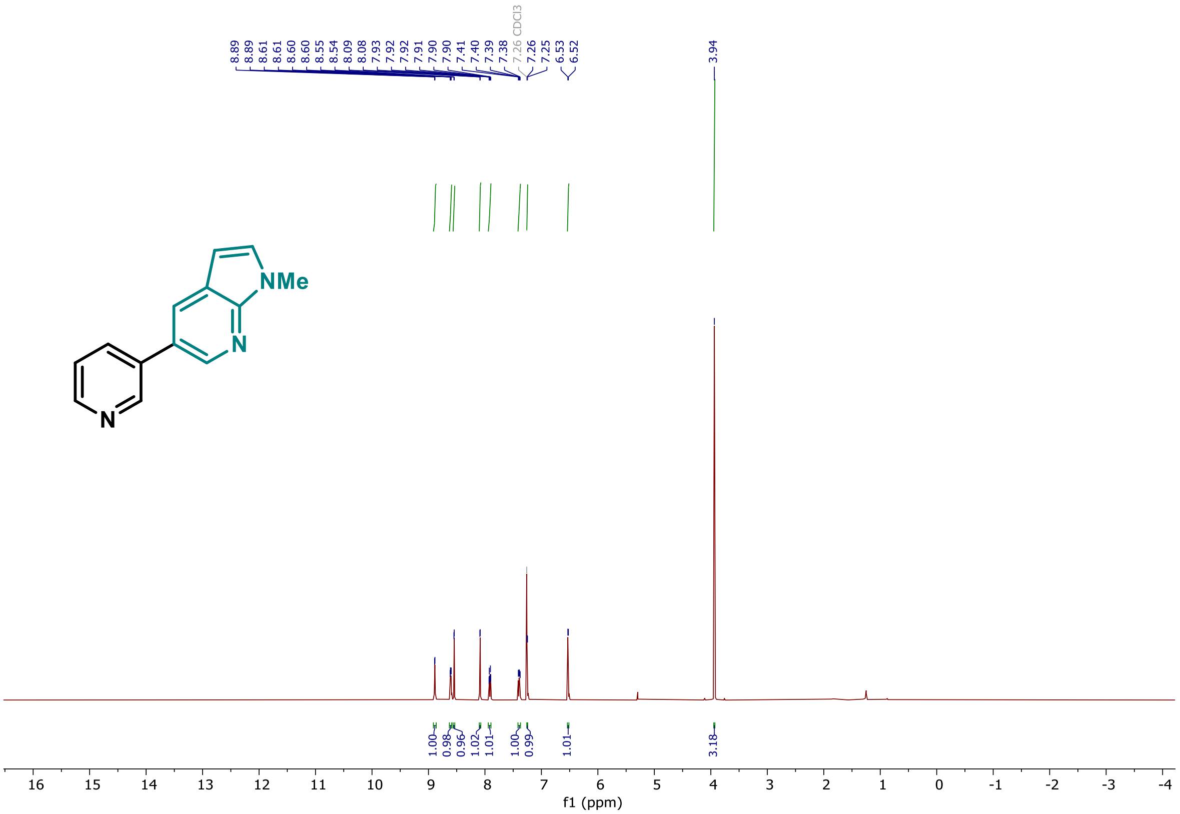

^{13}C NMR of 26 ($CDCl_3$, 101 MHz)

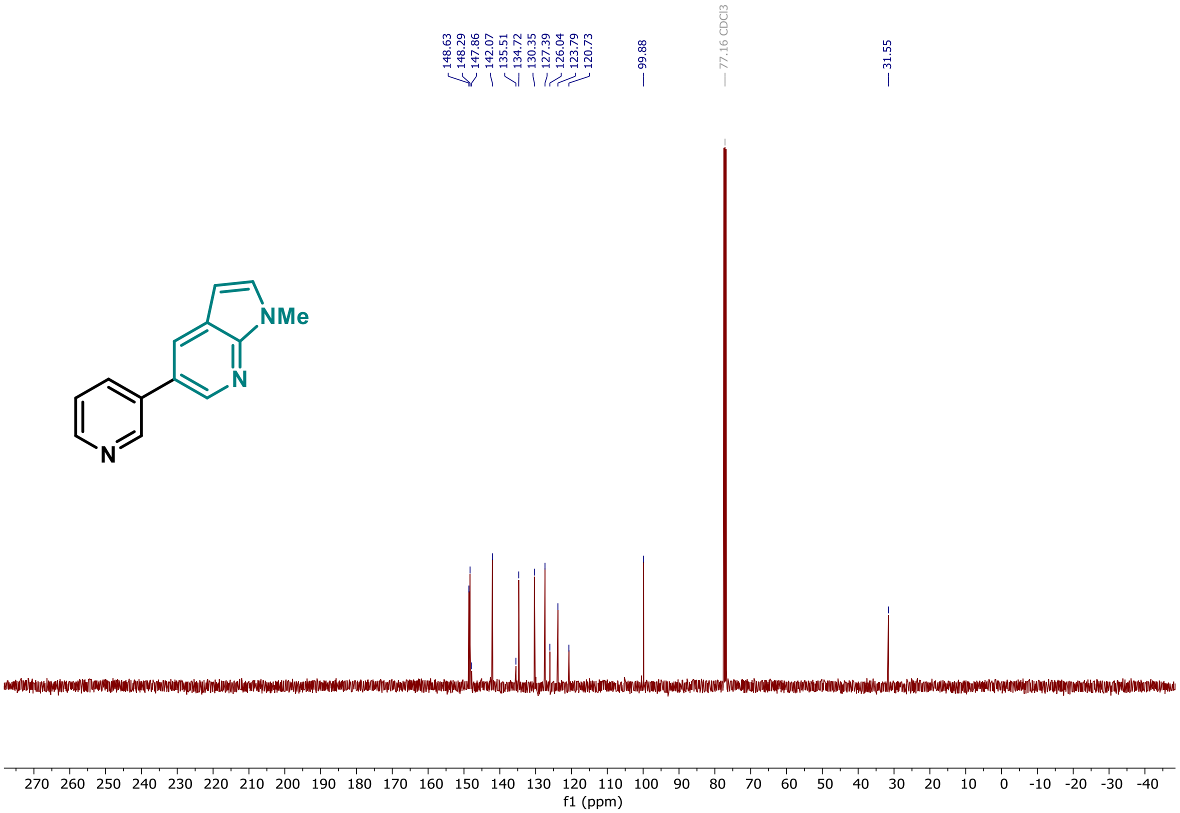

^{1}H NMR of 27 ($CDCl_3$, 400 MHz)

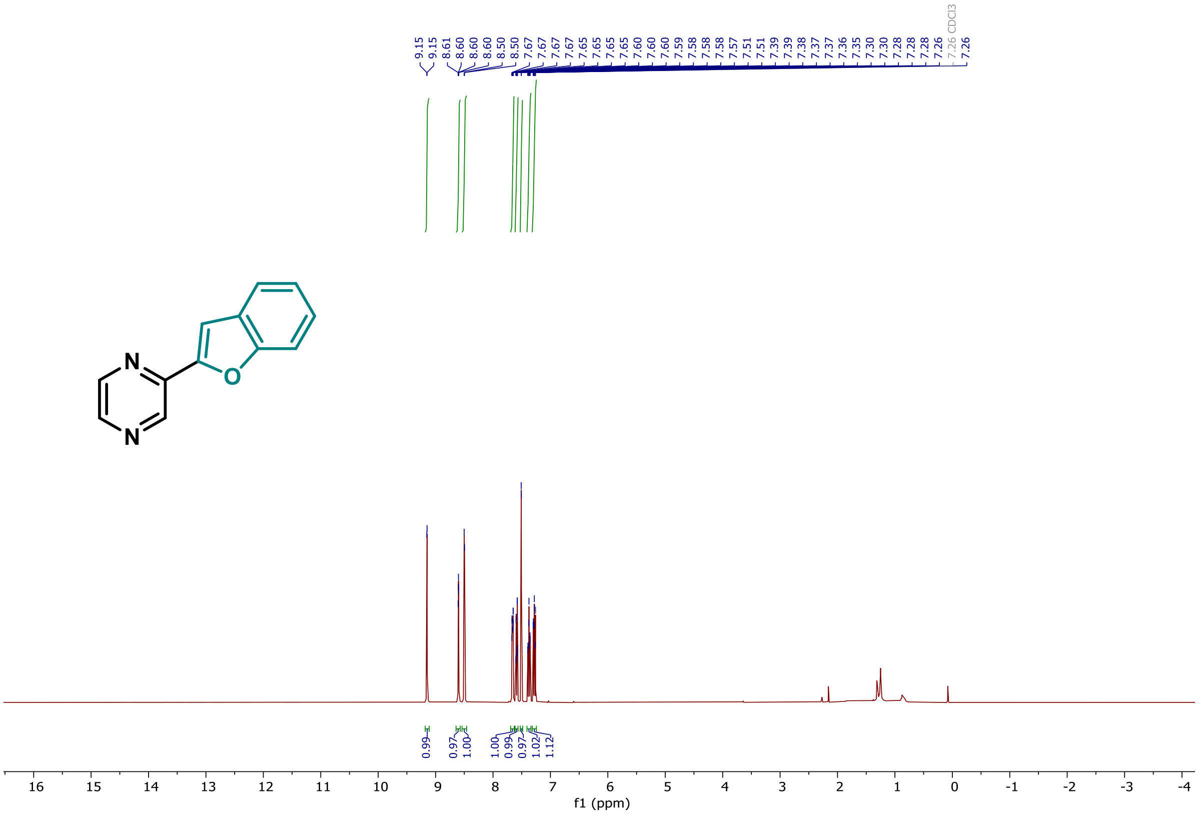

^{13}C NMR of 27 ($CDCl_3$, 101 MHz)

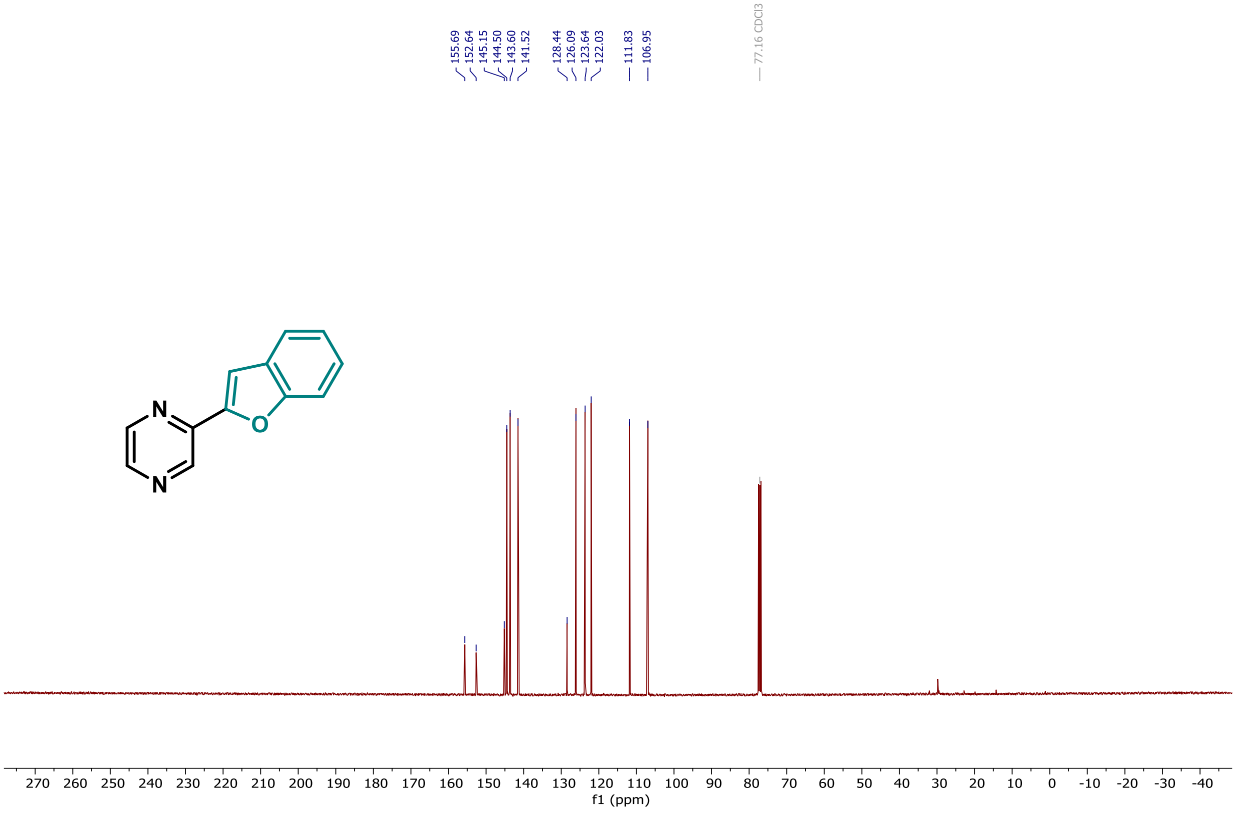

^{1}H NMR of 28 ($CDCl_3$, 300 MHz)

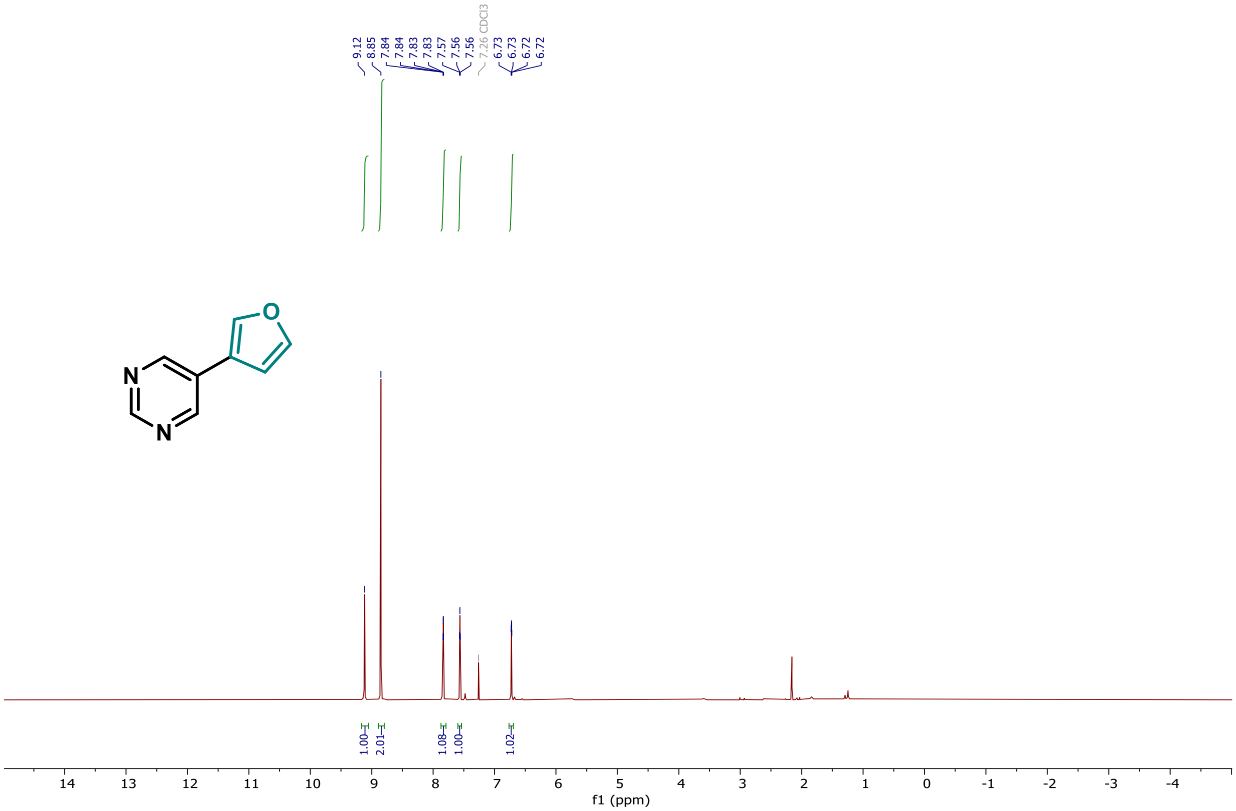

^{13}C NMR of 28 ($CDCl_3$, 75 MHz)

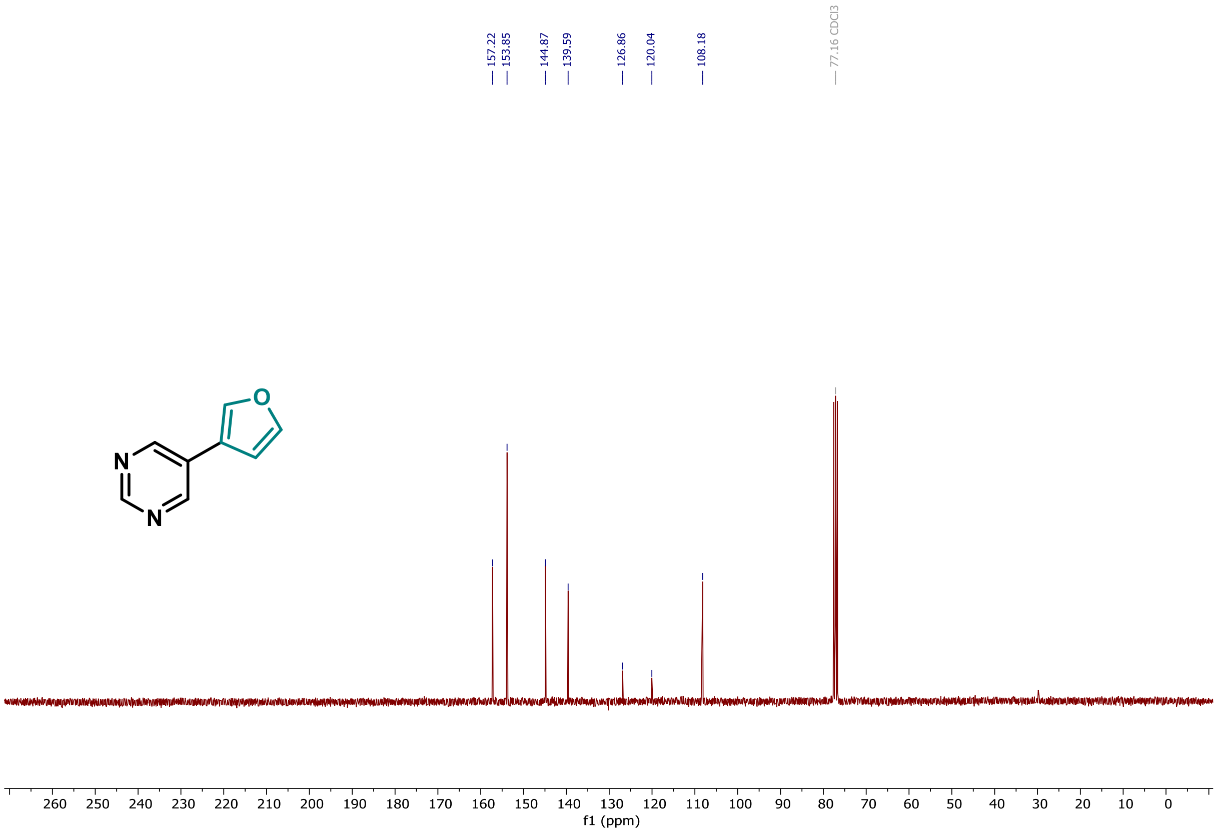

^{1}H NMR of 29 ($CDCl_3$, 300 MHz)

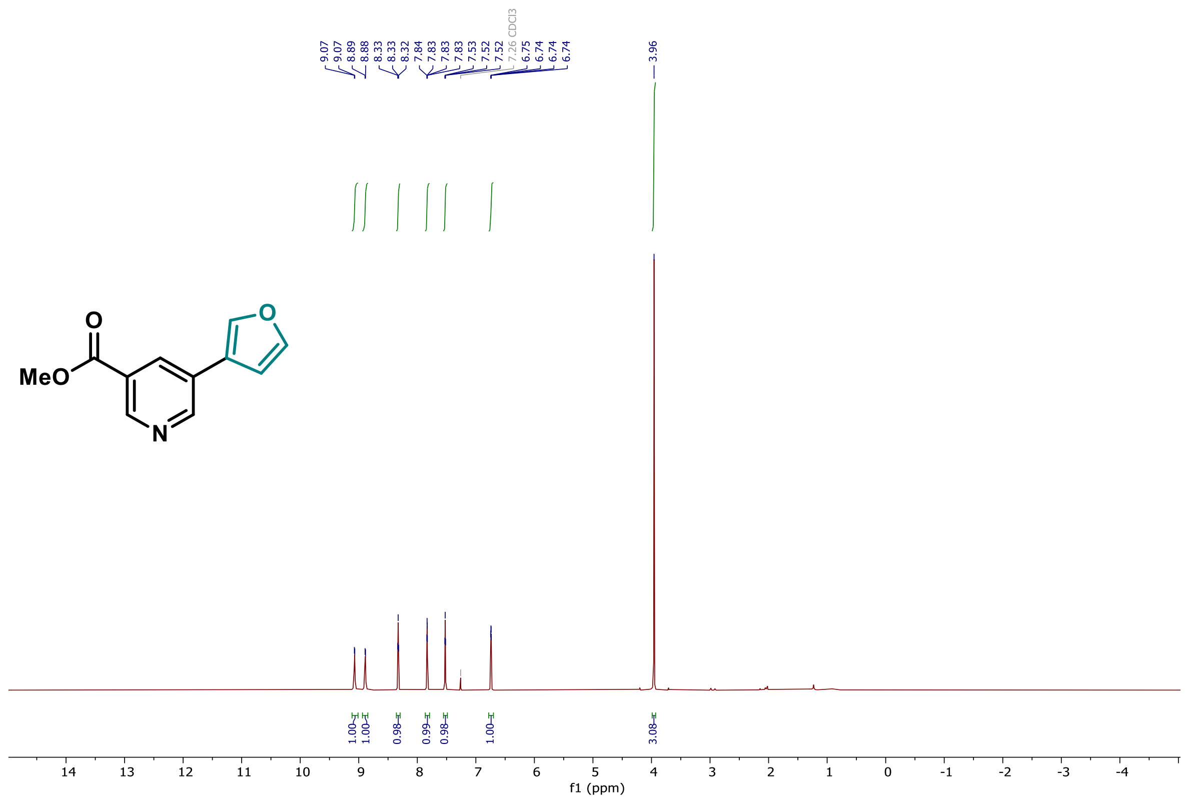

^{13}C NMR of 29 ($CDCl_3$, 101 MHz)

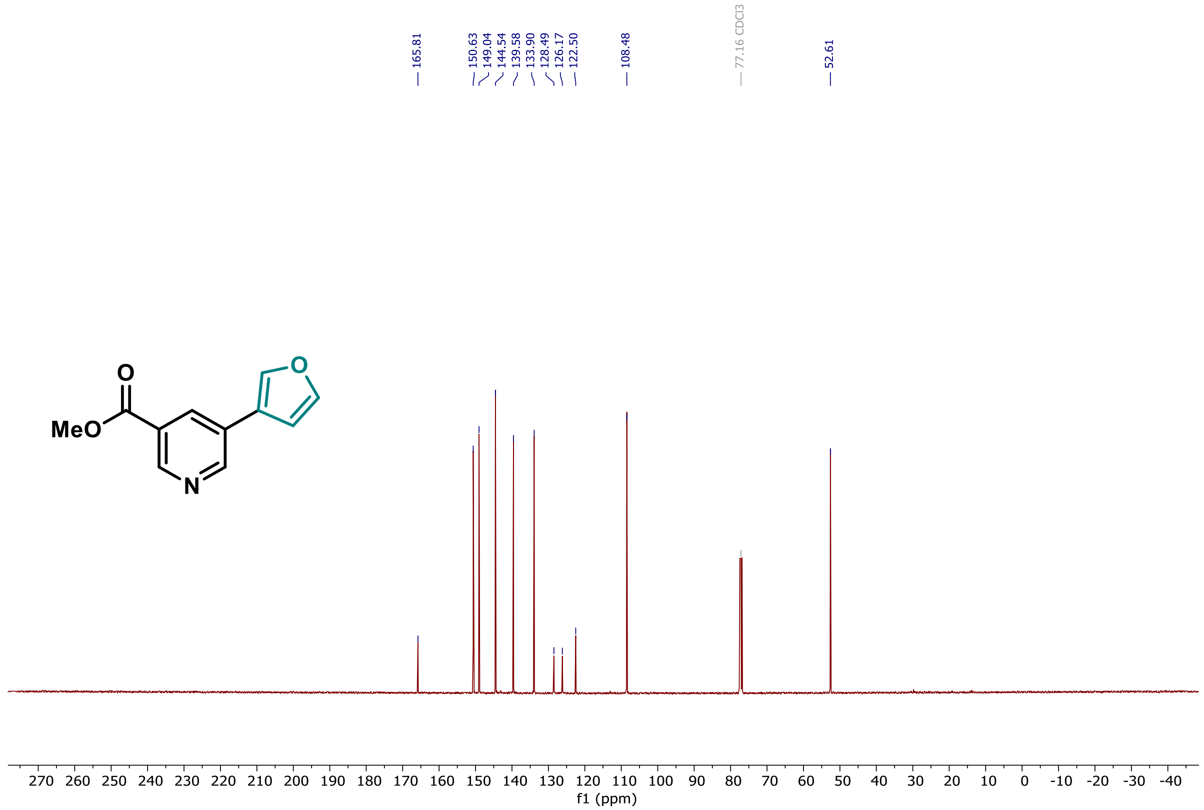

^{1}H NMR of 30 ($CDCl_3$, 300 MHz)

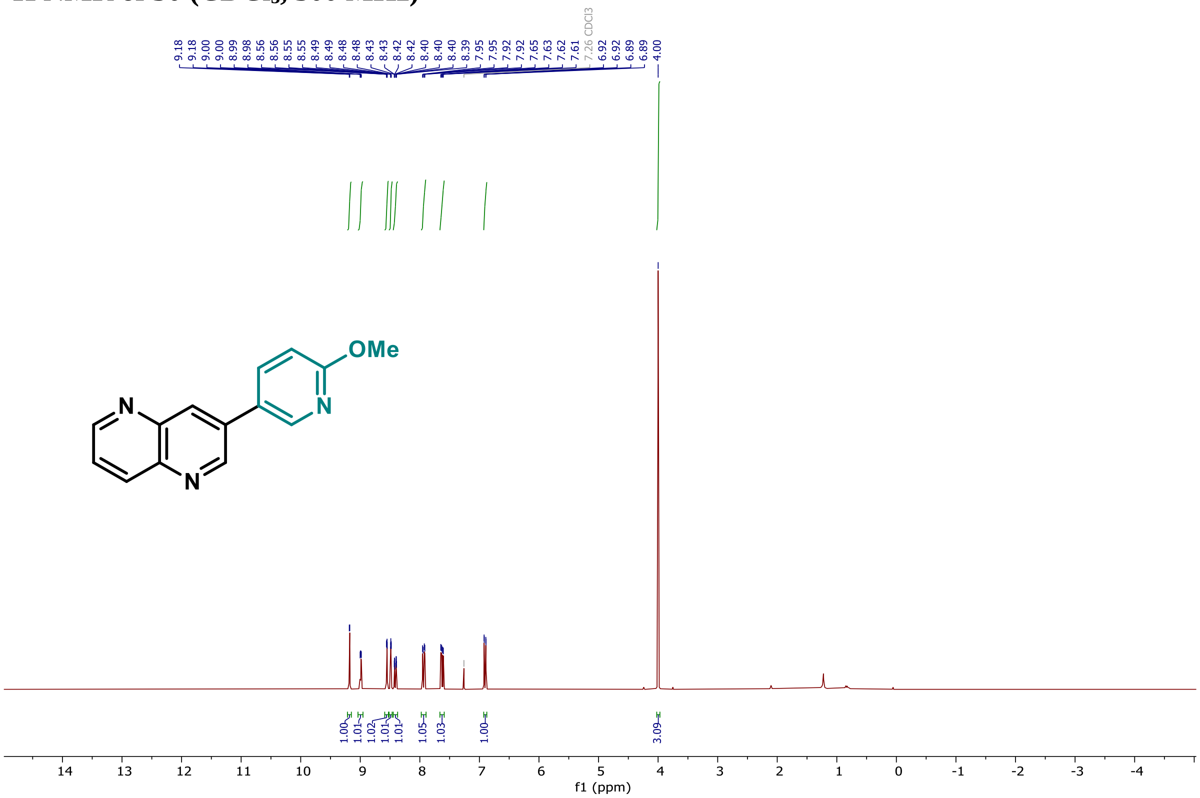

^{13}C NMR of 30 ($CDCl_3$, 101 MHz)

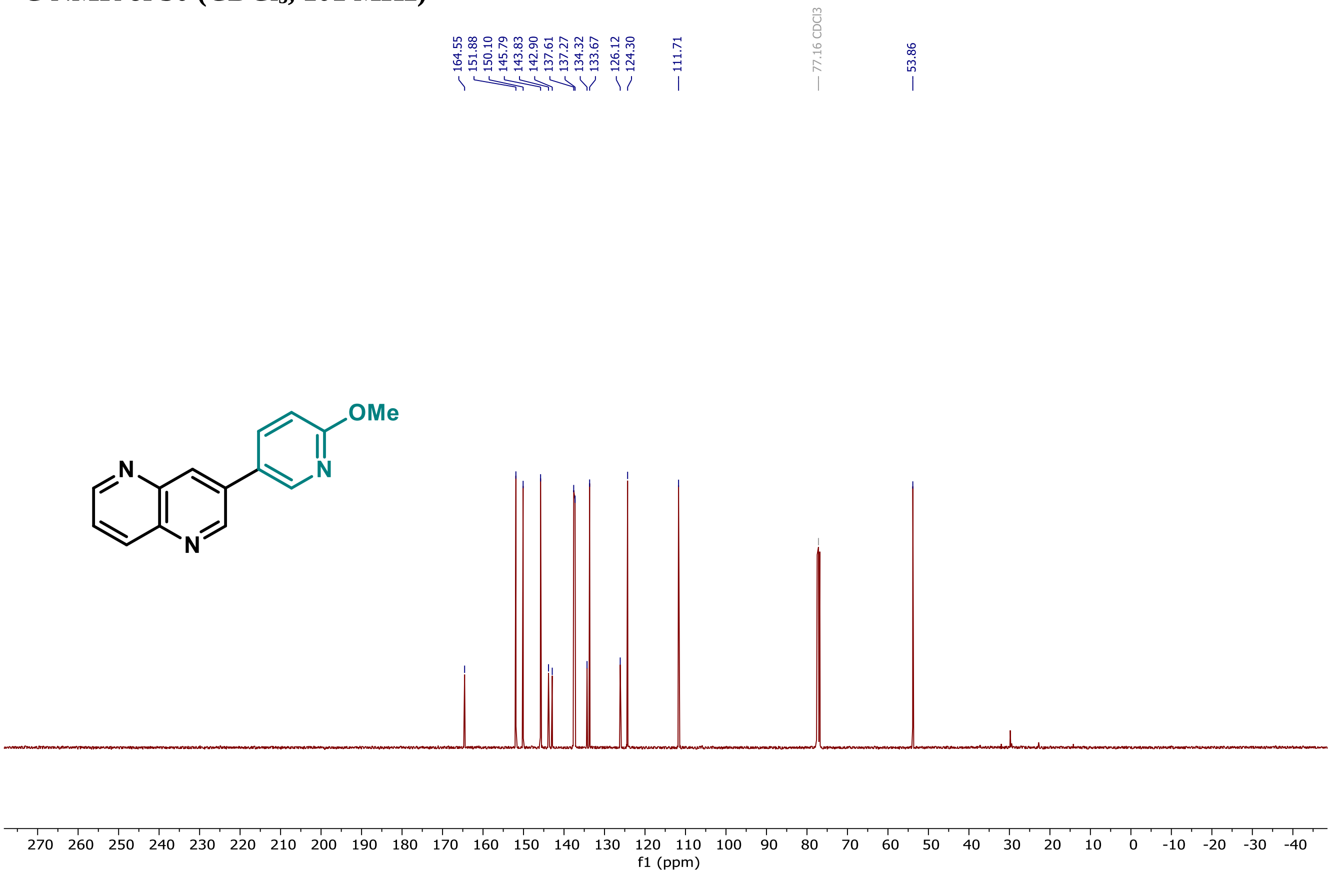

^{1}H NMR of 31 ($CDCl_3$, 400 MHz)

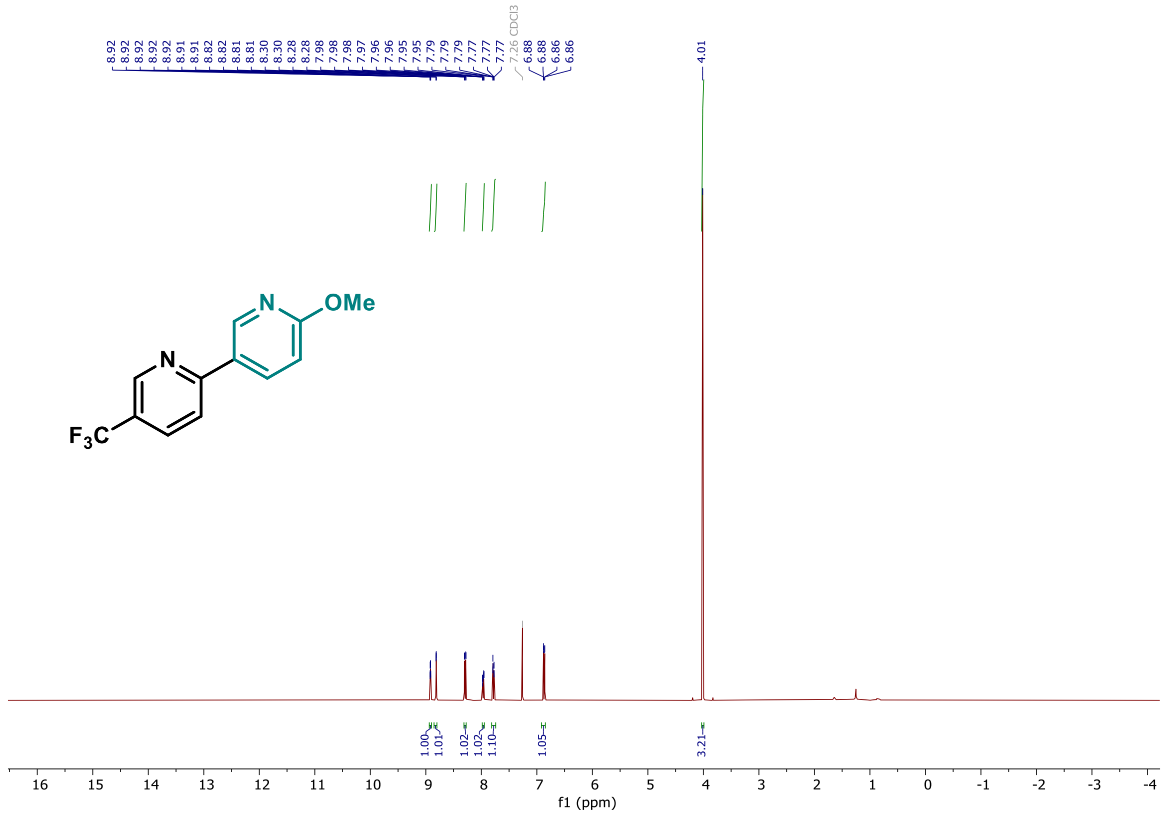

^{13}C NMR of 31 ($CDCl_3$, 101 MHz)

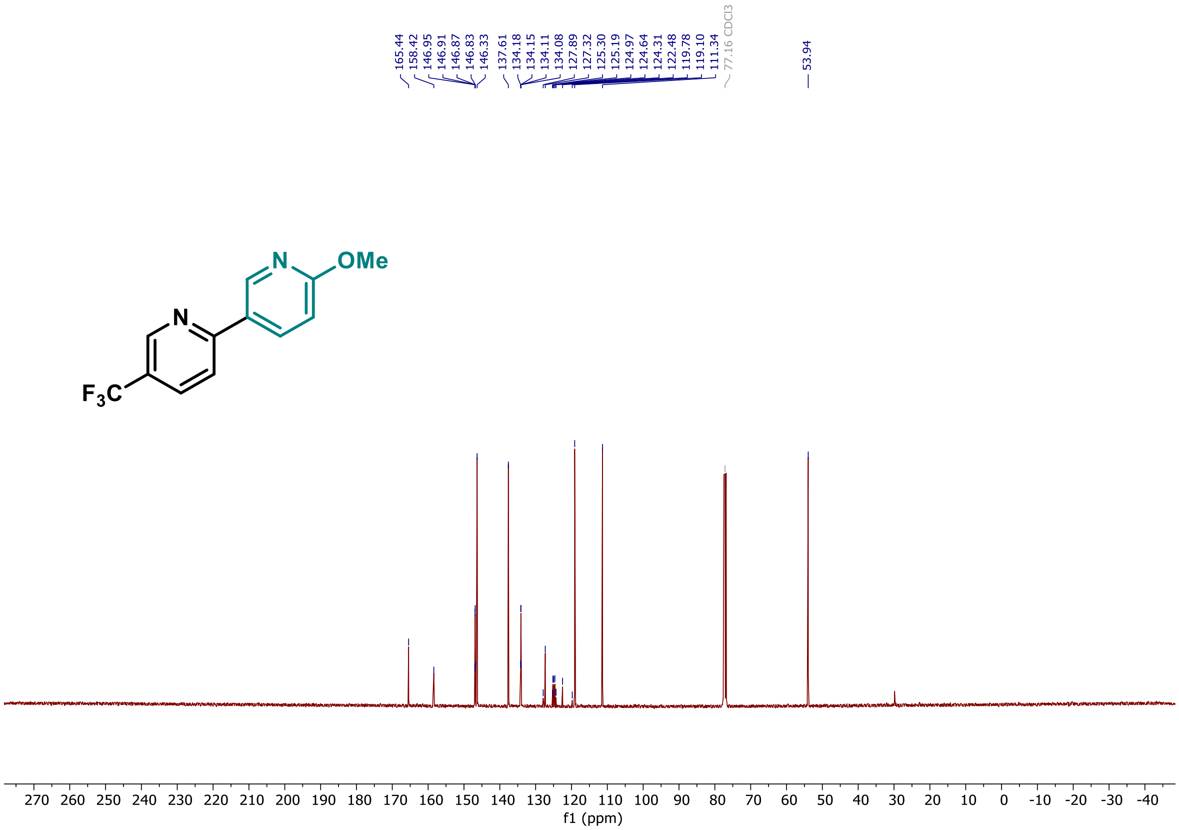

^{19}F NMR of 31 ($CDCl_3$, 282 MHz)

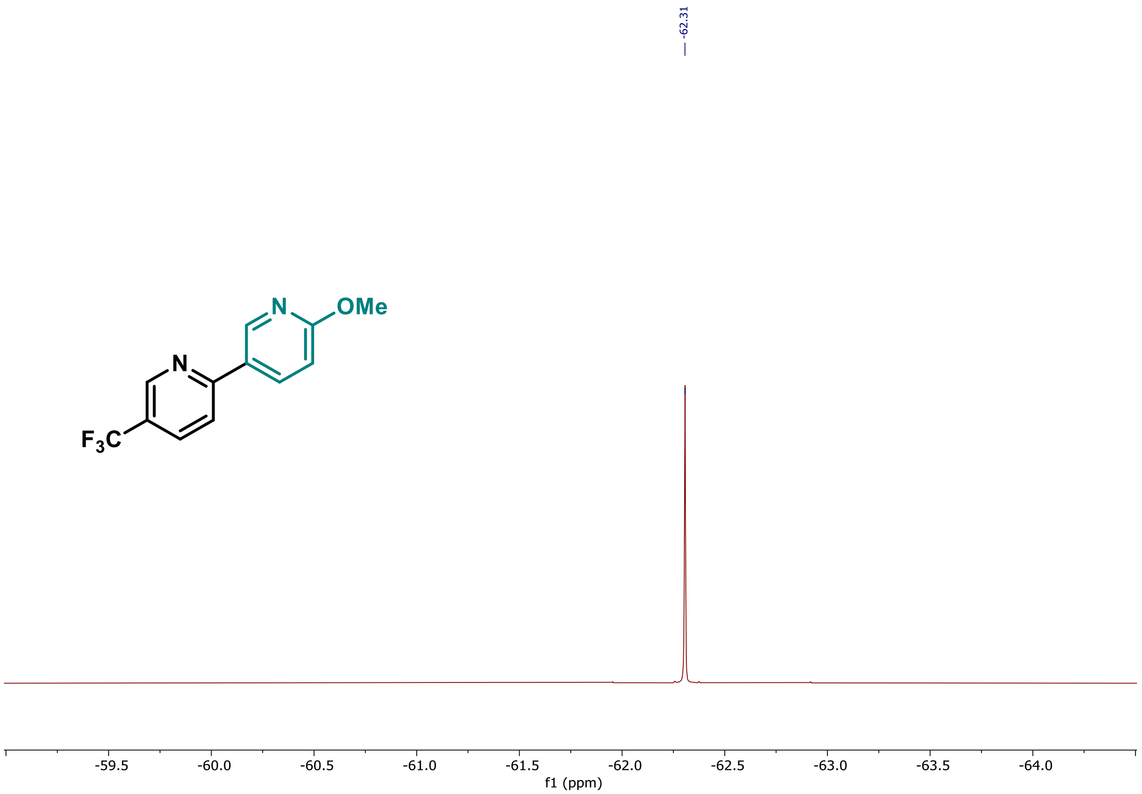

^{1}H NMR of 32 ($CDCl_3$, 400 MHz)

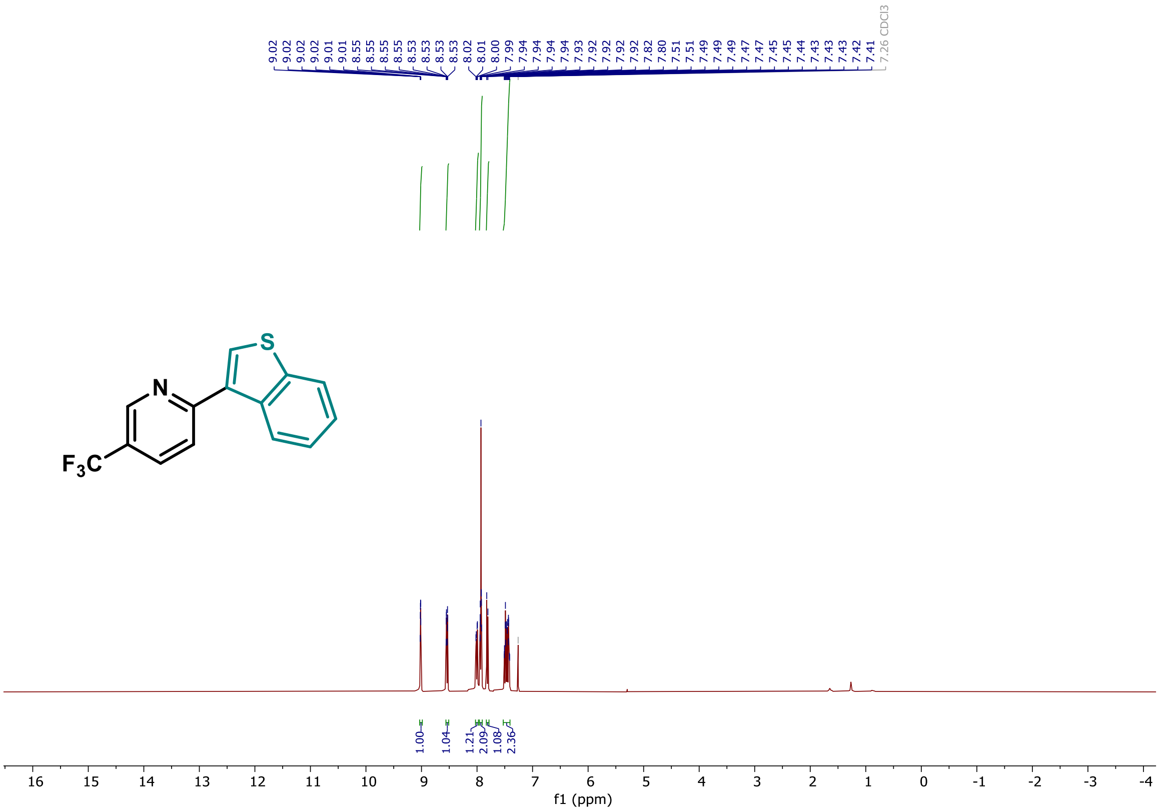

^{13}C NMR of 32 ($CDCl_3$, 101 MHz)

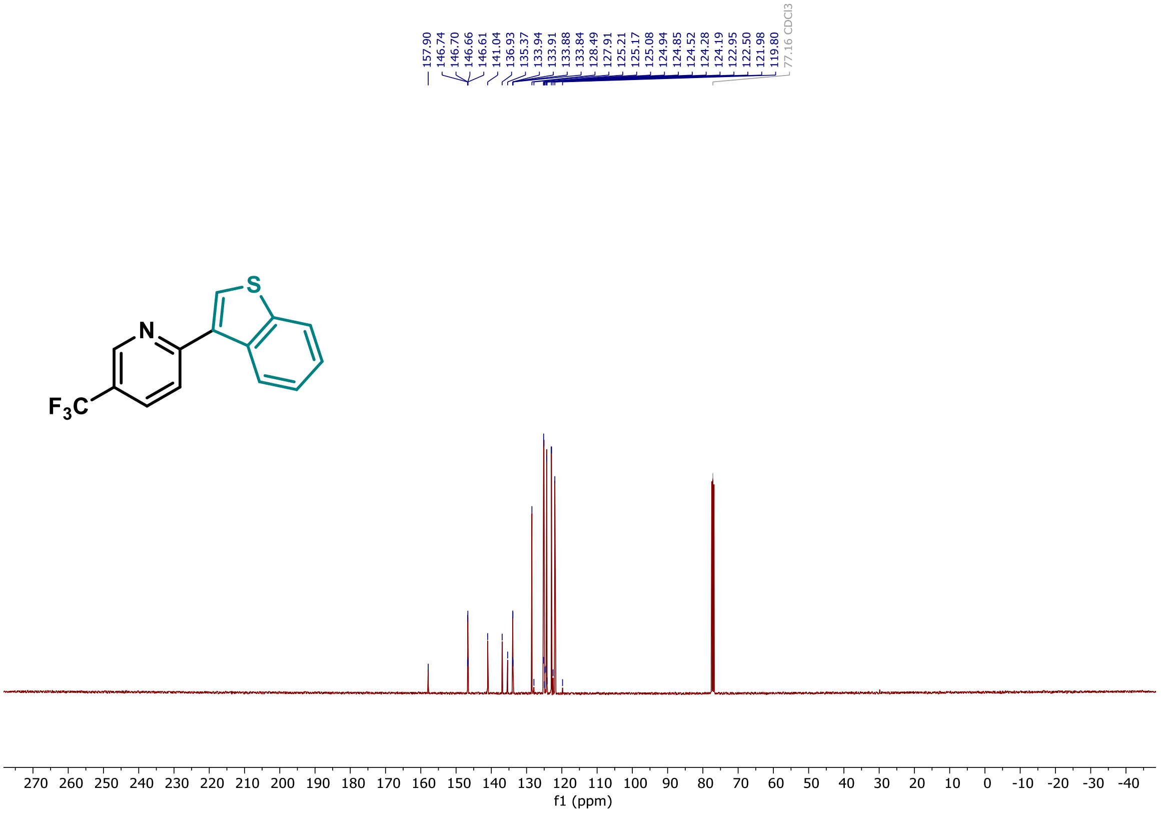

^{19}F NMR of 32 ($CDCl_3$, 282 MHz)

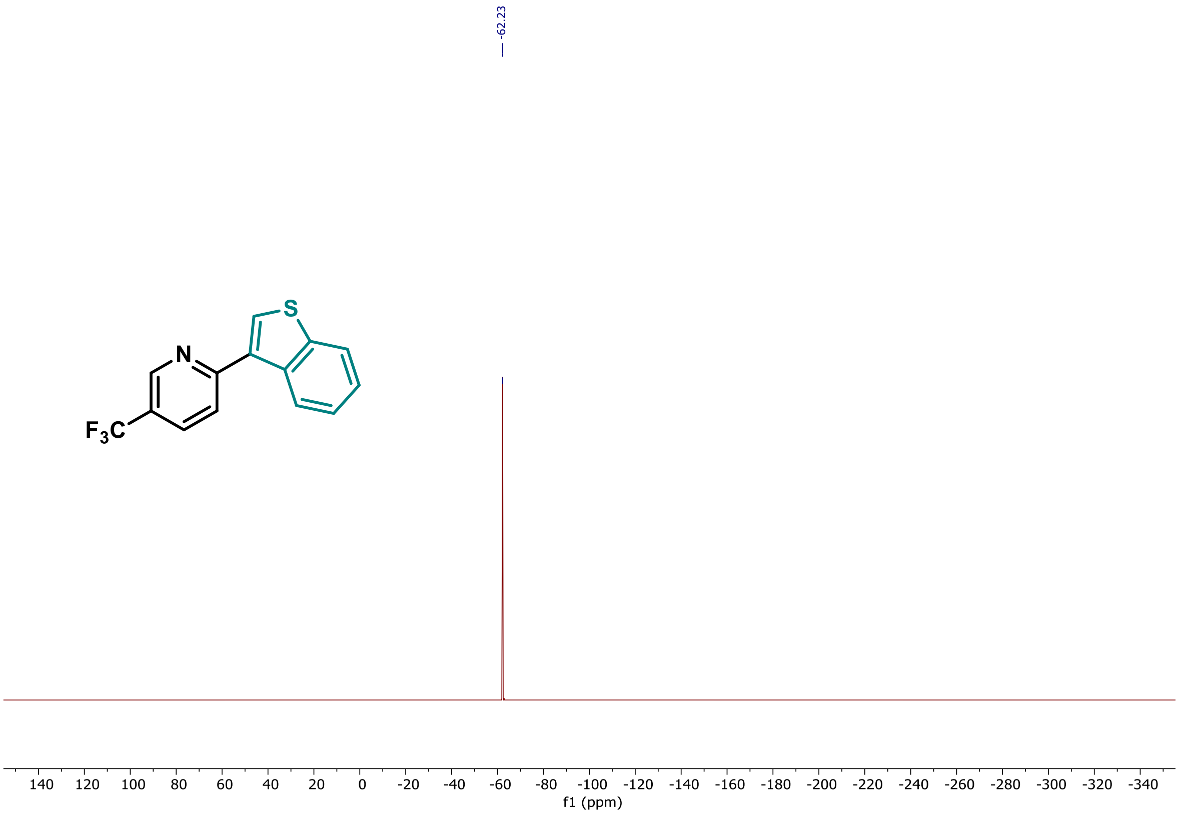

Supplement: Supplementary file 1 — Supporting Information [file ANIE-64-e202424051-s001.pdf]
